# Vesicle Transport in Plants: A Revised Phylogeny of SNARE Proteins

**DOI:** 10.1177/1176934320956575

**Published:** 2020-10-15

**Authors:** Xiaoyan Gu, Adrian Brennan, Wenbin Wei, Guangqin Guo, Keith Lindsey

**Affiliations:** 1 Ministry of Education Key Laboratory of Cell Activities and Stress Adaptations, School of Life Sciences, Lanzhou University, Lanzhou, China; 2 Department of Biosciences, Durham University, Durham, UK

**Keywords:** *Arabidopsis thaliana*, SNAREs, vesicle trafficking, membrane fusion, phylogenetics, Viridiplantae

## Abstract

Communication systems within and between plant cells involve the transfer of ions and molecules between compartments, and are essential for development and responses to biotic and abiotic stresses. This in turn requires the regulated movement and fusion of membrane systems with their associated cargo. Recent advances in genomics has provided new resources with which to investigate the evolutionary relationships between membrane proteins across plant species. Members of the soluble N-ethylmaleimide-sensitive factor attachment protein receptors (SNAREs) are known to play important roles in vesicle trafficking across plant, animal and microbial species. Using recent public expression and transcriptomic data from 9 representative green plants, we investigated the evolution of the SNARE classes and linked protein changes to functional specialization (expression patterns). We identified an additional 3 putative SNARE genes in the model plant *Arabidopsis*. We found that all SNARE classes have expanded in number to a greater or lesser degree alongside the evolution of multicellularity, and that within-species expansions are also common. These gene expansions appear to be associated with the accumulation of amino acid changes and with sub-functionalization of SNARE family members to different tissues. These results provide an insight into SNARE protein evolution and functional specialization. The work provides a platform for hypothesis-building and future research into the precise functions of these proteins in plant development and responses to the environment.

## Introduction

Communication between cells, and between compartments within cells, depends on membrane structure and function, and is fundamental to plant and animal growth and development and responses to environmental stresses. The regulated dynamics of membranes, and the associated proteins, is mediated by the membrane trafficking system, and is of great current interest to those interested in understanding the cell as an integrated system of signals and molecular responses. The membrane trafficking system in plant cells comprises the biosynthetic secretory pathway, the endocytic pathway and the vesicle transport pathway.^1^ The organelles involved in these processes use small membrane-enclosed transport vesicles to exchange molecular information.

**Figure 1.**
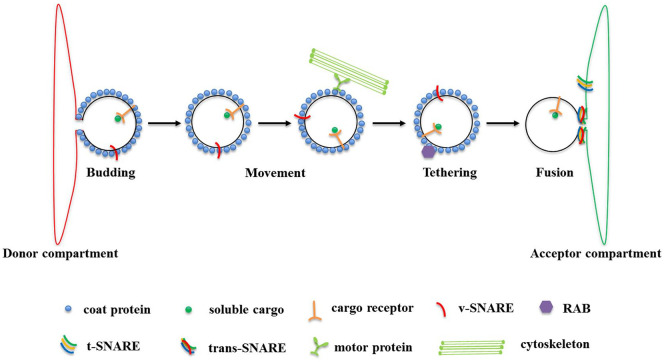
The vesicle trafficking pathway. Adapted from Cai et al.^7^

There are 4 essential steps in the vesicle trafficking system, namely budding, vesicle movement, tethering and fusion (Figure 1). During budding, coat proteins and dynamin-related GTPases in a donor compartment are used to form a vesicle and deform the local membrane until a vesicle is freed by scission. Cargo and vesicle (v)-SNAREs (soluble N-ethylmaleimide-sensitive factor attachment protein receptors) are incorporated into the budding vesicle by binding to coat subunits. In the movement phase, the freed vesicle moves towards the acceptor compartment by association with cytoskeletal motors. Molecular motors including kinesin and myosin have all been shown to be involved in this process.^2,3^ Then tethering and docking factors work in conjunction with Rab GTPases and SNARE proteins to tether the vesicle to their acceptor membrane.^4^ In the final, fusion step, a tetrameric target (t)-SNARE complex is formed from a single v-SNARE molecule (members of the synaptobrevin or VAMP family of proteins) and a trimeric target membrane t-SNARE complex (members of the syntaxin and SNAP-25 families) that allows vesicles to identify their target compartment and complete membrane fusion and cargo delivery.^5-9^

Previous reports have suggested there are 61 SNARE genes represented in the *Arabidopsis* genome,^10-12^ and the encoded proteins are located in different subcellular compartments and are essential for membrane fusion (Supplemental Figure 1; Supplemental Tables 1 and 2). SNARE proteins form a superfamily of small proteins of 100 to 300 amino acids that are characterized by a conserved protein motif called the SNARE domain, a membrane-anchor domain in the C terminus (TM) and a variable N-terminal domain.^6^ The SNARE domain contains a SNARE motif of 60 to 70 amino acids, and consists of a heptad repeat forming a coiled-coil structure, via hetero-oligomeric interactions.^6,8,13^ SNAREs can be classified on either a functional or structural basis. Classification on the basis of function puts SNAREs into vesicle-associated (v-SNARE) and target membrane-associated (t-SNARE) groupings.^14^ An alternative type of classification relies on amino acid sequence, though this results in a similar grouping to the v/t classification. Here, SNAREs are classified as either Q-SNAREs or R-SNAREs according to whether they contain a conserved glutamine (Q) or arginine (R) residue in the SNARE domain.^15^ t-SNAREs broadly correspond to Q-SNAREs, and v-SNAREs to R-SNAREs. SNAREs can be further subdivided into Qa-, Qb-, Qc-, Qb+c- and R-SNAREs, each with a hydrophobic SNARE domain.^6,16^ SNAREs include the vesicle-associated membrane protein (VAMP) known as synaptobrevin in animal systems, with a role in fusing synaptic vesicles to the plasma membrane.^17,18^

Most R-SNAREs localize to trafficking vesicles, and are attached via the C-terminal transmembrane (TM) domain. In plants, R-SNAREs form 3 major subfamilies, the Sec22-, YKT6- and VAMP7-like R-SNAREs.^12,19^ Vesicle resident R-SNAREs are typically designated VAMPs (vesicle-associated membrane proteins), and similar to other SNARE proteins have 3 functional domains, the N terminal domain, a SNARE motif and the transmembrane domain.^20^ VAMPs can be further classified into 2 subgroups, that is, with either a short or a long N-terminal sequence: the shorter VAMPs are termed ‘brevins’ and longer VAMPs, ‘longins’.^21^ Based on functions in synaptic exocytosis in mammalian systems, the shorter R-SNAREs are also termed synaptobrevins. However, all plant R-SNAREs are longins.^22,23^

In *Arabidopsis*, VAMP7-like R-SNAREs have been subjected to duplication into 2 gene families of 4 and 8 members respectively. The 4 VAMP71 group proteins are involved in endosomal trafficking;^10,24^ while the 8 VAMP72 group proteins are likely specific to the green plant lineage and represents R-SNARE components involved in secretion.^12,19^ VAMPs have roles as key proteins in abiotic stress tolerance (VAMP711, VAMP712),^25,26^ in gravitropic responses,^27^ in cell plate formation (VAMP721, VAMP722),^28-33^ in cytokinesis,^29,34^ in defence responses,^28,31,35^ in the transport of phytohormones (eg, Abscisic acid (ABA) and auxin) and in root formation and hormone response in plants.^36,37^ These functions are therefore dependent on correct membrane vesicle trafficking and its regulation. However, R-SNAREs may play a key role in determining the specificity in vesicle budding, and an important mechanism for SNARE localization is interaction with vesicle coats. For example, Springer & Schekman^38^ have shown that R-SNAREs may be components of the COPII vesicles that are involved in ER-Golgi transport. Rein et al^39^ found that R-SNAREs must be packaged into COPI vesicles during retrieval from the Golgi.

In this article, we analyze phylogenetic relationships between different classes of SNARE proteins across plant species across the plant kingdom, and summarize recent findings on the role of SNARE proteins in the model plant species *Arabidopsis thaliana*, to provide some insight into the evolution of these important proteins and to consider directions of research to understand better these proteins.

## Materials and Methods

### Phylogenetic analysis

To understand the phylogenetic relationships between different classes of SNAREs across plant species, we built a database of *Arabidopsis* SNARE-encoding genes and their homologues in 8 well annotated plant genomes (*Brachypodium distachyon* (grass), *Chlamydomonas reinhardtii* (alga), *Glycine max* (soybean), *Oryza sativa* (rice), *Physcomitrella patens* (moss), *Populus trichocarpa* (poplar), *Sorghum bicolor* (sorghum), *Vitis vinifera* (grapevine)). These species span the green plant kingdom (Viridiplantae), capturing various major taxonomic groupings in plants including: Embryophyta (all land plants excluding alga), Trachaeophyta (all vascular plants excluding alga and moss), Moncotyledoneae (rice, sorghum and grass), and Dicotelydoneae (*Arabidopsis*, soybean, poplar, grapevine). Each *Arabidopsis* gene on the TAIR database (https://www.arabidopsis.org) was searched to find its list of plant gene homologues that have previously identified by sequence-based methods through the PANTHER (Protein ANalysis THrough Evolutionary Relationships) curated database.^40^ The list of protein accession codes were searched on the UniProt database (https://www.uniprot.org) and fasta formatted amino acid coding sequences were downloaded (listed in Suppl. Table 3). Phylogenetic analysis was performed in R v3.5.3 (https://www.r-project.org) using the R packages: ape v5.3,^41^ ggtree,^42^ msa v1.4.3,^43^ phytools v0.6-99,^44^ phangorn v2.5.5^45^ and seqinr v3.6.1.^46^ The protein lists for each gene class were filtered by gene name to remove duplicated sequences, protein fragments, and allelic variants of the same gene. The remaining amino acid sequences for each gene class were aligned using the Expresso structural alignment algorithm of T-Coffee alignment suite (http://tcoffee.crg.cat/apps/tcoffee/^47^) that prioritizes alignment of protein structural motifs. The best amino acid evolutionary model for the aligned amino acid sequences was identified using the phangorn modelTest function, which was identified to be JTT+G+I in all cases. Maximum likelihood distances were calculated for the model using the phangorn dist.ml function and a best fit tree estimated using the phangorn optim.pml function with options appropriate for the JTT+G+I model. The resulting maximum likelihood trees were tested with 100 bootstrap iterations of the phangorn bootstrap.pml function. Trees were visualized using phytools and ggtree. Clades of related genes that were identified in each tree are highlighted with different colours. To understand the evolutionary relationships between the functional domains of different *Arabidopsis* SNAREs, the *Arabidopsis* amino acid sequences were analyzed to detect protein motifs with *P* < .05 using MEME software (http://meme-suite.org/tools/meme^48^). The amino acid sequences of the genes from other species were searched for regions that were significantly similar to these protein domains using FIMO software (http://meme-suite.org/doc/fimo.html^49^). Some short domains gave multiple hits for each gene so the significance threshold was adjusted from *P* < 1E × 04 to between *P* < 1E × 06 and *P* <1E × 08 that gave no more than 2 hits per domain per gene. The putative protein domains were identified when possible by manually checking the locations of these putative domains against the UniProt protein domain annotation of each *Arabidopsis* gene. Identified protein domain information was visualized alongside the phylogenetic trees using the gheatmap function of the R package ggtree to highlight the evolutionary gain and loss of motifs in each gene class.

### The expression of SNARE protein-coding genes in Arabidopsis

The expression data for 64 SNARE protein-coding genes in 79 organs and developmental stages of *Arabidopsis* were downloaded from TraVA (Transcriptome Variation Analysis, http://travadb.org/; Supplemental Tables 5 and Table 6).^50^ Expression levels of each gene across samples were scaled to have a mean of zero and standard deviation of one. The expression heatmap was generated using dChip software^51^ with the default settings of paired Pearson correlation distances as distance and centroid linkage method for building clusters.

### Relationship between genetic distance and expression differences

Paired Pearson correlation distances were calculated from the TraVA gene expression data using the cor.dist function of the R package bioDist v1.58.0.^52^ Coding sequences of each class of SNARE genes were aligned using webPRANK,^53^ a phylogeny-aware alignment algorithm that maintains the correct reading frame. Maximum likelihood trees were constructed for each class as described above but using the optimal GTR+G+I DNA model of evolution instead. Paired gene maximum likelihood genetic distances were extracted from this tree using the R ape function cophenetic.phylo. Sub-functionalization within each gene class was tested with Mantel tests to investigate relationships between genetic and expression distances using the R phytools multi.mantel function. For the between gene class sub-functionalization tests, mean, standard deviation, and sample size values were calculated for the genetic and expression distance matrices for each gene class. The relationship between mean gene class genetic and expression distance weighted by sample size was then tested with linear models in R. Neo-functionalization was tested by measuring paired gene non-synonymous to synonymous base change ratios (Dn/Ds) for each pair of genes within each webPRANK aligned gene class using the R seqinr kaks function assuming that amino acid changes contribute to changes in protein function. Gene pairs that gave valid Dn/Ds values greater than zero and less than 9 were considered. Mantel tests were used to investigate relationships between Dn/Ds and expression distances. Between gene class neo-functionalization tests were performed with linear models of gene class means weighted by sample size.

## Results

### Phylogenetic analysis of plant SNARE proteins

**Figure 2.**
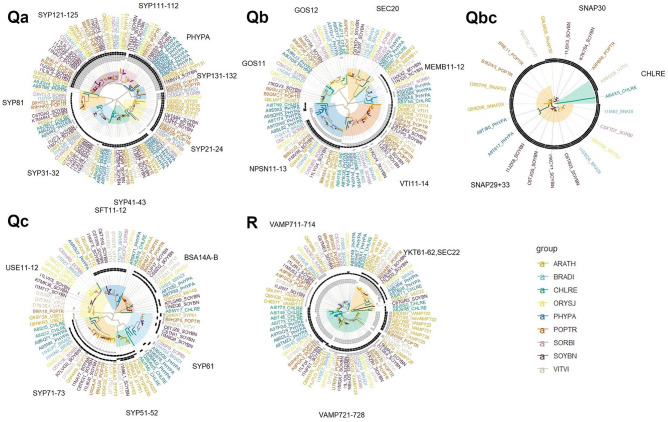
Maximum likelihood trees of all 5 classes of SNARE genes in 9 different species representing all green plants. Branch tips have been labelled according to UniProt gene name and 5 letter species abbreviation, except for *Arabidopsis* where the gene name has been provided instead. Branches and tip labels have been coloured according to species: *Arabidopsis thaliana* (ARATH); *Brachypodium distachyon* (BRADI); *Chlamydomonas reinhardtii* (CHLRE); *Oryza sativa* (ORYSJ); *Physcomitrella patens* (PHYPA); *Populus trichocarpa* (POPTR); *Sorghum bicolor* (SORBI); *Glycine max* (SOYBN); *Vitis vinifera* (VITVI). Clades representing a predominance of 1 *Arabidopsis* gene type have been highlighted with different colours. The rings of bands correspond to protein domains identified in each gene. Black domains correspond to domains annotated as SNAREs and grey bands correspond to other domains. Domains are ordered from the centre to the edge of the circle approximating their N to C terminal order.

A search of PANTHER homologues of *Arabidopsis* SNARE genes led to the identification of 3 new putative *Arabidopsis* SNARE homologues; 2 Qc-SNARE homologues, AT1G16225 and AT1G16230; and an R-SNARE homologue, AT3G25013, and many other similar genes across the 8 other target species. The phylogenetic trees of each SNARE gene class are presented in Figure 2. Identified protein domains are summarized in the trees and in Supplemental Table 4. The main evolutionary patterns that the phylogenetic analyses reveal in each gene class are discussed in the following sections.

For the Qa-SNAREs, we identified 8 distinct clades that are highly conserved across plants (Figure 2). Some clades can be traced back into more basal green plant representatives than others and clades also reveal a progressive increase in protein domain complexity. Each of the clades labelled SYP31-32, SYP41-43, and SYP81 could be traced back to the most ancient Viridiplantae grouping including alga. The basal SYP81 clade gains 1 recognisable tSNARE domain from the Trachaeophyta onwards, missing this domain in alga and moss. The basal SYP31-32 clade gains the same tSNARE domain earlier from the Embryophyta onwards, missing it only in the alga. The SYP41-43 clade has this original tSNARE domain throughout and gains a second tSNARE domain from the embryophyta onwards. Some later gene duplication has taken place in these clades with, for example, 2 gene copies in the moss for SYP31-32 and SYP41-43 and up to 4 gene copies of SYP32 present in soybean. The clade SYP21-24 is the next most ancient as it can be traced back to all Embryophyta. This clade is characterized by 4 gene copies in both moss and *Arabidopsis* and mostly contains the 2 tSNARE domains and a new coiled coil protein domain. These 4 basal clades show sporadic evidence for a transmembrane domain. The remaining clades, SYP131-132, PHYPA, SYP111-112 and SYP121-125, are more closely related to each other and are only found in Embryophyta. There is a diverse 7 member moss-specific PHYPA clade, suggesting the evolution of multiple specialist functions for this SNARE gene family in moss. These results are consistent with the recent phylogeny study of SYP1 subfamily of Qa-SNAREs by Slane et al^54^ The other clades, SYP111-112, SYP121-125, SYP131-132 are only found in Trachaeophyta. These clades have diversified into many gene members with evidence of gene duplication within soybean with 8 and 5 gene copies in the SYP121-125 and SYP111-112 clades, respectively. These 4 clades mostly all contain the transmembrane domain, the 2 tSNARE domains and a 3 preceding coiled coil domains, the SYP-121-125 gaining an extra fourth coiled coil domain. Genes that lack several of these domains, such as B9I5W3_POPTR and K7KQY1_SOYBN have relatively long branch lengths and might be recent pseudogenes copies. Coiled coil domains enable protein interactions and interestingly, the *Arabidopsis* genes SYP121 and SYP122 function as negative regulators of the programmed cell death reaction, the salicylic acid, jasmonic acid and ethylene signalling pathways,^55^ and the membrane traffic mediated by SYP121 and SYP122 are associated differentially with lots of cargo proteins.^56^ The *Arabidopsis* genes SYP123, SYP124 and SYP125 that function to specify root hair and pollen tissues, tissues not found in algae or moss, are specific to Trachaeophyta.^57-60^

The Qb-SNAREs cluster into 6 clades (Figure 2). All of the clades are present across all Viridiplantae, except GOS11 that is present in all Embryophyta, suggesting that the functions of each of the Qb-SNARE clades are ancient and essential. Gene diversification is evident in these clades. Multiple moss gene copies are present in all clades, possibly reflecting a need for more Qb-SNAREs with increasing multicellularity and adaptation to land. The largest clade is VTI11-14, with multiple copies found in *Arabidopsis* (4 gene copies plus an isoform), soybean (5 copies), poplar and moss (4 copies each), grapevine (3 copies), and grass (2 copies). The smallest group of Qb-SNAREs is the MEMB11-12 clade, which is nonetheless found in multiple copies in *Arabidopsis* (2 copies), soybean (2 copies) and moss (3 copies). This clade appears to be missing from grass and poplar, but this may be an artefact of database incompleteness. In terms of protein domains, the clades GOS11, GOS12 and SEC20, show no recognisable domains. The MEMB11-12 clade contains the tSNARE domain from the Qa class, and Trachaeophyta members also contain a shared coiled coil domain. Only 2 moss genes show a recognisable transmembrane domain. The VTI11-14 clade contains this tSNARE and coiled coil domain with an extra coiled coil domain in Embryophyta members of the clade. Some members have a transmembrane domain. The NPSN11-13 clade has an algal member with only the tSNARE domain, 5 moss members that show a mix of 2 tSNARE and 2 coiled coil domains that are distinct from the other clades in this class, and a remaining Trachaeophyta members all show these 4 domains and a transmembrane domain.

For the Qc-SNAREs, there are 6 clades, all except USE11-12 and SYP51-52 that can be traced back to all Viridiplantae (Figure 2). The USE11-12 clade traces to Embryophyta but the moss gene is not at the expected basal position. This clade contains just 1 recognised coiled coil domain in the Dicotyledoneae; *Arabidopsis*, grapevine, and soybean. The SFT11-12 clade contains 2 tSNARE domains starting from the Trachaeophyta. The BSA14A-B clade contains one of these tSNARE domains in all Viridiplantae and a second distinct tSNARE domain in Trachaeophyta members only. The SYP61 clade contains the same set of domains as the prior clade but not all domains are reliably identified in all clade members. This clade also sometimes shows a transmembrane domain. Interestingly, the SYP51-52 clade is found only in some Dicotyledoneae including soybean, grapevine, and *Arabidopsis*. There are 4 *Arabidopsis* gene copies in this clade, including 2 previously uncharacterized genes, At1g16225 and At1g16230. These genes are mainly expressed in root tissue. This clade contains the widespread tSNARE domain, the tSNARE domain of BSA14A-B and SYP61 and a third distinct tSNARE domain in most members. The largest clade is SYP71-73 with 2 copies each for alga, moss, and grass, and poplar, 3 copies in grapevine, and *Arabidopsis* and 5 copies in soybean. This clade contains a distinct coiled coil domain and the widespread tSNARE domain in all Viridiplantae and the SYP61-like tSNARE domain in all Embryophyta. An occasional transmembrane domain is identified in this clade.

The Qb+cSNAP SNARES can be divided into 3 clades, CHLRE SNAP29, SNAP29+SNAP33 but these clades are relatively poorly supported (Figure 2). The CHLRE clade contains highly divergent alga gene and a grapevine gene. The small SNAP30 clade contains Dicotyledoneae *Arabidopsis*, poplar and 2 soybean genes. The final larger SNAP29+33 group contains Embryophyta members but the divergent 2 moss genes are not in the expected basal position. As with all other gene classes examined, multiple gene copies were found in many species, most notably soybean with 6 copies. Most class members contained 3 tSNARE domains corresponding to Qb and Qc class tSNARE domains.

The R-SNAREs form 3 large clades distinguished by YKT61-62+SEC22, VAMP711-714, and VAMP721-728 encoding genes that are each conserved in all Viridiplantae (Figure 2). The smallest clade is YKT61-62+SEC22, which is represented at least once in all studied species. The base of this clade is defined by a pair of divergent alga genes, *Arabidopsis* SEC22, and a soybean gene C6TGR3. These genes except for soybean contain a widespread vSNARE domain. The remaining YKT61-62 subclade is found in all Viridiplantae, suggesting its essential cell trafficking role. This clade contains mostly the widespread vSNARE domain, a second vSNARE domain and up to 3 longin domains. The algal gene A8J6T1 contains just 1 of the 3 longin domains. VAMP711-714 is the next largest clade, again with representatives in all studied species, including probable gene duplicates in poplar (5 copies), soybean (4 copies), *Arabidopsis* (4 copies), and moss (3 copies). This clade contains the widespread vSNARE domain and 3 longin domains that are distinct from the previous clade. Only the algal gene A8J924 contains a single central longin domain. Dicotyledoneae members of the VAMP711-13 subclade mostly contain a transmembrane domain also. The largest clade is represented by VAMP721-728 with multiple members from all Viridiplantae. Gene expansion is likely to have occurred within *Arabidopsis* for this clade with VAMP-721, 722, 723, 725, and 726 all forming a monophyletic subclades. The basal alga and moss also show monophyletic clusters of duplicated genes (clusters of 4 genes each). It might be that the role of this clade of genes in SNARE complex formation has allowed for repeated pattern of redundant gene duplication within species. This clade includes a divergent *Arabidopsis* gene AT3G25013 that was designated as a new putative R-SNARE gene by PANTHER. This clade contains the same vSNARE and 3 longin domains as the VAMP711-14 as well as up to 2 transmembrane domains from Embryophyta onwards. Genes missing several of these domains have relatively long branch lengths and are probably pseudogenes.

### The expression of SNARE protein-coding genes in Arabidopsis

**Figure 3.**
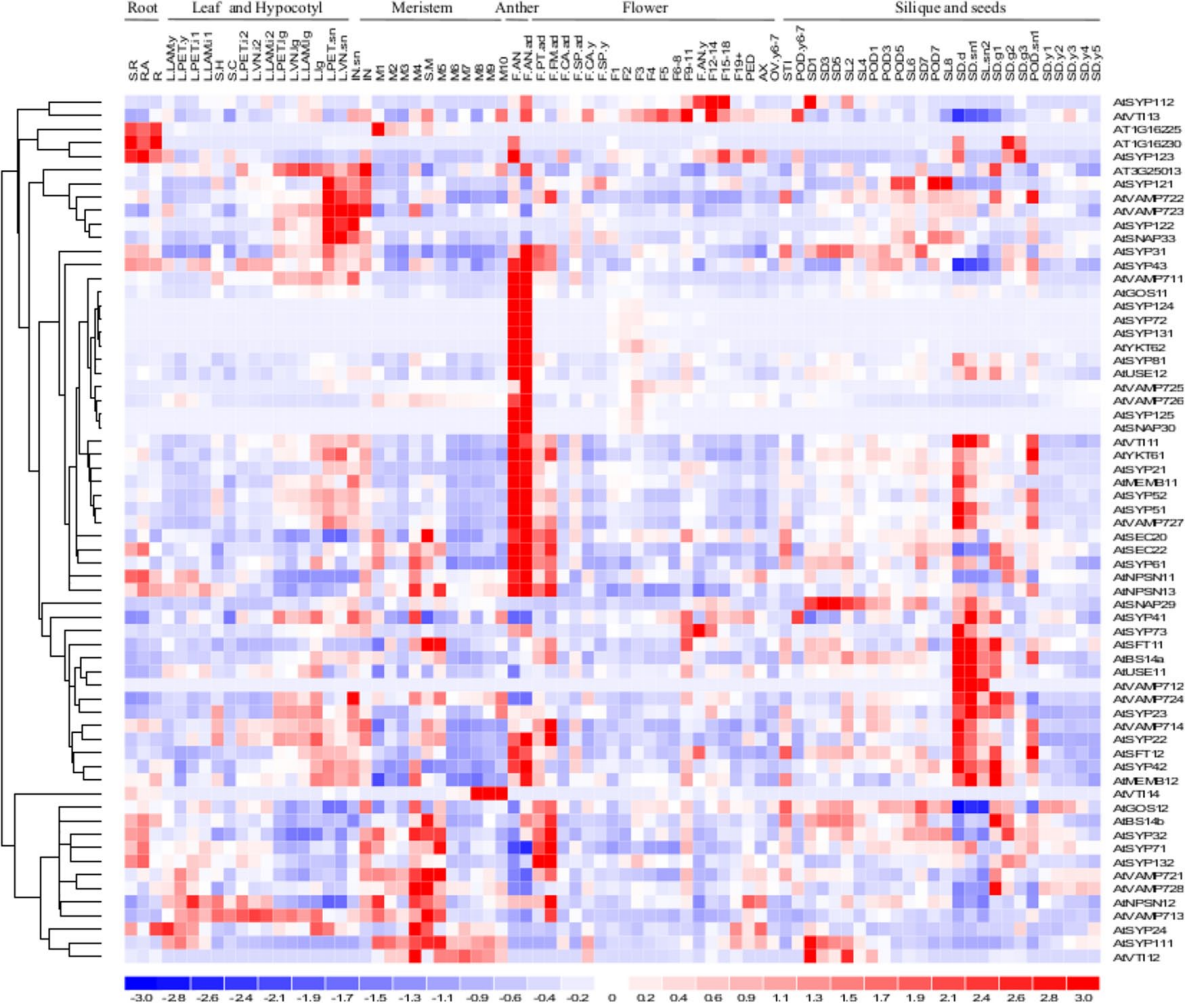
Expression heat map of SNARE genes in different organs and developmental stages of *Arabidopsis*. Expression levels of each gene across samples were scaled linearly to have a mean of 0 and a standard deviation of 1. Samples were numbered as in Supplemental Table 5.

The expression of 64 SNARE protein-coding genes in 79 organs and developmental stages of *Arabidopsis* is shown in Figure 3 (see Methods for details). SNARE-encoding genes show a wide range of expression levels and distinct regulation during *Arabidopsis* development. *Arabidopsis* SNARE genes are clustered according to their expression patterns in root, leaf, meristem, anther, flower, silique and seeds. Two SNARE-encoding genes (*SYP112* and *VTI13*) are abundantly expressed in flower. Five SNARE transcripts (*SYP121, VAMP722, VAMP723, SYP122* and *SNAP33*) are abundant in leaf and internode. Twenty SNARE genes (*SYP123, SYP31, SYP43, VAMP711, GOS11, SYP124, SYP72, SYP131, YKT62, SYP81, USE12, VAMP725, VAMP726, SYP125, SNAP30, SEC20, SEC22, SYP61, NPSN11* and *NPSN13*) are highly expressed in the anther. Interestingly, the *Arabidopsis* genes *SYP123, SYP124, SYP125* and *SYP131* might be important for exocytosis during pollen tube growth.^54,57-60^ SEC22 is essential for gametophyte development and maintenance of Golgi-stack integrity.^61^ Seven SNARE gene transcripts (*VTI11, YKT61, SYP21, MEMB11, SYP52, SYP51* and *VAMP727*) are abundant in anther and seed. *SNAP29* is abundant in the inflorescence meristem and seeds. Nine (*SYP41, SYP73, SFT11, BS14a, USE11, VAMP712, VAMP724, SYP23, VAMP714*) are strongly expressed in the silique and seeds, and 5 (*GOS12, BS14b, SYP32, SYP71* and *SYP132*) are abundant in meristem and flower. *VTI14* is abundant in inflorescence meristem. Four SNARE genes (*SYP22, SFT12, SYP42* and *MEMB12*) are strongly expressed in anther, silique and seeds. Seven (*VAMP721, VAMP728, NPSN12, VAMP713, SYP24, SYP111* and *VTI12*) are strongly expressed in meristems.

### Relationship between genetic distance and expression differences

Large gene families like SNAREs usually arise through gene duplication.^62^ Redundant duplicated genes tend to have a limited evolutionary lifespan unless they acquire new useful functions. Gene paralogues can acquire functional diversity either through sub-functionalization when different tissues or developmental stages come to predominantly express only 1 of the available gene paralogues, or neo-functionalization, when relaxed evolution during the redundant phase allows gene copies to explore adaptive space and acquire novel functions.^63,64^

**Figure 4.**
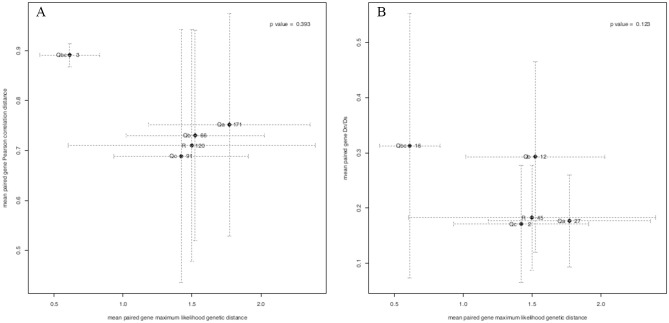
Relationships between (A, left) genetic distance versus expression distances, and (B, right) Dn/Ds ratios versus expression distances across SNARE gene classes in *Arabidopsis*. Filled diamonds show mean values for SNARE gene classes. Dotted bars show standard deviations. Gene class names and the number of values assessed are plotted to the left and right of each point, respectively.

We explored the first of these hypotheses of gene family diversification for SNARE genes by comparing genetic distances to expression differences within and between each gene class. If duplicated genes gradually accumulate expression differences under weak or absent divergent selection, then a significant positive relationship between genetic distance and expression distance would be expected within each gene class. More rapid accumulation of expression differences between duplicated genes would mask this null relationship. For comparisons between gene classes, we might expect that larger families would show more sub-functionalization compared to smaller families. Analysis of the relationships between genetic and expression distances suggested that no significant positive relationships were found for any gene class suggesting that the hypothesis of rapid sub-functionalization of gene duplicates cannot be ruled out within gene classes (Supplemental Figure 2). No relationship was found between genetic and expression distance across gene families failing to support the hypothesis of sub-functionalization at this level (Figure 4).

We explored the second hypothesis of gene diversification by neo-functionalization. According to the neo-functionalization hypothesis, divergent selection for amino acid changes between duplicated genes would weaken an otherwise longer-term positive relationship for amino acid changes to accumulate over longer periods of time. The results of Mantel tests showed that, except for Qa, there were no significant positive relationships between Dn/Ds and expression distances/genetic distances at either the within-or between gene class levels (Supplemental Figures 3–5). The largest gene classe, Qa, showed a positive linear relationship between genetic distance and Dn/Ds (*r*^2^ = 0.61, β = 6.44, *P* = 0.005) supporting a null hypothesis of gradual functional change (Supplemental Figure 4). The other gene families did not show significant positive linear relationships suggesting that some gene pairs in these families might accumulate putatively functional amino acid differences at a faster rate than others. The need for coordinated interactions between different R class genes in the SNARE core complex might contribute to their relatively rapid amino acid change.

## Discussion

During the last few years there have been advances in our understanding of the functions of SNARE proteins, especially in the membrane fusion and vesicle transport pathways. However, the functional redundancy between SNAREs makes it hard to define precisely their fusion specificity in relation to specific cellular or developmental events. For example, functional redundancy exists between VTI11 and VTI12,^65,66^ SYP121 and SYP122,^67,68^ and VAMP721 and VAMP722 R-SNAREs.^28^ Homozygous double mutants of each show embryo lethal or severe growth defects, but single gene knockout mutations do not show any obvious phenotypes. SYP22 and SYP23,^69^ VAMP711, VAMP712 and VAMP713^70^ may either form distinct SNARE complexes or they could have overlapping functions for specific developmental roles. The redundancy between SNAREs makes it difficult to identify specific SNARE functions if we use traditional experimental approaches.^71^

Our phylogenetic analysis provides insights into patterns and drivers of SNARE family gene diversification across green plants. Each SNARE class could be broken down into subclades, many of which had genes representing all studied species, attesting to their ancient evolutionary origins. Some sets of homologous genes including: Qa: SYP21-24, SYP31-32, SYP41-43, SYP81; Qb: SEC20, MEMB11-12, VTI11-14, NPSN11-13; Qc: SYP61, SYP71-73, BS14a-b, SFT11-12; and R: SEC22 plus YKT61-62, VAMP711-714, VAMP721-728 are found in all species examined, indicating that the SNARE complex was inherited vertically throughout all Viridiplantae.^12^ Many of these subclades were diverse, with relatively high numbers of gene duplications within some species. Species specific gene duplications frequently occurred in plants. The phylogenetic trees show that many SNARE subclades including: Qa SYP1; Qc USE11-12; and Qc SYP51-52, appeared during the evolution of multicellular land plants (Embryophyta), with a similar story for syntaxins in higher animals.^12^ These more recent subclades contain an increased number of protein domains involved in protein-protein interactions and associated with cell membranes. Therefore 1 reason for the diversification of SNAREs might be related to the increased complexity of inter- and intracellular communication systems that are associated with multicellularity. For other SNARE classes, for example: Qa-SNAREs, Qb-NPSN11-13, and R-VAMP721-728, it is less clear why basal green plants such as moss have species-specific subclades with as many as 7 genes. Understanding these phylogenetic relationships will help address our understanding of the evolution of SNARE function. Gene duplications in *Arabidopsis* seem to be more associated with sub-functionalization to different tissue types than neo-functionalization in terms of the accumulation of amino acid changes, as no associations were found between genetic and expression distance.

Some information on the functions of SNAREs in different tissues is available. Depending on the specific intracellular trafficking process, different v-/t-SNARE complexes are correspondingly formed.^18^ This specificity of t-SNARE and v-SNARE complex formation ensures that vesicles are targeted to the correct compartment and induce membrane fusion.^72^ For example, SYP111 takes part in membrane fusion events forming the cell plate and the transport of secretory vesicles at the plasma membrane.^34,73-75^ SYP122 plays a role in cell wall deposition and in tethering of donor and target membrane.^76^ SYP81, SYP31, SYP32, GOS11, GOS12, MEMB11, SFT11, SFT12, BS14a, BS14b, SEC22, YKT61 and YKT62 mediate anterograde traffic between ER and Golgi and retrograde traffic within the Golgi apparatus.^5,61,77-82^ SEC20, MEMB12, USE11, USE12, SYP71, SYP72 and SYP73 mediate retrograde traffic from Golgi to ER for protein recycling and balance maintenance.^12,29,83-86^ SYP51 and SYP52 take part in direct membrane transport from ER to tonoplast and Golgi to mediate vesicle trafficking.^87,88^ VAMP714 interacts with SYP121 and SYP22 to mediate vesicle transport from the Golgi apparatus to the vacuole.^89,90^

These cell trafficking functions are important mediators of environmental responses. Thus, Q-SNAREs have roles as key proteins in gravitropic responses (VTI11, VTI14),^27,65^ in cytokinesis (SYP31, SYP32, NPSN11, NPSN12, NPSN13),^4,10,91-93^ in auxin homeostasis (VTI11, SYP41, SYP42, SYP43),^94-99^ and in cell plate formation and pathogen resistance (SYP121, SYP131, SYP132, SNAP33).^35,37,67,100-106^

SYP121 mediates vesicle fusion at the *Arabidopsis* plasma membrane, and binds the K^+^ channels voltage sensors to coordinate membrane trafficking with K^+^ uptake for growth.^107^ Karnik et al.^30,108,109^ and Zhang et al.^110^ found that SM protein SEC11 binds and selectively regulates secretory traffic mediated by N terminus of SYP121 and is important for assembling and recycling of the SNARE during membrane fusion. And Sec1/Munc18 proteins interact with some SNARE proteins to form the exocyst complex, which initiates membrane fusion.^111,112^ Moreover, Waghmare et al.^56^ found that the membrane traffic mediated by SYP121 and SYP122 is associated differentially with lots of cargo proteins.

Xue et al^26^ found that VAMP711 regulates ABA-mediated inhibition of PM H+-ATPase activity and drought stress response by regulating stoma closure. Zhang et al^19,75^ found that VAMP721 and VAMP722 interact with the same K+ channels and that this interaction suppresses channel activity, and VAMP721 assembles with SYP121 to coordinate K+ channel gating during SNARE assembly and vesicle fusion. Kim et al.^113^ found that CALRETICULIN 1 (CRT1) and CRT2 are critical components in the accumulation of VAMP721 and VAMP722 during ER stress responses. Zhang et al.^114^ found that SYP22 and VAMP727 mediate the BRI trafficking to PM.

These studies show that SNARE proteins likely function in trafficking at most membranes, but especially at the vacuole and plasma membrane. And VAMP727 forms a complex with SYP22, VTI11, SYP121 and SYP51 to drive membrane fusion and vesicle transport pathways between PVCs and vacuoles.^32,33,115^ Löfke et al.^99^ found that auxin can increase the amount of SNARE proteins in the vacuolar membrane. Shirakawa et al.^116^ found that SYP22 functions in the polarized localization of the auxin efflux carrier PIN1 in the *Arabidopsis* leaf. Xia et al^117^ found that SYP132 and associated endocytosis play significant roles in auxin-regulated H^+^-ATPase traffic and associated functions at the plasma membrane. Moreover, we have recently found that AtVAMP714 is also required for the exocytic localization of PIN1 and PIN2 proteins to the plasma membrane via the Golgi, and for polar auxin transport; and the related genes *VAMP711, VAMP712* and *VAMP713* are also inducible by auxin.^118^

## Conclusion

The analysis presented in this paper shows that SNARE proteins likely play very important roles in plant development, with functions being identified in auxin transport and signalling. Phylogenetic studies show that SNARE diversification is associated with the evolution of multicellularity in plants, therefore providing new insights into the functions of SNARE proteins in vesicle transport pathways. This phylogenetic context can be combined with future molecular genetic findings for greater insights. Knowing where and how those membrane protein complexes originate and are destined in the cell, and their dynamics and relationship with membrane composition that determines for example transporter polarity in a tissue-specific manner,^119^ will improve our knowledge of the importance of SNARE complexes in vesicle trafficking pathways and in a range of developmental and stress response pathways in higher plants.

## Supplemental Material

00f8b8c046636_EvoBioRevSupplTable5y_xyz466362fa485fb – Supplemental material for Vesicle Transport in Plants: A Revised Phylogeny of SNARE Proteins Click here for additional data file. Supplemental material, 00f8b8c046636_EvoBioRevSupplTable5y_xyz466362fa485fb for Vesicle Transport in Plants: A Revised Phylogeny of SNARE Proteins by Xiaoyan Gu, Adrian Brennan, Wenbin Wei, Guangqin Guo and Keith Lindsey in Evolutionary Bioinformatics

2873cc8b46636_EvoBioRevSupplTable4_xyz466366e8025bf – Supplemental material for Vesicle Transport in Plants: A Revised Phylogeny of SNARE Proteins Click here for additional data file. Supplemental material, 2873cc8b46636_EvoBioRevSupplTable4_xyz466366e8025bf for Vesicle Transport in Plants: A Revised Phylogeny of SNARE Proteins by Xiaoyan Gu, Adrian Brennan, Wenbin Wei, Guangqin Guo and Keith Lindsey in Evolutionary Bioinformatics

3185449246636_EvoBioRevSupplTable1_xyz466364bc83175 – Supplemental material for Vesicle Transport in Plants: A Revised Phylogeny of SNARE Proteins Click here for additional data file. Supplemental material, 3185449246636_EvoBioRevSupplTable1_xyz466364bc83175 for Vesicle Transport in Plants: A Revised Phylogeny of SNARE Proteins by Xiaoyan Gu, Adrian Brennan, Wenbin Wei, Guangqin Guo and Keith Lindsey in Evolutionary Bioinformatics

529d24f646636_EvoBioRevSupplTable6_xyz466362abe5173 – Supplemental material for Vesicle Transport in Plants: A Revised Phylogeny of SNARE Proteins Click here for additional data file. Supplemental material, 529d24f646636_EvoBioRevSupplTable6_xyz466362abe5173 for Vesicle Transport in Plants: A Revised Phylogeny of SNARE Proteins by Xiaoyan Gu, Adrian Brennan, Wenbin Wei, Guangqin Guo and Keith Lindsey in Evolutionary Bioinformatics

ef0a8cf646636_EvoBioRevSupplTable3y_xyz466366d196b73 – Supplemental material for Vesicle Transport in Plants: A Revised Phylogeny of SNARE Proteins Click here for additional data file. Supplemental material, ef0a8cf646636_EvoBioRevSupplTable3y_xyz466366d196b73 for Vesicle Transport in Plants: A Revised Phylogeny of SNARE Proteins by Xiaoyan Gu, Adrian Brennan, Wenbin Wei, Guangqin Guo and Keith Lindsey in Evolutionary Bioinformatics

f763b0b446636_EvoBioRevSupplTable2_xyz46636386aed71 – Supplemental material for Vesicle Transport in Plants: A Revised Phylogeny of SNARE Proteins Click here for additional data file. Supplemental material, f763b0b446636_EvoBioRevSupplTable2_xyz46636386aed71 for Vesicle Transport in Plants: A Revised Phylogeny of SNARE Proteins by Xiaoyan Gu, Adrian Brennan, Wenbin Wei, Guangqin Guo and Keith Lindsey in Evolutionary Bioinformatics

Supplementary_Figures_1-5_Gu_et_al. – Supplemental material for Vesicle Transport in Plants: A Revised Phylogeny of SNARE Proteins Click here for additional data file. Supplemental material, Supplementary_Figures_1-5_Gu_et_al. for Vesicle Transport in Plants: A Revised Phylogeny of SNARE Proteins by Xiaoyan Gu, Adrian Brennan, Wenbin Wei, Guangqin Guo and Keith Lindsey in Evolutionary Bioinformatics
