# Supplementary material for: Vesicle Transport in Plants: A Revised Phylogeny of SNARE Proteins
Source: Evol Bioinform Online. 2020 Oct 15;16:1176934320956575. doi: 10.1177/1176934320956575 (PMC7573729; doi:10.1177/1176934320956575)
Supplement: 00f8b8c046636_EvoBioRevSupplTable5y_xyz466362fa485fb – Supplemental material for Vesicle Transport in Plants: A Revised Phylogeny of SNARE Proteins [file 00f8b8c046636_EvoBioRevSupplTable5y_xyz466362fa485fb.pdf]

	A	B	C	D	E	F	G	H	I	J	K	L	M	N	O	P	Q	R	S	T	U	V	W	X	Y	Z	AA	AB
1	Symbol	S.R	R.A	R	L.LAM.y	L.PET.y	L.PET.i1	L.LAM.i1	S.H	S.C	L.PET.i2	L.VN.i2	L.LAM.i2	L.PET.lg	L.VN.lg	L.LAM.lg	L.lg	L.PET.sn	L.VN.sn	IN.sn	IN	M1	M2	M3	M4	S.M	M5	M6
2	AtVAMP72	2269	2644	2270	2718	3689	3083	2436	2598	1911	2382	2355	2454	1955	1716	1550	1967	2069	1839	2443	3607	3727	3534	3265	4466	6305	5870	2248
3	AtVAMP72	36	54	80	238	390	275	134	42	46	65	45	31	21	19	29	20	7	10	11	108	482	303	290	476	396	350	273
4	AtSYP111	1331	1550	1101	4866	6046	4173	2840	319	1213	773	685	505	271	147	26	116	37	40	30	877	6771	6315	5071	7356	8411	8624	4133
5	AtSYP125	2	2	5	4	0	0.51	5	0	3	0	0.62	0	0	0.47	2	0	1	4	0.98	0	0	0	0	0	0	0	0
6	AtSEC22	1127	1322	837	675	747	823	797	485	730	737	738	730	666	626	751	669	938	835	731	846	1148	928	873	1233	978	1172	604
7	AtSNAP30	0	0	0.8	0	0	0	0	0	2	0	0	0	0.47	0.47	0	1	3	2	4	0	0	0	0	0	0	0	0
8	AtGOS11	560	746	659	478	430	453	495	917	422	578	522	582	895	906	1024	1152	967	912	1104	826	702	430	422	639	775	703	396
9	AT1G1622	43	35	46	0	3	0.99	0	0	0	0.52	0	0	0	0	0	0	0	0	0	0	140	17	14	3	15	2	6
10	AT1G1623	25	8	13	0	0	0	0	0	0	0	0	0	0	0	0	0	0.56	0.49	0.48	0.45	0	0	0	0	0	0	0
11	AtSYP51	970	1204	1089	981	809	913	1019	1792	993	1204	1105	1119	1566	1588	1755	1986	2340	1901	2157	1656	1047	1072	888	1036	1201	1330	749
12	AtVTI12	51	31	78	4	2	3	0.62	0	2	0	0	0.65	0	0	1	0.99	2	0	0.97	3	80	271	269	1091	61	510	504
13	AtSYP61	691	720	616	712	659	658	713	570	655	718	738	773	671	598	700	936	765	604	696	816	991	624	649	1099	929	689	479
14	AtSFT12	154	188	169	131	104	113	134	150	173	157	155	154	229	215	285	325	309	308	266	226	171	144	103	147	273	211	85
15	AtSYP24	9	3	14	16	10	9	3	0.51	0	2	3	2	5	4	3	13	11	5	4	1	0	0.47	2	31	13	2	4
16	AtNPSN12	342	453	513	796	864	1218	872	989	680	947	736	806	443	408	352	337	176	208	457	850	1203	633	468	1170	1037	883	419
17	AtSYP81	295	309	317	283	220	306	318	218	263	312	268	294	270	282	308	357	371	326	294	305	208	267	221	472	344	369	208
18	AtUSE11	68	70	87	176	161	137	176	112	106	131	161	168	133	142	190	241	67	102	169	133	24	126	62	150	68	157	88
19	AtSYP124	0.91	0.96	3	2	3	2	1	8	7	0.52	1	0.67	3	0.95	2	3	7	8	12	4	0	3	0	29	8	0	1
20	AtSYP52	531	653	572	385	359	367	398	776	367	588	498	512	714	749	754	930	1010	854	933	635	669	494	392	590	450	649	346
21	AtSYP112	15	7	3	42	2	8	53	51	67	12	34	105	12	30	99	80	14	15	9	64	2	12	1	1	31	0	1
22	AtVAMP71	0	0	0	0	0	0	0	0.51	0	0	0	0	0	0	0	0	0	9	0.97	0	0	0	0	0	0	0	0.41
23	AtVAMP72	8	5	15	23	47	34	15	5	2	15	12	18	14	4	3	8	254	167	45	46	22	56	74	109	116	146	91
24	AtVAMP72	85	68	120	171	109	118	125	100	70	113	134	127	166	166	188	185	433	314	299	283	59	99	95	227	71	109	97
25	AtVAMP72	1910	2212	1749	1294	1419	1549	1545	1767	1698	1805	1821	1902	1990	1924	1944	2110	5360	3783	3075	3267	1651	1680	1280	1737	2156	2114	1015
26	AtNPSN11	2379	2512	1496	959	1636	1827	868	631	728	1190	867	654	175	97	28	79	76	50	37	1797	678	1010	928	2032	833	1063	857
27	AtMEMB1	437	491	523	413	401	375	401	520	238	445	390	386	674	632	625	789	675	517	1158	732	326	378	306	511	637	503	254
28	AtGOS12	872	962	862	898	729	949	1050	779	722	959	909	1017	701	566	652	723	441	456	669	1312	525	776	649	1401	628	1072	661
29	AtSYP131	10	2	1	0.55	0.47	1	0	0.51	9	2	0.59	0.67	1	2	9	4	5	22	1	5	14	1	0	0.4	0.56	0	3
30	AtSYP43	753	714	708	609	449	641	680	515	424	726	686	693	532	494	577	518	760	612	567	695	534	328	285	647	579	427	335
31	AtSYP71	2177	2603	1802	1840	1937	1872	2088	1950	1837	1960	1921	1930	1455	1392	1436	1699	1730	1419	1856	2673	2333	1840	1746	2174	2191	3173	1674
32	AtSYP121	790	800	1061	496	575	606	558	1531	521	1161	1272	1155	1083	720	813	869	4125	2375	2070	2581	552	600	554	1026	751	1016	613
33	AtNPSN13	1073	1380	1243	1148	1169	1083	1265	1073	1022	840	804	877	693	638	787	689	726	772	993	1177	878	1087	949	1633	1034	1641	959
34	AtSEC20	330	423	301	401	396	386	388	345	317	358	332	376	293	249	270	347	191	179	307	484	605	339	266	431	865	388	262
35	AtSYP32	1116	1154	799	763	799	889	795	581	635	757	819	716	590	484	472	505	700	548	732	1082	1269	919	841	1442	1360	1377	707
36	AtVAMP72	101	72	123	129	189	126	107	38	73	109	79	84	93	90	97	119	67	72	102	119	118	154	154	356	485	159	125
37	AT3G2501	44	36	48	41	53	50	54	42	36	75	44	45	83	97	107	92	82	65	87	171	22	30	25	62	9	33	37
38	AtVTI13	71	74	111	123	82	92	95	108	63	104	91	95	107	111	128	139	79	87	158	152	111	73	66	130	173	104	78
39	AtSYP72	10	12	10	5	5	11	8	13	0.72	14	13	5	7	3	0.53	4	10	8	0.48	9	0	3	0	0.4	0	0	0.41
40	AtSYP122	628	616	1299	29	4	12	43	495	44	176	170	371	3480	2064	1216	2147	17860	7305	12917	1134	2	19	7	4	38	1	14
41	AtVAMP72	794	1010	694	615	592	569	615	763	617	808	730	754	821	881	853	1059	1515	1339	1076	1028	724	673	653	846	962	859	563
42	AtUSE12	182	212	194	159	128	174	141	175	116	173	160	146	179	171	190	228	232	195	257	258	208	135	114	218	134	149	126
43	AtBS14a	511	619	568	701	732	676	675	776	650	704	653	736	878	891	920	1255	1078	1097	1220	1023	1096	720	623	764	1079	1322	510
44	AtSYP73	10	4	15	8	8	13	5	24	3	13	9	4	25	27	39	33	41	62	65	79	7	3	12	2	22	0	7
45	AtSYP42	169	131	198	192	85	129	173	128	112	165	163	180	185	186	190	250	290	256	267	218	113	84	96	282	72	86	100
46	AtSYP123	160	202	113	75	1	8	48	22	59	25	27	19	11	7	0.51	1	9	13	6	78	5	3	0	4	18	2	2
47	AtBS14b	684	795	673	506	417	468	449	514	402	442	413	480	327	284	349	364	339	364	210	370	476	469	364	564	1083	659	286
48	AtSFT11	203	226	232	285	243	225	240	193	233	198	204	202	219	219	248	304	220	213	220	205	123	338	221	400	608	586	207
49	AtVAMP72	514	585	871	1096	1127	866	947	1189	877	892	766	842	2035	1985	1631	2262	1090	1268	3483	1308	1673	1248	1319	2405	1682	2509	1401
50	AtSYP23	838	945	1112	1148	1148	920	1122	2252	992	1125	877	919	2249	2316	2240	2519	1469	1638	2450	1634	877	859	823	1270	1348	1554	944
51	AtVAMP71	1835	2179	1827	714	673	734	1078	4752	2120	2132	2054	2148	5545	5030	5494	6451	4623	3647	9008	1843	620	680	651	746	1070	835	570
52	AtSYP31	488	494	391	171	160	194	153	366	156	288	210	239	494	436	414	448	662	467	432	471	290	169	136	256	129	232	152
53	AtSNAP29	5	8	10	3	9	8	1	20	0.78	3	2	2	18	16	9	21	34	37	53	13	0	28	12	45	20	42	20
54	AtSYP132	1808	2034	1154	1222	1135	1459	1403	1131	1107	1399	1258	1138	1021	904	811	858	843	713	1028	1625	1071	1014	934	1525	1233	1199	817
55	AtVAMP71	576	702	634	1019	1537	1673	1411	1877	1741	1976	2142	2007	1693	1805	1565	1847	968	927	1255	1448	1873	1461	1170	1519	2576	2044	823
56	AtSYP21	693	593	669	354	184	262	499	729	575	366	424	539	369	378	445	531	1407	1391	1098	1530	284	201	198	354	224	224	157
57	AtVAMP71	754	840	759	486	569	613	600	894	519	806	751	774	826	841	911	901	798	717	1025	1150	214	568	424	573	995	819	344
58	AtSYP41	192	201	292	327	260	360	326	299	166	379	310	321	484	402	421	507	367	324	530	440	369	227	204	572	128	228	238
59	AtVTI11	1511	1630	1427	1172	1129	1336	1347	1786	1334	1797	1664	1620	1960	1814	1647	2291	2324	2168	2541	2123	1295	1335	1132	1454	1217	1594	989
60	AtVTI14	2	0.98	1	0	0	0	0	0	0	0	0	0	0	0	0	0	0.56	0	0.48	0	0	0	0	0	0	0	0.83
61	AtSYP22	1065	1176	1094	1049	1212	1460	1250	1851	1149	1630	1522	1419	1833	1787	1749	2170	1574	1417	1555	1982	937	1237	1087	1537	1750	1819	893
62	AtMEMB1	119	118	148	107	65	102	105	132	67	112	115	124	117	99	121	179	173	153	167	128	17	63	48	194	116	101	50
63	AtYKT61	1425	1621	1172	926	1188	1153	962	1257	1122	1416	1249	1198	1488	1500	1356	1849	2361	2593	1460	2006	963	1221	923	1249	1004	1309	797
64	AtYKT62	0.46	0	0.4	0.54	0.49	0.51	0	0	2	0	0.59	0	0	1	0	0.52	1	0.52	2	0.46	0	0	0	0	0	0.51	0
65	AtSNAP33	960	1134	1372	990	895	990	1134	917	907	1095	1346	1308	1692	1441	1601	1860	6865	4114	3024	2230	1558	1001	987	1567	1110	1566	988

	AC	AD	AE	AF	AG	AH	AI	AJ	AK	AL	AM	AN	AO	AP	AQ	AR	AS	AT	AU	AV	AW	AX	AY	AZ	BA	BB	BC	BD
1	M7	M8	M9	M10	F.AN	F.AN.ad	F.PT.ad	F.FM.ad	F.CA.ad	F.SP.ad	F.CA.y	F.SP.y	F1	F2	F3	F4	F5	F6-8	F9-11	F.AN.y	F12-14	F15-18	F19+	PED	AX	OV.y6-7	STI	POD.y6-7
2	2028	2451	2713	2151	1258	913	3208	4054	2491	1936	2340	2085	2156	2015	1964	1873	1621	1628	3193	1777	1753	2095	2358	2912	2951	2459	3313	2404
3	281	269	260	213	1304	4399	31	14	160	17	247	67	130	219	488	249	151	108	201	123	154	136	224	206	318	189	11	170
4	4069	4918	5481	4053	126	49	47	113	3097	68	4877	755	1328	1369	1103	1290	1563	1631	2543	828	1956	2885	4735	3156	4387	2675	136	2786
5	0	0	1	1	4381	9770	6	16	1	1	0	4	212	404	801	129	2	11	5	0	3	1	0.39	0	0.84	0	4	0
6	584	683	691	521	1720	2186	1211	1586	699	807	564	661	690	666	791	660	626	574	850	682	569	572	595	665	570	736	1323	521
7	0	0	1	1	4232	9247	1	11	3	3	0	0.57	146	346	603	151	90	26	0.84	2	2	0	0	0	0	3	1	1
8	339	336	469	487	8151	7985	907	984	465	867	479	690	779	766	864	682	642	508	864	655	646	512	479	531	413	552	706	588
9	3	0	0	0	0	0	0	0	0	0	0	0	0	0	0	0	0	0	0	0	0.39	0	0.39	0.51	0	0	0	0
10	0	0	0	0	7	0	0	0	0	0	0	0	1	0	0	0	0	0	0	0.46	0.77	0	0	0	0	0	0	0
11	698	726	869	917	5728	5283	1631	1798	929	1581	960	1116	1296	1186	1176	1006	990	993	1456	1455	1111	902	928	963	659	1081	1644	955
12	625	487	435	273	0	0	0	0.56	0.4	0	307	9	0.44	0	0.46	0.43	5	17	44	2	93	21	68	0.51	3	2	0.48	0.8
13	536	524	519	541	1327	1500	984	1033	663	787	563	555	607	542	642	675	724	570	771	600	634	714	624	673	559	586	902	628
14	92	107	132	127	510	440	235	267	142	296	125	139	195	137	204	168	138	146	267	424	186	149	109	120	99	135	437	107
15	7	5	5	3	0	0	0.52	0	2	0	4	1	2	0.92	0	2	2	1	1	0	3	2	10	5	12	1	0	1
16	493	542	631	693	787	798	793	1310	516	472	554	484	464	508	626	558	595	592	660	298	505	469	636	1063	971	448	410	560
17	268	226	279	291	1601	2096	474	384	261	291	292	265	305	338	430	316	319	276	445	373	327	308	263	307	268	316	417	325
18	96	90	115	133	19	122	128	108	104	166	97	146	125	113	127	127	124	133	218	113	154	155	123	190	144	78	137	119
19	7	1	7	6	11037	15337	10	36	5	8	12	4	392	689	782	46	2	7	7	9	16	18	19	2	3	2	2	5
20	328	379	365	320	2900	1981	826	942	447	761	298	372	670	612	477	356	349	342	502	586	469	385	344	389	270	453	528	429
21	1	0.82	2	2	85	256	15	23	39	61	39	19	35	50	64	42	61	52	138	337	844	441	19	51	3	12	10	42
22	0	0	0	0	0	0	0	0	0	0	0	0	0	0	0	0.44	0	0	0	0	0	0	0	0	0	0	0	0.4
23	105	106	86	80	295	4148	84	85	84	42	54	62	63	128	492	366	238	234	265	155	61	56	64	60	44	147	62	65
24	130	105	118	132	145	50	141	124	116	149	140	120	130	110	107	109	120	107	142	76	93	100	101	121	113	107	132	110
25	923	1090	1131	926	2293	1486	2368	3665	1486	1995	1136	1450	1501	1388	1460	1230	1231	1144	1409	1220	1062	1056	1092	1503	1285	1343	3162	1148
26	901	1065	1113	991	2933	5040	1275	2412	984	289	1264	508	634	791	1346	979	1018	791	775	337	768	764	1094	1354	1841	962	919	949
27	317	273	299	415	2334	2469	699	556	417	506	417	434	589	601	578	533	536	456	681	472	455	445	408	515	400	441	653	658
28	708	743	841	855	490	931	1291	1374	918	714	984	867	689	801	970	962	985	890	1053	740	898	1038	919	933	758	959	1398	981
29	1	0.82	12	7	21980	22433	15	42	10	12	0	2	1020	1434	1092	124	61	132	109	25	5	0.4	0.78	2	1	2	23	2
30	438	261	378	418	1010	1276	835	779	389	516	406	462	365	397	516	473	510	460	504	278	454	485	467	594	503	360	606	456
31	1440	1763	1791	1491	847	659	2840	3999	1452	1902	1552	1458	1302	1426	1556	1334	1322	1242	2245	1529	1441	1514	1606	1861	1453	1860	2129	1492
32	598	699	692	592	1334	121	1402	1128	993	1033	1506	2144	1177	948	833	774	717	696	887	659	995	1275	1483	757	646	839	760	1172
33	937	1003	1072	1070	1748	2050	1416	1716	738	772	939	767	625	691	814	614	639	646	836	549	714	672	850	940	777	911	804	835
34	265	268	353	343	730	1042	626	697	357	475	342	427	390	414	467	432	447	398	583	444	411	451	366	471	376	367	580	402
35	705	764	781	633	531	914	1241	1515	734	639	766	751	677	631	846	749	889	733	987	640	682	785	783	830	769	690	945	677
36	124	100	126	152	14	4	74	91	95	55	128	88	95	86	111	103	89	92	141	57	86	93	106	95	127	110	95	148
37	53	41	50	77	32	101	40	24	24	44	37	28	32	40	48	27	43	36	20	13	38	30	29	41	41	32	21	54
38	114	65	114	184	104	205	115	86	125	116	174	119	111	135	156	178	208	171	248	112	241	171	136	141	125	124	123	213
39	0	0	2	0.76	6601	8998	13	14	3	17	0	3	332	492	547	321	295	101	59	127	36	9	1	5	3	0	11	1
40	6	9	2	4	333	42	180	96	241	1748	91	1086	538	323	224	247	287	277	314	72	216	177	243	89	70	66	83	100
41	496	563	627	558	2797	2501	1192	1454	612	1015	521	517	800	776	736	645	625	578	860	844	592	648	535	682	482	712	1089	570
42	147	105	142	187	1001	1081	217	166	148	188	161	155	191	218	230	174	164	152	283	144	198	196	185	166	125	141	186	165
43	426	492	624	555	795	869	1258	1646	596	998	466	776	768	686	732	629	644	625	1054	791	577	552	533	779	565	541	1247	529
44	7	4	10	6	98	119	10	6	15	2	6	2	33	27	17	23	23	19	151	282	135	28	7	10	27	18	1	22
45	112	84	99	136	357	428	233	131	120	223	146	169	170	156	157	169	164	121	238	148	171	187	126	155	145	127	140	162
46	6	4	22	15	206	10	4	10	79	0.53	16	22	54	41	39	22	18	24	31	62	71	147	69	130	84	6	6	72
47	311	313	503	392	358	797	707	1028	335	382	396	381	332	365	557	488	423	375	561	368	397	430	387	463	435	326	823	369
48	213	247	258	297	219	291	341	432	269	240	270	203	251	219	238	234	245	229	505	296	273	313	266	246	222	285	378	266
49	1518	1553	1809	2538	276	194	1537	1123	1152	917	1837	1096	1013	870	946	1099	1066	1068	1949	1303	1555	1708	1389	1188	895	1125	855	1263
50	860	1076	1472	2532	1617	1733	1711	1400	1092	1278	1484	1506	1062	1010	1203	1353	1365	1184	1410	1368	2056	1432	1208	1291	958	871	2048	1404
51	547	532	483	768	23499	21867	3171	3210	746	4575	477	1384	2631	2218	1805	1019	943	656	906	752	682	499	478	776	340	811	999	595
52	147	152	166	220	465	1010	671	580	264	439	272	263	274	308	422	357	373	322	389	336	281	254	234	207	131	336	620	261
53	25	30	28	14	11	9	5	4	10	6	10	1	15	8	7	5	8	8	23	43	54	41	16	5	8	18	5	5
54	854	873	973	921	529	937	3544	3675	1012	1059	975	1130	996	1240	1500	1228	1157	1041	1184	727	1034	987	1043	1304	1165	915	1256	1107
55	794	819	892	675	518	356	848	2140	646	996	698	733	699	696	688	660	651	579	792	493	561	607	765	1204	963	634	615	540
56	147	157	209	203	7229	3388	1401	913	453	1033	271	856	840	780	712	583	535	685	1205	1130	755	446	341	382	192	417	971	453
57	315	352	402	363	764	639	972	1542	412	840	386	521	474	544	556	448	447	428	622	600	492	427	362	546	379	525	814	472
58	353	234	324	468	140	147	296	246	328	295	349	308	325	301	322	335	370	391	521	259	451	389	414	454	345	292	292	569
59	889	997	1180	1114	4808	3511	2077	2581	1022	2086	911	1152	1606	1443	1479	1299	1215	1142	1793	1152	1128	1188	1044	1343	1031	1057	1657	998
60	1	9	14	13	0	0	0	0	0	0	0	0	0	0	0	0	0.83	0	0.42	0	0.78	0	0.39	0	0	0	0	0
61	882	979	1035	948	2130	2732	1632	2850	947	1227	942	1069	1166	1078	1161	1202	1074	989	1189	938	1081	945	1027	1513	1244	903	1424	941
62	47	38	68	65	136	236	146	117	78	109	110	123	79	103	112	113	112	101	160	113	128	120	82	100	73	69	174	90
63	728	784	878	692	4124	3664	1825	2916	996	1624	675	834	1104	1050	1309	1116	1101	891	1182	1068	791	831	740	1115	870	1115	1572	841
64	0	0	0.41	0.38	2418	2069	1	6	2	1	0.75	1	78	215	367	168	120	36	2	6	19	52	2	0	0	0.38	0	0.4
65	837	1015	1089	922	1250	594	1264	1202	1162	1892	1142	1969	1740	1229	1149	1072	1020	1027	1519	1107	1391	1285	1328	1110	949	1156	1496	1092

	BE	BF	BG	BH	BI	BJ	BK	BL	BM	BN	BO	BP	BQ	BR	BS	BT	BU	BV	BW	BX	BY	BZ	CA	CB
1	SD1	SD3	SD5	SL2	SL4	POD1	POD3	POD5	SL6	SD7	POD7	SL8	SD.d	SD.sn1	SL.sn2	SD.g1	SD.g2	SD.g3	POD.sn1	SD.y1	SD.y2	SD.y3	SD.y4	SD.y5
2	2820	2633	2656	2598	2122	2082	1991	1943	2327	2774	1740	2230	2008	1828	1288	3679	2562	2223	2081	2445	2712	2749	2737	2749
3	257	163	152	145	78	50	50	32	54	57	30	35	0	5	2	1	28	47	6	81	118	152	169	169
4	9210	6587	5600	5170	3108	1963	2156	866	1202	992	336	747	27	31	20	4	622	1262	23	1248	1639	2419	2819	2705
5	1	0	3	1	2	5	11	10	11	6	2	23	0	0	0.95	0	4	5	25	4	4	7	7	4
6	906	904	988	889	727	797	785	877	986	1111	999	992	303	452	452	1351	1007	750	958	867	857	896	851	701
7	0	2	1	2	7	4	2	11	10	5	3	6	18	0	2	0	0.53	0	25	3	1	5	2	6
8	729	749	633	1036	796	1127	1055	887	821	759	835	647	1171	1085	659	983	650	619	1117	534	516	608	540	626
9	0	0	0.85	0	0	0	0	0	0	0	0	0	0	0	0	0	0.53	3	0	0	0	0	0	0
10	0	0	0	0	0	0	0	0	0	0.47	0	0	6	0	0	0	11	6	0	0	0	0	0	0
11	1184	1272	1266	1544	1195	1479	1446	1375	1353	1498	1305	1293	5258	3322	2110	1176	1137	1312	2565	1080	1102	1049	1054	1075
12	1681	305	190	787	156	341	98	13	62	36	1	2	2	1	1	59	71	65	0.6	3	2	0.38	1	0.76
13	850	876	855	931	661	736	789	669	692	986	670	766	942	883	649	1048	1035	694	819	757	678	672	570	544
14	262	301	303	330	246	289	285	225	244	320	160	171	498	388	259	460	192	212	668	198	160	121	139	110
15	6	4	1	1	3	0	0.52	1	1	0.47	0.59	1	0	0	1	0.92	1	6	0	0.4	0	1	1	5
16	506	595	576	780	555	681	716	761	600	619	695	660	302	363	259	527	484	588	321	617	548	472	473	393
17	290	359	416	394	274	309	317	320	352	411	310	324	723	486	332	481	384	309	527	287	314	338	270	296
18	152	143	158	154	135	153	130	153	145	152	134	111	636	463	249	294	156	119	113	140	133	105	86	84
19	1	3	8	13	13	8	7	21	26	20	19	29	10	4	4	6	1	1	40	6	11	14	15	6
20	454	647	564	673	480	595	505	422	507	785	438	510	1270	720	557	544	461	775	1270	420	423	465	509	436
21	445	93	11	209	28	79	31	15	8	32	12	12	10	0	2	0.95	8	56	4	7	4	7	4	13
22	0	0.48	0	0	0	0	0	0	0	0	0	0	3299	2761	1500	0	1	1	6	0	0	0	0	0
23	46	103	76	46	48	42	44	41	54	113	36	46	4	9	14	10	12	7	131	64	61	85	111	129
24	114	142	129	132	109	124	118	145	150	118	154	157	161	160	85	158	141	101	109	129	134	126	102	74
25	1547	1859	1991	2335	2019	2265	2073	2195	2543	2122	2464	2344	2240	2147	1764	3227	1995	1887	3982	1489	1656	1666	1607	1490
26	1442	836	856	1228	911	736	1018	936	879	682	1111	1062	5	26	34	393	2153	1505	91	983	1006	1050	951	773
27	450	534	530	735	507	547	518	542	519	625	509	541	1483	1050	658	613	649	400	420	414	395	483	461	463
28	932	1121	1188	1209	937	947	1161	1130	1121	1436	1107	1210	97	216	199	1173	1118	739	436	1184	1212	1126	947	761
29	3	7	24	23	30	14	31	31	75	57	42	74	6	0	20	1	1	40	99	8	21	21	18	3
30	324	320	303	553	451	642	706	723	560	384	669	518	46	112	164	354	658	544	766	380	422	451	360	338
31	1980	1860	1668	2017	1724	2029	2069	1913	1910	1965	1964	1942	2165	2510	1580	1526	1743	2048	2293	1807	1927	2071	1854	1829
32	404	534	566	640	1426	590	1219	2815	2787	1042	4406	3429	239	220	349	283	793	750	648	770	962	1088	1181	1111
33	944	849	838	1032	806	940	1076	984	923	1016	970	937	1114	1150	783	855	754	1006	1364	859	906	894	844	870
34	381	403	391	469	419	400	444	442	442	474	436	436	496	464	296	402	459	437	446	394	423	378	368	400
35	987	851	983	1092	879	915	829	912	1084	1066	1115	1163	499	634	508	945	1369	990	1014	903	906	889	781	721
36	75	62	92	94	63	80	80	93	79	109	84	89	12	30	24	302	137	66	22	154	140	130	155	146
37	27	38	50	58	32	46	46	37	37	66	34	32	53	30	56	103	38	31	19	52	46	40	22	18
38	91	80	105	97	81	103	97	109	92	106	97	66	8	28	33	51	84	91	103	102	116	140	112	95
39	2	1	14	16	18	10	15	24	29	24	18	32	0	1	12	0	0.53	7	42	1	6	5	9	2
40	19	42	92	390	1438	256	816	1950	1835	225	2475	1438	46	24	823	70	117	354	2266	36	42	93	136	95
41	866	893	857	1027	885	878	830	777	924	990	771	863	2212	1537	985	977	831	953	1702	710	752	782	704	756
42	257	247	236	227	208	180	205	210	229	237	190	170	280	417	258	399	235	166	244	179	173	161	141	129
43	1185	1234	1076	1518	1164	1332	1219	1085	1144	1073	877	821	3149	2729	1592	1680	877	762	1912	658	656	622	580	730
44	15	15	47	21	14	13	7	12	24	37	12	16	452	115	58	16	2	7	21	11	11	14	31	13
45	184	161	205	172	163	173	185	220	195	215	247	189	445	335	283	420	215	196	283	135	143	132	110	120
46	1	1	0.43	4	4	1	4	12	11	5	29	48	121	1	4	0	91	166	13	10	9	6	10	3
47	707	805	830	801	641	460	502	474	586	756	399	446	227	396	369	1496	811	507	653	543	468	376	344	326
48	365	349	371	300	247	234	255	230	260	378	183	223	593	634	421	582	336	231	363	330	290	289	258	326
49	1107	1159	992	1834	1278	1665	1588	1514	1436	1411	1348	1263	3242	4371	1883	3353	2814	1067	415	1060	1043	997	1061	988
50	1342	1355	1194	2125	1310	2021	1845	1337	1300	1011	1331	1180	2965	5601	2683	2940	2355	1616	831	831	811	937	936	872
51	1104	1448	1058	2492	1586	2948	2470	1904	1345	1513	1414	1177	314	252	759	508	1857	2316	4484	839	742	670	737	761
52	477	623	665	615	454	451	536	391	446	668	367	379	353	301	162	254	459	413	198	412	410	335	363	303
53	113	164	231	119	101	67	61	32	65	80	5	20	63	106	57	40	5	6	21	20	13	15	10	3
54	807	842	892	1105	872	947	1022	1070	1049	1034	1214	1232	1313	975	764	1360	1829	1665	1030	961	972	991	914	823
55	654	758	896	907	723	950	1005	876	772	728	877	760	551	750	484	774	768	799	1127	741	704	711	767	641
56	604	877	796	951	886	982	817	896	1063	1229	1010	944	2350	1410	1068	584	552	644	2122	516	527	458	432	423
57	576	714	648	918	659	880	826	699	628	756	618	598	1924	1154	789	634	526	652	1168	530	522	475	498	499
58	164	240	223	354	251	352	363	319	250	324	319	269	432	699	431	528	340	247	310	357	367	346	254	222
59	1567	1688	1655	1893	1486	2016	1662	1601	1673	1773	1420	1418	5493	4666	3155	1700	1493	1709	3451	1123	1193	1131	954	1091
60	0	0	0	0	0	0	0	0	0	0	0	0	0	0	0	0	0	0	0.6	0	0	0	0	0
61	1245	1097	1078	1523	1035	1391	1272	1237	1183	1196	1242	1242	2518	2101	1642	1580	1132	1352	2022	901	916	956	935	936
62	97	108	95	111	80	88	74	104	92	122	108	111	193	275	149	261	145	98	137	99	86	89	80	63
63	1422	1629	1550	1651	1373	1675	1654	1450	1469	1660	1289	1353	2208	1926	1478	1521	1205	1398	3272	1091	1059	1124	1139	1123
64	1	19	20	7	10	2	5	5	24	60	4	20	2	1	4	0	0	1	11	34	22	5	4	1
65	1322	1471	1420	1432	1602	1478	1365	1859	2406	1450	2693	2585	2156	1394	1225	989	1000	1129	2102	975	1185	1362	1536	1408
