# Supplementary material for: Vesicle Transport in Plants: A Revised Phylogeny of SNARE Proteins
Source: Evol Bioinform Online. 2020 Oct 15;16:1176934320956575. doi: 10.1177/1176934320956575 (PMC7573729; doi:10.1177/1176934320956575)
Supplement: 2873cc8b46636_EvoBioRevSupplTable4_xyz466366e8025bf – Supplemental material for Vesicle Transport in Plants: A Revised Phylogeny of SNARE Proteins [file 2873cc8b46636_EvoBioRevSupplTable4_xyz466366e8025bf.pdf]

Supplementary Table 4 Identified protein domains and their consensus amino acid sequence in Arabidopsis Q- and R-SNARES. Protein domains discovered by MEME and identified by UniProt across SNARE genes in *Arabidopsis*. Domains are listed in approximate N to C terminal order. When multiple memes contribute to the same identified domain, the meme names are suffixed with "start, "mid" and "end" as appropriate.

Gene_class	MEME_ID	Uniprot_annotation	Arabidopsis_consensus_aasequence
Qa	28	Coiled_coil_start	LDKLDEDVDSVKSRLKGVQKR
Qa	8	Coiled_coil_mid	REAPGCGPGSSVDRTRTSVVAGLRKKLKDVMKEFQKLRERI
Qa	10	Coiled_coil_end	LQDSNEESKTVHNAKAVKELRAKMDGDVAEVLKKAKMIKGKLEALDK
Qa	6	Coiled_coil_end	EYKETVERRYFTVTGEKADEZTIERLIS
Qa	2	tSNARE_start	IQERDDALKEJEKSLLELKQIFLDMAVEVEDQGR
Qa	9	tSNARE_end	DDIEGNVDNASSAVANGTDQLQKAKRTQR
Qa	7	Transmembrane	IAIJJALILJIIVIJ
Qb	9	Coiled_coil_start	DDIEGNVDNASSAVANGTDQLQKAKRTQR
Qb	21	Coiled_coil_end	LTDKMRDCKRLVKEFDRELKDGEARNSPQVNKQLNDEKQSMIKELNSYVA
Qb	16	Coiled_coil_start	GEEKKDKLSEIKSGIEEAEVLISKMDLEARSLPPNSKSDLLRKLREYKSD
Qb	29	Coiled_coil_end	LNNFKTEVKRITSGBLNASARDELLEAGMADTKTASADQRARLMMSTERL
Qb	11	tSNARE_start	TTKAIKNSKRVVEETEEVGVSILSNLHEQREQLLRAHNKLHDIDDSLGKS
Qb	44	tSNARE_end	LVKEIGRQVATDKC
Qb	7	Transmembrane	IAIJJALILJIIVIJ
Qc	26	Coiled_coil	NRAAAVAMNAEIRRTKARLAEDVPKLQRLAVKKVKGLTKEELDARNDLV
Qc	38	Polar	PETEVSDEPFAKDSTNGSSPKIEDEPRSPNSPQLRRRIVPASSKEQSFDA
Qc	2	tSNARE_start	IQERDDALKEJEKSLLELKQIFLDMAVEVEDQGR
Qc	45	tSNARE_end	QMTLIRAQAGVKNNIRKLNLSIIRSGNNH
Qc	28	tSNARE_end	LDKLDEDVDSVKSRLKGVQKR
Qc	9	tSNARE_end	DDIEGNVDNASSAVANGTDQLQKAKRTQR
Qc	7	Transmembrane	IAIJJALILJIIVIJ
Qbc	11	tSNARE	TTKAIKNSKRVVEETEEVGVSILSNLHEQREQLLRAHNKLHDIDDSLGKS
Qbc	2	tSNARE_start	IQERDDALKEJEKSLLELKQIFLDMAVEVEDQGR
Qbc	28	tSNARE_end	LDKLDEDVDSVKSRLKGVQKR
R	4	Longin_start	QSJIYSFVARGTVILAEFTEFKGNFSSIAAQCLZKLPSSNN
R	1	Longin_mid	FTYNCDGHTFNYLVEBGFTYCVVAVESAGRQIPMAFLERVKEDFNKRYGG
R	5	Longin_end	HTAQANSLNKEFGSVLKEHMZYCSDHPDE
R	46	Longin_start	EFIVFIGRTVARRTPPGQRQSVKHEEC

R	33	Longin_mid	VHAYNRNGLCAVGFMDDHYPV
R	17	Longin_end	MNDVFKKYEKQYVELSRQASKDCDSAY
R	3	vSNARE_start	SKLAKVKAZVSEVKGVMMENIEKVLDRGEKJELLVDKTENL
R	12	vSNARE_end	RSQAQDFRTQGTQMRRKMWFQNMKIKLIV
R	42	vSNARE_end	MFYKQAKKTNSCCT
R	7	Transmembrane_start	IAIJJALILJIIVIJ
R	19	Transmembrane_end	CHGFNC
