# Supplementary material for: Vesicle Transport in Plants: A Revised Phylogeny of SNARE Proteins
Source: Evol Bioinform Online. 2020 Oct 15;16:1176934320956575. doi: 10.1177/1176934320956575 (PMC7573729; doi:10.1177/1176934320956575)
Supplement: 3185449246636_EvoBioRevSupplTable1_xyz466364bc83175 – Supplemental material for Vesicle Transport in Plants: A Revised Phylogeny of SNARE Proteins [file 3185449246636_EvoBioRevSupplTable1_xyz466364bc83175.pdf]

Supplementary Table 1 Locus number and function of Arabidopsis Q-SNAREs.

**Name**		**Locus No.**	**Function**	**Reference**
Qa	AtSYP81	At1g51740	Mediates ER-Golgi traffic in the plant secretory pathway.	Bubeck et al. (2008)
	AtSYP31	At5g05760	Mediates cytokinesis and control ER-Golgi trafficking in the plant secretory pathway.	Rancour et al. (2002); Bubeck et al. (2008); Bassham et al. (2008)
	AtSYP32	At3g24350		
	AtSYP41	At5g26980	Mediates secretory and vesicle transport that regulate auxin homeostasis and disease resistance, and maintains the morphology of the Golgi and TGN.	Bassham et al. (2000); Uemura et al. (2004, 2012); Fujiwara et al. (2014)
	AtSYP42	At4g02195		
	AtSYP43	At3g05710		
	AtSYP21	At5g16830	Mediates vesicle transport from post-Golgi membrane to the tonoplast. SYP22 proteins interact with VTI11, SYP51 and VAMP727 to mediate vesicle traffic to these organelles. SYP22 and VAMP727 mediate the BRI trafficking to PM.	Sanderfoot et al. (1999, 2001); Uemura et al. (2010); Yano et al. (2003); Shirakawa et al. (2009); Fujiwara et al. (2014); Zhang et al (2019)
	AtSYP22	At5g46860		
	AtSYP23	At4g17730	Mediates vesicle-mediated transport.	Ohtomo et al. (2005)
	AtSYP24	At1g32270	Forms SNARE complexes with other proteins driving vesicle traffic.	Sanderfoot et al. (1999); Bassham et al. (2000)
	AtSYP111	At1g08560	Induces membrane fusion events forming the cell plate and the transport of secretory vesicles at the plasma membrane.	Lukowitz et al. (1996); EI-Kasmi et al. (2013); Kim et al. (2014); Zhang et al. (2017)
	AtSYP112	At2g18260	Functionally replaces the cell-cycle-regulated SYP111 proteins.	Sanderfoot et al. (2000); Müller et al. (2003)
	AtSYP121	At3g11820	Mediates secretory vesicle traffic at the plasma membrane including as an important structural element determining the gating of K^+ channel to promote K^+ uptake. Mediates the traffic mediated by SYP121 and SYP122 are associated with lots of cargo proteins. SYP121 plays an vital role in pathogen resistance.	Assaad et al. (2004); Uemura et al. (2004); Sutter et al. (2006); Zhang et al. (2007); Grefen and Blatt (2008); Enami et al. (2009); Reichardt et al. (2011); Grefen et al. (2015); Waghmare et al. (2018)

			SYP121 and SYP122 as negative regulators of the programmed cell death reaction, the salicylic acid, jasmonic acid and ethylene signalling pathways.	
	AtSYP122	At3g52400	General function in secretion including a role in cell wall deposition and in tethering of donor and target membrane.	Kargul et al. (2001); Collins et al. (2003); Assaad et al. (2004); Rehman et al. (2008)
	AtSYP123	At4g03330	Can function with SYP132 to induce tip-focused membrane trafficking for root hair tip growth.	Ichikawa et al. (2014)
	AtSYP124	At1g61290	As pollen-specific syntaxins involved in pollen tube growth.	Kato et al. (2010); Silva et al. (2010); Ul-Rehman et al. (2011)
	AtSYP125	At1g11250		
	AtSYP131	At3g03800	General function in secretion including as a component of multiple forms of defense against bacterial pathogens in plants. SYP131 might be important for proper exocytosis during pollen tube growth. SYP132 is required for auxin-stimulated H^+-ATPase traffic and associated functions at the plasma membrane.	Kalde et al. (2007); Reichardt et al. (2011); Ul-Rehman et al. (2011); Slane et al. (2017); Xia et al. (2019)
	AtSYP132	At5g08080		
Qb	AtSEC20	At3g24315	Mediates retrograde traffic from Golgi to ER.	Sanderfoot, (2007)
	AtMEMB11	At2g36900	Mediates anterograde protein trafficking at the ER-Golgi interface.	Chatre et al. (2005)
	AtMEMB12	At5g50440	Mediates retrograde trafficking from Golgi to ER for protein recycling and balance maintenance.	Uemura et al. (2004); Zhang et al. (2011)
	AtGOS11	At1g15880	Mediates anterograde traffic between ER and Golgi and retrograde traffic within the Golgi apparatus.	Tsui et al. (2000); Bubeck et al. (2008)
	AtGOS12	At2g45200		
	AtVTI11	At5g39510	Mediates post-Golgi membrane trafficking to the tonoplast. VTI11 is needed for auxin to be able to both restrict the growth of root	Zheng et al. (1999); Ebine et al. (2008); Bubeck et al. (2008); Löfke et al. (2015)
	AtVTI12	At1g26670		

			epidermal cells and change the appearance of vacuoles.	
	AtVTI13	At3g29100	Interacts with SYP52 to mediate the vesicle transport.	Barozzi et al. (2019)
	AtVTI14	At5g39630	Function in the endosomes and vesicles, including response to gravitropism.	Kato et al. (2002)
	AtNPSN11	At2g35190	General function in secretion and cytokinesis. NPSN11 may contribute to a SNARE complex driving homotypic fusions of a membrane compartment.	Heese et al. (2001); Zheng et al. (2002); Uemura et al. (2004); Lipka et al. (2007)
	AtNPSN12	At1g48240		
	AtNPSN13	At3g17440		
Qc	AtUSE11	At1g54110	Induces membrane fusion in the retrograde trafficking from Golgi to ER.	Burri et al. (2003); Dilcher et al. (2003)
	AtUSE12	At3g55600		
	AtBS14a	At3g58170	Contributes a single helix to form the SNARE complex driving transport from ER to Golgi.	Parlati et al. (2000)
	AtBS14b	At4g14455		
	AtSFT11	At4g14600	Mediates anterograde traffic between ER and Golgi and retrograde traffic within Golgi. Salt and osmotic stress responses in vacuoles.	Tsui et al. (2000); Bubeck et al. (2008); Vaishali et al. (2015)
	AtSFT12	At1g29060		
	AtSYP51	At1g16240	Induces direct membrane transport from ER to tonoplast and Golgi to mediate vesicle trafficking.	Sanderfoot et al. (2001); Ebine et al. (2008); Barozzi et al. (2019)
	AtSYP52	At1g79590		
	AtSYP61	At1g28490	Forms a complex with SYP41 and YKT61 to drive secretion of cell wall enzymes and vesicle trafficking.	Chen et al. (2005); Roy et al. (2017)
	AtSYP71	At3g09740	Induces membrane fusion and traffic pathways to the ER.	Suwastika et al. (2008); Kanazawa et al. (2015)
	AtSYP72	At3g45280		
	AtSYP73	At3g61450		
Qb+c SNAP 25	AtSNAP29	At5g07880	As key proteins for the docking and fusion of synaptic and other vesicles.	Veit et al. (1996)
	AtSNAP30	At1g13890		
	AtSNAP33	At5g61210	Function in general secretion, pathogen defence, and induces cell plate formation in endocytosis from the plasma membrane during cytokinesis.	Kargul et al. (2001); Collins et al. (2003); Dhonukshe et al. (2006); Reichardt et al. (2007)
