# Supplementary material for: Vesicle Transport in Plants: A Revised Phylogeny of SNARE Proteins
Source: Evol Bioinform Online. 2020 Oct 15;16:1176934320956575. doi: 10.1177/1176934320956575 (PMC7573729; doi:10.1177/1176934320956575)
Supplement: 529d24f646636_EvoBioRevSupplTable6_xyz466362abe5173 – Supplemental material for Vesicle Transport in Plants: A Revised Phylogeny of SNARE Proteins [file 529d24f646636_EvoBioRevSupplTable6_xyz466362abe5173.pdf]

**Supplementary Table 6. List of Abbreviations corresponding to *Arabidopsis* tissue samples used for expression studies.**

| Abbreviation | Full name |
|---|---|
| S.R | Seedling root |
| R.A | Root apex |
| R | Root without apex |
| L.LAM.y | Leaf blade of the young leaf |
| L.PET.y | Petiole of the young leaf |
| L.PET.i1 | Petiole, intermediate 1 |
| L.LAM.i1 | Leaf blade, intermediate 1 |
| S.H | Seedling hypocotyl |
| S.C | Seedling cotyledons |
| L.PET.i2 | Petiole, intermediate 2 |
| L.VN.i2 | Leaf vein, intermediate 2 |
| L.LAM.i2 | Leaf blade, intermediate 2 |
| L.PET.lg | Petiole of the mature leaf |
| L.VN.lg | Vein of the mature leaf |
| L.LAM.lg | Leaf blade of the mature leaf |
| L.lg | Whole mature leaf |
| L.PET.sn | Petiole of the senescent leaf |
| L.VN.sn | Vein of the senescent leaf |
| IN.sn | Senescent internode. |
| IN | Internode |
| M1 | SAM at 7 days after germination |
| M2 | SAM at 8 days after germination |
| M3 | SAM at 9 days after germination |
| M4 | Meristem at 10 days after germination |
| S.M | Seedling meristem |
| M5 | Meristem at 11 days after germination |
| M6 | Meristem at 12 days after germination |
| M7 | Inflorescence meristem at 13 days after germination |
| M8 | Inflorescence meristem at 14 days after germination |
| M9 | Inflorescence meristem at 15 days after germination |
| M10 | Inflorescence meristem at 16 days after germination |
| F.AN | Opened anthers |
| F.AN.ad | Anthers of the mature flower (before opening). |
| F.PT.ad | Petals of the mature flower |
| F.FM.ad | Stamen filaments of the mature flower |
| F.CA.ad | Carpels of the mature flower (before pollination |
| F.SP.ad | Sepals of the mature flower |
| F.CA.y | Carpels of the young flower |
| F.SP.y | Sepals of the young flower |
| F1 | Flower 1 |
| F2 | Flower 2 |
| F3 | Flower 3 |

| | |
|---|---|
| F4 | Flower 4 |
| F5 | Flower 5 |
| F6-8 | Flower 6-8 |
| F9-11 | Flower 9-11 |
| F.AN.y | Anthers of the young flower |
| F12-14 | Flower 12-14 |
| F15-18 | Flower 15-18 |
| F19+ | Flower 19+ |
| PED | Pedicel |
| AX | Axis of the inflorescence |
| OV.y6-7 | Ovules from 6th and 7th flowers |
| STI | Stigmatic tissue |
| POD.y6-7 | Carpel of 6th and 7th flowers |
| SD1 | Seeds 1 |
| SD3 | Seeds 3 |
| SD5 | Seeds 5 |
| SL2 | Silique 2 |
| SL4 | Silique 4 |
| POD1 | Pod of the silique 1 |
| POD3 | Pod of the silique 3 |
| POD5 | Pod of the silique 5 |
| SL6 | Silique 6 |
| SD7 | Seeds 7 |
| POD7 | Pod of the silique 7 |
| SL8 | Silique 8 |
| SD.d | Dry seeds |
| SD.sn1 | Seeds of first yellowing silique. |
| SL.sn2 | Senescent silique 2 |
| SD.g1 | Germinating seeds 1 (first day after soaking) |
| SD.g2 | Germinating seeds 2 (second day after soaking) |
| SD.g3 | Germinating seeds 3 (third day after soaking) |
| POD.sn1 | Pod of the senescent silique 1. |
| SD.y1 | Young seeds 1 |
| SD.y2 | Young seeds 2 |
| SD.y3 | Young seeds 3 |
| SD.y4 | Young seeds 4 |
| SD.y5 | Young seeds 5 |
