# Supplementary material for: Vesicle Transport in Plants: A Revised Phylogeny of SNARE Proteins
Source: Evol Bioinform Online. 2020 Oct 15;16:1176934320956575. doi: 10.1177/1176934320956575 (PMC7573729; doi:10.1177/1176934320956575)
Supplement: ef0a8cf646636_EvoBioRevSupplTable3y_xyz466366d196b73 – Supplemental material for Vesicle Transport in Plants: A Revised Phylogeny of SNARE Proteins [file ef0a8cf646636_EvoBioRevSupplTable3y_xyz466366d196b73.pdf]

>P59277|SYP81_ARATH-ARABIDOPSIS
MSRFRDRTEDFKDSVRNSAVSIGYNESKVASTMASFIIHKPKERSPFTKAAFKTLDSIKELELFMLKHRKDYVDLHRT
TEQEKDSIEQEVAAFIKACKEQIDILINSIRNEEANSKGWLGLPADNFNADSIAHKHGVVLILSEKLHSVTAQFDQLRA
TRFQDIINRAMPRRKPKRVIKEATPINTTLGNSESIEPDEIQAQPRRLQQQLLDDETQALQVELSNLLDGARQTET
KMVEMSALNHLMATHVLQQAQQIEFLYDQAVEATKNVELGNKELSQAIQRNSSSRTFLLLFFFVLTFSVLFLDWYS
>I1IZY9|SYP81_BRADI-BRACHYPODIUM
MTRARDRTEDFKESVRVAALAHGYTEAQLAALMSAFIIRKPNPKSPFTKAAIKTLESIRELEKFIVKHRKDYVDTHRTT
EQERDTIEHEVGVFVKACKEQIDILKNRIHEDEKNRSAKTWLSTRDEGSRLDLIAHQHGVVLILSERLHSVTAQFDRL
RSMRFQEAINRAMPRKKIKKKPEIKPAESSKSNLVLQSDVSRVGDQEVSTAPLRVQEQLLDDETRALQVELGNLLDT
VQETETKMIEMSALNHLMSTHVLQQAQQIQYLYDQAVEATNNVERGNKELSQAIQRNSSSRTFILLFFFVLTFSVLF
LDWYKN
>A8JDH4|SYP81_CHLRE-CHLAMYDOMONAS
MQRDYAKEAGALATPDPAKDAAEAQVAAFVKRVGGYIDRLKEAVAAAQKGKQGLAAAGGGGGEGPQQRANEQ
SVAHMTGVVGARVLILAERLHRSAVAFDRLRAARYQALLDRRGPGMLPVPPAASAATAAVLAAAAATGSAASTSG
RGTAKLNGDGGPRGYGPVASARAAALAVARSGWQHLVGGAGGGSSGGLAGSAAGGKGAGGPGPGPGAADGV
DGGAEAWTAGQAQEVEQENKALLERLTATRNAAFSVEQSVRDVAALNQMFSTAVLAQAETIESIYMAAVEATHN
ITRGNESLTKTVAINRSSSRYILVLLLVATACLLFFDWFNS
>C6TGA0|SYP81_SOYBN-GLYCINE
MGKARDRTEDFKDAVRHTARSLGYDEAKLASVMASFIIHKPPQKSPFSKAALKTLESIGELDQFLLKHRKDYTDLHR
TTEKERDSIEHEVSTFIKTCQEQIDILKNSINQEEETSKGWLGIATTKSNADIIAHKHGVVLILSERLHSVTAQFDQLRA
VRFQDAINKAIPRRKLNRVARNDSAETSKSSDMELREPEELRAEPLRVQQQLLDDETRALQVELTSLLDTVQETETK
MVEMSALNHLMSTHVLHQAQQIEHLYDQAVEATNNVELGNKELSQAIQRNSSSRTFLLLFLFVLTFSILFLDWYS
>K7MMR4|SYP81_SOYBN-GLYCINE
MEFREPEELCSEPLRVQQQLLDDETRALQVELTSLLNTGQETETNMVEMSALNHLMSTHVLHQAQQIEHLYDQA
VEATKNVELGNIELSRAIQRNSSSRTFLLLFLSVLTFSILFLDWYS
>Q7X7I8|SYP81_ORYSJ-ORYZA
LEKFIVKHRKDYVDLHRTTEQERDNIEHEVSVFVKACKEQIDILKNRIHEEKGGSTKTWLGTSDESSRLDLIAHQHGV
VLILSERLHSVTVQFDRLRTMRFQDAINRAMPRKRIQKKRETKAAEPSKPNLVLKSDVSKVEDQEVSTAPLRVQEQL
LDDETRALQVELTNLLDAVQETETKMIEMSALNHLMSTHVLQQAQQIQYLYDQAVEATNNVERGNKELSQAIQR
NSSSRTFLLLFFFVLTFSVLFLDWYSK
>A9S7B6|SYP81_PHYPA-PHYSCOMITRELLA
MASIRDLTQKFREAVRAAAVANNYDEGKLAKITSSLMLHSTPPRSTFYLTAIKIAESIRDLQKFVASHGKDYAEKHRS
TEQDRDSIENEVGLFVKACRDSIEALKNSIGMEEKKAYGSNWLQALGRGSPNTDCIAHQHGVVLILSEQLHAITEDF
DKLRSIRFEETVRKKMHKRRKGIENLPPPENVPAPAAYNDGYSNDWLKEEPRMLESRQQLMDQETEALQQELTE
MMNMTQETERKMIELSALNYLFSTHVLRQAQQIESIYMKAMEATSFMEKGNKELAKTQQRNSSSRLYIILIFTVLTT
TLLFLDWYNG
>B9HHK0|SYP81_POPTR-POPULUS
TRICHOCARPAMAKIRDRTEDFKDAVRHIAISLGYNETRLTAIMASFIIHKPRQRSPFTRAALKTLESIGALEQFMLKH
RKDYVDLHRTTEQERDSIEQEVTAFIKACKEQIDILKNSINDEAANTKGWLGIKADTSTTDTIAHKHGVVLILSEKLHS
VTARFDQLRAIRFQDAINKRIPRRKLNRAANTNTTTVDSSKTNNLEFSEPDDIQPESLRVQQQVLDDETRALQVELT
SLLEAVQETETKMLEMSALNHLMSTHVLQQAQQIELLYEQAVEATKNVELGNKELSQAIQRNSSSRTFLLLFLFVLT
FSILFLDWYS
>B9HT21|SYP81_POPTR-POPULUS
MAKIRDRTEDFKDAVRHTAVSLGYNEAKLAAIMASFIIHKPRQRSPFTKAALKTLESIGALEQFMLKHRKDYVDLHR
TTEQERDSIEQEVTAFIKACKEQIDILKDSINNEEANTKGWLGIKADTSNADTIAHKHGVVLILSEKLHSVTARFDQLR
AIRFQDAINKRIPRRKVNRAANKNTSSVDSSKTNNLDFMEPDEIQPEPLRVQQQLLDDETRALQVELTSLLDAVQET
ETKMVEMSALNHLMSTHVLQQAQQIELLYEQAVEATKNVELGNKELSQAVQRNSSSRTFLLLFLFVLTFSILFLDWY
S
>C5YCL0|SYP81_SORBI-SORGHUM
MSRVRDRTEDFKEAVRVAALSHGYTEAQLAALMSSFIIRKPSPKSAFTNAAIKTLESIRELERFIVKHRRDYVDLHRTT
EQERDNIEHEIGVFVKACKEQIDILKNRIHEEEKNGSGKTWLGTRDESSRLDLIAHQHGVVLILSERLHSVTAQFDRL

RSMRFQDAINRAMPRKKILKRPEIKPAEPSKSNLVLKSDVSKIVDQEVSPAPMRVQEQLLDDETKALQVELTSLLDA
VQETETKMIEMSALNHLMSTHVLQQAQQVQYLYDQAVEATNNVERGNKELSQAIQRNSSSRTFLLLFFFVLTFSVL
FLDWYNN
>F6HNM3|SYP81_VITVI-VITIS
MAKIRDRTEDFKDVVRQTALSLGYNESKMASILSSFIIHKPLQRTSFTKAALKTLESIRTLEQFIMQHRKDYVDMHRT
TEQERDSIEHEVTIFIKACKDQIDILKNSISGEEANSRGWLGIRGDHSNADTIAHKHGVVLILSERLHSVTAQFDQLRA
RRFQDAINRRIPRKKMNRVSSSNATEIPKSINSELREPDEPQPEPLRVQQQLLDDETRALQVELSSLLDAVQETETQ
MVEMSALNHLMSTHILQQAQQIELLYEQAVEATSNVELGNKELSQAIQRNSSSRTFLLLFLFEEPMEMGCELIDLQ
PAVF
>Q9FFK1|SYP31_ARATH-ARABIDOPSIS
MGSTFRDRTVELHSLSQTLKKIGAIPSVHQDEDDPASSKRSSPGSEFNKKASRIGLGIKETSQKITRLAKLAKQSTIFN
DRTVEIQELTVLIRNDITGLNMALSDLQTLQNMELADGNYSQDQVGHYTAVCDDLKTRLMGATKQLQDVLTTRSE
NMKAHENRKQLFSTKNAVDSPPQNNAKSVPEPPPWSSSSNPFGNLQQPLLPPLNTGAPPGSQLRRRSAIENAPSQ
QMEMSLLQQTVPKQENYSQSRAVALHSVESRITELSGIFPQLATMVTQQGELAIRIDDNMDESLVNVEGARSALL
QHLTRISSNRWLMMKIFAVIILFLIVFLFFVA
>I1JI73|SYP31_SOYBN-GLYCINE
MASSYRDRTSEFRLLSETMKKIGGPVQPENPPSTSRGGESSYSRSEFNRKASRIGLGIHETSQKIARLAQLARKSSMF
NDPAVEIQELTVLIKNEITTLNSALSDLQTIQNTDMADGGYSQDTIVHSTAVCDDLKSKLMGATKHLQDVLTARTEN
IKAHENRKQIFSKNASRENPFQHQPKPANEPPPWSNSSNASESLQQESALPSNGAPVGNQLRRRLAVDNTPSQQ
MEMSMVQQVVPRHENYAQSRATALHNVESTITELSGIFSHLATMVAHQGELAIRIDDNMDESLANVEGAHSSLL
RHLNRISSNRWLLIKIFAILILFLTIFIFFVA
>I1M7V7|SYP31_SOYBN-GLYCINE
MASSYRDRTSEFRLLLETLKKIGSPVQPENAPSTSHGESYSRSEFNRKASRIGLGIHETSQKIARLTQLARKSSMFNDP
AVEIQELTVLIKNEITALNSALFDLQTVQNTDMADGGYSQDTIVHSTAVCDDLKSKLMGATKHLQDVLAARTENIK
AHENRKQIFSKNASRENPLQHQPKPTTEPPPWSNSSNASESLHQELALPSNGAPVGNQLRRRLAVDSTPSQQME
MSMVQQVVPRHDNYAQSRATALHNVESTITELSGIFSHLATMVAHQGELAIRIDDNMDESSANVEGAHSSLLRHL
NRISSNRWLLIKIFVILILFLMIFIFFVA
>D7T4S5|SYP31_VITVI-VITIS
MAAVSMGHHGPTMPIFHGLWPARRFFAWSRFLFVIDVYISPPPVTVTDYPLQFPMTSSGVSSYRDRTSEFRSLSG
RMKKIGGMAVANHAEDDPATSRSLASASSRSEFNKKASRIGLGIHEACLKISRLAKLAKKSSMFNDPIMEIQELTALI
KDDITALNIAVSDLQTLQNLEIADGNYSDDRVVHSNTVCDDLKNKLMGATKQLQDVLTTRTENIKAHENRKQIFST
NVSRENPFQQHAKTVTEPPPWSSLSKTSGNLQPSVLSSNGVQVGNQLRRRLAVDNTPSNHMEVSMLQQVVPRQ
ENYTQSRALALQNVESTISELSGIFTHLATMVAQQGELAIRIDDNMDESLANVEGAQSALLKHLNQISSNRWLLLKIF
AILIFFLMIFIFFVV
>F4J6K6|SYP32_ARATH-ARABIDOPSIS
MSARHGQSSYRDRSDEFFKIVETLRRSIAPAPAANNVPYGNNRNDGARREDLINKSEFNKRASHIGLAINQTSQKLS
KLAKRIRMVLRSRTDLFSVAKRTSVFDDPTQEIQELTVVIKQEISALNSALVDLQLFRSSQNDEGNNSRDRDKSTHSA
TVVDDLKYRLMDTTKEFKDVLTMRTENMKVHESRRQLFSSNASKESTNPFVRQRPLAAKAAASESVPLPWANGSS
SSSSQLVPWKPGEGESSPLLQQSQQQQQQQQQQMVPLQDTYMQGRAEALHTVESTIHELSSIFTQLATMVSQQ
GEIAIRIDQNMEDTLANVEGAQSQLARYLNSISSNRWLMMKIFFVLIAFLMIFLFFVA
>I1HCM5|SYP32_BRADI-BRACHYPODIUM
MNHPSRSAPASFRDRTNEFRSAVESARRHVAPSPASSSAASASASGGPLDDSRSAASAHSEFNRRASKIGLGIHQTS
QKLARLAKLAKRTSVFDDPTLEIQELTAVVKKDIGALNNAVMDLQVLCNSQNESGNLSKDTTNHSTTVVDNLKNRL
MSATKEFKEVLTMRTENLKVHENRRQMFSSSAAKDASNPFIRQRPLVPREASDSNANPAPWASDSASTPLFQRKK
TNGDHGASSSSSPAFMQQQQQLAVQQDTYMQSRAEALQNVESTIHELSNIFTQLATMVSQQGELAIRIDENMEE
TVANVEGAQGQLLKYLNSISSNRWLMMKIFFVLMVFLMIFIFFVA
>A8JBG7|SYP32_CHLRE-CHLAMYDOMONAS
MPLPQPASLRDRTPEFLAIAERLQRQPGFAPSTSGAPATNGSGPGSGPSTSASSKGQHSEFARRAADIGHGIHRTS
VKLQKLAQLAKRTSAFDDPAQEIDDLTGMIKQDIQGLNNAIADLQRVSARSKGEDRGNKQVSDHSHTVVDNLRSR
LKDTTATFRDVLTARTDSLKHHRERRQLFTSNTDPEAGLPLLARQRAAAAASSSGALGGAAGSSSAPSAPTPSFLAA

SSPAQLQQQQQMQLLAPQDTYLSSRAEALRNVENTIVELGSIFNKLSELVAEQGELAIRIDENVEDTLSNVNAAQA
QLLKYLNGLQNNKWLVLKVLGVLLVFMVLFVMFIA
>I1JUJ6|SYP32_SOYBN-GLYCINE
MYTKSAQSSFRDRTHEFQSIAERLKKTGSAPNGQSSSSSRSEEQRSAIANQSEFNRRASKIGLGIHQTSQKLAKLAKL
AKRTSVFDDPTMEIQELTGVIKQDITALNSAVVDLQLLCNSRNESGNASTDTTSHSTTVVDDLKTRLMSTTKEFKDV
LTMRTENLKVHENRRQLFSANGSKDSANPFVRQRPLATRSAANTSNAPAPPWATGSSSSQLFPKKQVDGESQPLL
QQQQQQQQEVVPLQDSYMQSRAEALQNVESTIHELSNIFNQLATLVSQQGEIAIRIDENMDDTLANVEGAQGAL
LKYLNSISSNRWLMIKIFFVLIFFLMVFLFFVA
>I1JUJ8|SYP32_SOYBN-GLYCINE
MHVKSAQSSFRDRTQEFHSITERLKKSGSGPNGPSSSTSSRSEEQRSAIANQSEFNRRASKIGYGIHQTSQKLAKLA
KLAKRTSVFDDPTMEIQELTGVIKQDITALNSAVVDLQLVCSSRNETGNVSADTSSHSSTVVDDLKTRLMSTTKEFK
DVLTMRTENMKVHENRRQLFSSSASKDSANPFIRQRPLAARAAASTSSAPALPWANGSPSSSQAFPKKQVDGESQ
PLLQQQQQQQEVVPLQDSYMQSRAEALQNVESTIHELSNIFNQLATLVSQQGEIAIRIDENMDDTLANVEGAQG
ALLKYLNSISSNRWLMIKIFFVLIFFLMVFLFFVA
>I1K911|SYP32_SOYBN-GLYCINE
MHTKSAQSSFRDRTHEFQSIAERLKKTGPAPNGQSSSSSRSEEQRSAIANQSEFNRRASKIGFGIHQTSQKLAKLAKL
AKRTSVFDDPTMEIQELTGVIKQDITALNSAVVDLQLLSNSRNESGNASTDTTSHSTTVVDDLKTRLMSATKEFKDV
LTMRTENLKVHENRRQLFSATASKDSANPFVRQRPLATRSAASTSNAPAAPWATGSSSSQLFPKKQVDGESQPLL
QQQQQQQEVVPLQDSYMQNRAEALQNVESTIHELSNIFNQLATLVSQQGEIAIRIDENMDDTLANVEGAQGALL
KYLNNISSNRWLMIKIFFILIFFLMVFLFFVA
>I1K912|SYP32_SOYBN-GLYCINE
MHAKSAQSSFRDRTQEFHSITERLKKSGSGPNGPSSSTSSTRSEEQRSAIANQSEFNRRASKIGYGIHQTSQKLAKLA
KLAKRTSVFDDPTMEIQELTGVIKQDITALNSAVVDLQFLCNSRNESGNVSADTTSHSSTVVDDLKTRLMSTTKEFK
DVLTMRTENMKVHENRRQLFSSSASKDSANPFIRQRPLAARAAASTSNAPALPWANGSPSSSQAFPKKQVDGES
QPLLQQQQQQQEVVPLQDSYMQSRAEALQNVESTIHELSNIFNQLATLVSQQGEIAIRIDENMDDTLANVEGAQ
GALLKYLNSISSNRWLMIKIFSVLIFFLMVFLFFVA
>Q9LGF8|SYP32_ORYSJ-ORYZA
MNPGRPAPASFRDRTNEFRAAVESAARYASSSAAAAPSSSSGGGVGGPLDVSRGAASAHSEFNRRASKIGLGIHQ
TSQKLARLAKLAKRTSVFDDPTVEIQELTAVIKKDITALNSAVVDLQVLCNSQNESGNLSKDTTNHSTTVVDNLKNRL
MSATKEFKEVLTMRTENLKVHENRRQMFSSSAANNASNPFVRQRPLVTRDGPESSVPPAPWASDSATTPLFQRK
KTNGDHGASSSSSQPFMQQQLVQQDSYMQSRAEALQNVESTIHELSNIFTQLATMVSQQGELAIRIDENMDDTL
ANVEGAQGQLLKYLNSISSNRWLMMKIFFVLMVFLMIFIFFVA
>A9S492|SYP32_PHYPA-PHYSCOMITRELLA
MPIAMGPLPSTLGRDRTSEFHAILDRLRKTQGSTLPYTNGAKCSTTNGHADETARLLPQATGALQSEFNKRASQIGL
SIHQTSQKLSKLAKLAKRTSMFDDPAVEIQELTSVVKQDITALNAAISDLQKLCDSRNDGANQSKQSSEHSATVVDT
LKSRLMNTTKEFKDVLTLRTENLKVHDNRRQLFTASPNKQVNPYARQGPLASAVPSSASSTGASLPPWSNGTGRS
NELFSSRRRPTADGLESSQSQGRLQQQQQQLVPVQDSYMQNRAEALQNVESTIVELSSIFTQLASMVAQHGEIAI
RIDENMDESLSNVEGAQTQLLKYLDSISSNRWLILKIFMVLIAFLLIFVVFVA
>A9SZH2|SYP32_PHYPA-PHYSCOMITRELLA
MPIAMGPLPSTSGRDRTSEFHAIVDRLRKTQGSTLPYANEANGAVTNGHAGETARLLPPTTGSLQSEFNKRASQIG
LSIHQTSQKLSKLAKLAKRTSMFDDPAVEIQELTSVVKQDITALNAAISDLQQLCDSRNDGVNQTKHSSEHSTTVVD
TLKSRLMNTTKEFKDVLTLRTENLKVHDNRRQLFTATANKQVNPYARQGPLASAAQNTASSTSVSLPPWGNGAG
RSNELFSSRRRHTADGPESSQSQARLQQQQLAPVQDSYMQNRAEALQNVESTIVELSTIFTQLATMVAQQGEVAI
RIDENMDESLSNVEGAQNQLLKYLDSISSNRWLILKIFMVLITFLLIFVVFVA
>U7DZJ5|SYP32_POPTR-POPULUS
AKRTSVFDDPTLEIQELTAVIKQDITALNAAVVDLQLLCNSQNESGNISSDTTTHSTTVVDNLKNRLMTATKEFKEVL
TTRTENLKVHENRRQLFSSTASKDSSNPFVRQRPLTSRTAASATQAPPPPWANASVSSSQLVPSKSTDVESQPLLQ
QQQQQMVPLQDSYMHSRAEALHNVESTIHELSNIFTQLATMVSQQGELAIRFVLCYGFHCYK
>C5XMQ4|SYP32_SORBI-SORGHUM
MNPTRPAQASFRDRTNEFRAAVESARRQSSAPAAASSSSTGPLDGLMAATSARSEFNNRASKIGLGIHQTSQKLSR
LAKLAKRTSVFDDPTVEIQELTAVIKKDITALNTAVVDLQAICNSQNESGSLSKDTTNHSTTIVDNLKNRLMSATKEFK

EVLTMRTENLKVHENRRQMFSSSAANDASNPFIRQRPLVARDPSESSVPPAPWASDSASTPLFQRKKTNGDHGAS
SSSSQPFAQQQQLAVQQDSYMQSRAEALQNVESTIHELSNIFTQLATMVSQQGELAIRIDDNMDDTLTNVEGAQ
GQLLKYLNSISSNRWLMMKIFFVLMVFLMIFIFFVA
>D7SUI9|SYP32_VITVI-VITIS
MPVKLQQSSFRDRTPEFLNVAERLKKSFSSTQNGANSASKAEEQRFAVAMQSEFNKRASKIGFGIHQTSQKLSKLA
KLAKRTSVFDDPTMEIQELTAVIKQDITALNSAVVDLQLLSNSRNESGNISSDTTSHSTTVVDDLKNRLMSATKEFKE
VLTMRTENLKVHENRRQLFSTASKDSTNPFVRQRPLATRSAASASASPPPWANGSPSSSQLFPRKQIDGESQPLIQ
QQQQQQQQQQQQLVPLQDSYMQSRAEALQNVESTIHELSSIFNQLATLVSQQGELAIRIDENMDDTLANVEGA
QGALLKYLHSISSNRWLMIKIFFVLIFFLMVFLFFVA
>D7TBS9|SYP32_VITVI-VITIS
MSMKSAQSSYRDRTQEFLNVAERLKKSFSSAAPNAVTSSGAKPDGTRSSLAIQKEFKDRASRIGYGIHQTSQKLAKL
AKLAKRTSVFDDPTMEIQELTAVVKQDITALNAAVVDLQLLCNSQNESGNISSDTTSHSTTVVDDLKNRLMSATKEF
KDVLTMRTENLKVHENRRQLFSSTASKESTNPFVRQRPLAAKSTATASSSPPPWANESSSSSPLFPRKQGNVESQPL
LQQQQQQQQLVPLQDSYMQSRAEALQNVESTIHELSNIFTQLATMVSQQGELAIRIDENMEDTLANVEGAQGQL
VRYLNSISSNRWLMIKIFFVLIVFLMIFLFFVA
>O65359|SYP41_ARATH-ARABIDOPSIS
MATRNRTLLFRKYRNSLRSVRAPLSSSSLTGTRSGGVGPVIEMASTSLLNPNRSYAPISTEDPGTSSKGAITVGLPPA
WVDVSEEISVNIQRARTKMAELGKAHAKALMPSFGDGKEDQHNIESLTQEITFLLKKSEKQLQRLSASGPSEDSNV
RKNVQRSLATDLQLLSMELRKKQSTYLKRLRQQKEDGMDLEMNLSRNRYRPEEDDFGDMLNEHQMSKIKKSEEV
SVEREKEIQQVVESVNDLAQIMKDLSALVIDQGTIVDRIDYNIENVATTVEDGLKQLQKAERTQRHGGMVKCASVL
VILCFIMLLLLILKEIFL
>I1H1H1|SYP41_BRADI-BRACHYPODIUM
MATRNRTPLYRKYRDALRHVRAPAGAPSSSSSSGGGGGGPVIEMASLLRPDRNYAPLSTDDPSAASSRGAVTVGL
PPAWVDVSEEISANMQRAKTKMAELAKAHAKALMPSFGDGRDDQRAIEVLTHEVTDLLKRSEKKLQKLSMKDSS
EDSNIRKNVQRSLATDLQNLSMEFRRKQSSYLKQLRQQKEGQDGVDLEMNINGSKSTFQLEDDEFEDVGFTEVQ
MSKLKKSEAFTRERERIEQVVESVNELAQIMKDLSVLVIDQGTIVDRIDYNIQNVAASVEEGYKQLQKAERTQKKG
GMVMCATVLVILIFIMIILLILKKILF
>A8IK60|SYP41_CHLRE-CHLAMYDOMONAS
MPPFGSTRNLTQQFIRLRNEARRLQHSGGPPQGDKATEKLMSAALGSSSDVEAAGAASSSVSPVWVLQSERIRVE
MNLVKERLVKLKEYHAKALLVTFDGESEAQVHAEALTREIQQSFKRLDAAIRAMAQSTGRNEDAEVRLQVQRQLA
SALFKLSVEFRKEETRFLNKVEQQKGLEAGSVIGLVEADEGTKTGGEPVDPGFTQAQLAMVDISTDLITERDSEIRKIV
EAIAELAQIMRDMSTLVLEQGTMLDRIDHNIAQTSVKVEEGVKQLKAAETTQKRGRMFICIIALIVLIVLMLIIVIIRHI
>I1JQP7|SYP41_SOYBN-GLYCINE
MATRNRTLEFRKHRDAVKSVRAPLSSSASSPVIEMVTTSLLPSNRSSYAPLSTQEHAPSTSRDAFTVGLPPSWVDDS
EEIATNIQRARVRISELTKAHAKALMPSFGDGKEDQRHIETLTQEITSLLRKSEVRLKRLSAAAGSSEDSNVRKNVQRS
HATDLQNLSMDLRRKQSAYLKHLQQQQEGYDGVDLEMNFNGSKFVSHNDEFSDVGFSEEQMTKLKRSEQFSEER
EREIEQVVKSVHELAQIMKDLSVLVIDQGTIVDRIDYNIQSVSTSVEEGLKQLQKAERIQKKGGMVMCASTLVIMCF
VMLVLLILKEILF
>I1LB67|SYP41_SOYBN-GLYCINE
MATRNRTLLFRKHRDALKSVRIPSFSSAPSTASGAGGGPVIELATTSFLNSNRSYTPISTDDPGNSSRGPNAITVGLPP
VWVDLSEEIAANVQRARTKMGELAKAHSKALMPSFGDGKEDQRAIETLTHEITDLIKKSEKRLRRLSATGPSEDSNV
RKNVQRSLATDLQNLSVELRKKQSTYLKRLRQQKEGQDGVDLEMLNGSKSKYEDDDLDNMVFNEHQMAKLKKSE
AFTIEREKEIQQVVESVNELAQIMKDLSVLVIDQGTIVDRIDYNIQNVATTVEDGLKQLQKAERTQKKGGMVMCAT
VLLIMCFVMLVLLIIKEIIL
>C6TBP9|SYP41_SOYBN-GLYCINE
MATRNRTIEFRKHRDAVKSVRAPLSSSASASTGPVIEMVTTSLLPPNRSSSYALLSTQEPAPSTSRDAFTVGLPPSWV
DDSEEIATNIQRARVKISELTKAHAKALMPSFGDGKEDQRHIETLTQEITSLLRKSEVRLRRLSAAAGSSEDSNVRKN
VQRSLATDLQNLSMDLRRKQSAYLKRLQQQQEGYDGIDLEMSFTGSKFGSQNDEFSDVGFSEEQMTKLKRSEQFS
EEREREIEQVVKSVHELAQIMKDLSVLVIDQGTIVDRIDYNIQSVSTSVEEGLKQLQKAERTQKKGGMVMCATTLVI
MCFVMLVLLILKEILF
>I1NJ45|SYP41_SOYBN-GLYCINE

MATRNRTLLFRKHRDALKSVRVPSISSPPFTASGAGGGPVIELATTSFLNPNRSYAPLSTEDPGNSSRGPNAITVGLP
PAWVDLSEEISANVQRARTKMAELAKAHSKALMPSFGDGKEDQRAIETLTHEITDLIKKSEKRLRRLSATGPSEDSN
VRKNVQRSLATDLQNLSVELRKKQSTYLKRLRQQKEGQDGVDLEMLNGSKSRYEDDDLDNMVFNEHQMAKLKN
SEAFTVEREKEIQQVVESVNELAQIMKDLSVLVIDQGTIVDRIDYNIQNVATTVEDGLKQLQKAERTQKKGGMVM
CATVLLIMCFVMLVLLIIKEIIL
>Q0DF40|SYP41_ORYSJ-ORYZA
MATRNRTPLYRKYRDALRHVRAPAGAPSSSSSGGGGGGGGGGPVIEMASLLRSNRPYAPLSTDDPSAASSRSAVT
VGLPPAWVDVSEEISANMQRARTKMAELAKAHAKALMPSFGDGRDDQRAIEILTHEVTDLLKRSEKRLQKLSMKD
SSEDSNVRKNVQVMMGSYLYLLNSYRTHKGFLF
>Q5VRL4|SYP41_ORYSJ-ORYZA
MATRNRTPLYRKYRDALRHVRAPAGAPSSSSSGGGGGGGGGGPVIEMASLLRSNRPYAPLSTDDPSAASSRSAVT
VGLPPAWVDVSEEISANMQRARTKMAELAKAHAKALMPSFGDGRDDQRAIEILTHEVTDLLKRSEKRLQKLSMKD
SSEDSNVRKNVQRSLATDLQSLSMEFRKKQSTYLKQLRQQKEGQDGVDLEMNMNGSKSTFELGDDEFEDVGFTE
VQMSKLKKSEAFTREREREIEQVVESVNELAQIMKDLSVLVIDQGTIIDRIDYNIQNVAASVEEGYKQLQKAERTQKK
GGMVMCATTLVILIFIMIVLLILKKILF
>A9TGD0|SYP41_PHYPA-PHYSCOMITRELLA
MATRSQTALFRKYREALRSVRPYAASSSRHGGAIELGYNAIGSEDLDARHLSAGSSTMNLPPGWVDISDQVSADM
QRARTKMAELAKAHSRALMPSFDETSKEEHTIELLSQEITKLLKKCEQKLQQLSRPSGPSEQDANIRKNVQRSLATDL
QTLFMDFRKQQKGYLNKLQRQQEGQAVDDGIGLRKQPKTSEDDDFSESFTNQHLQQLRQNEALSIEREKEISQIVE
SVQDLAQIMKDLSVLVIDQGTIVDRIDYNIMNVASSVEQGVKELVKAEETQKRGGMVTCILVLIVLCAAMLIIFVLKK
IIGL
>A9RUD3|SYP41_PHYPA-PHYSCOMITRELLA
MATRNQTALFRKYRQALRSVRPFAGASSRHGGAIELVEAPLLKGGPRGYNAVVGEDLDADRLSAGSSTLNLPPGW
VDISDQVSADMQRARSKMAELAKAHSRALMPSFDDFSKEEHTIELLSQQITKLLKKCEQKLQQLSRPSGPSEQDASI
RKNVQRSLATDLQTLSMDFRKQQKGYLNRLQRQQEGQAVDDGIGLRKQPKLSEDDDFSQSLSNQHLQQLRQNE
ALSIEREKEISQIVESVNDLAQIMKDLSVLVIDQGTIVDRIDYNITNVAASVEQGVKELVKAEETQKRGGMVTCILVLI
VLCAAMLIIYVLKKIIGL
>B9GSE4|SYP41_POPTR-POPULUS
MATRNRTVVYKKHRDEVKSVRAPLSSSLPGSSGPVIEMVSASFLRSQHSSYTPLSTEDPGPSTSSGDAFTIGLPLAW
VDDSEEISLNIQRIRTKMGELVKAHAKALMPTFGDGKEDERVIEGLTREITGLLRNSGTRLKKISASESFEDSNVRKNV
QRALATELQNLSMDLRRKQSMYLKRLQQQKEGHDGVDLEMSLNANKFRSEDDGFSDVGFNEGQMLKLKKSEQF
TVDRESEIKQVTESVHELAQIMKDLSVLVIDQGTIVDRIDYNIHNVASTVEEGFKQLQKAERNQNKGGMVMCATV
LVIMCFIMLTLLILKESLL
>B9I719|SYP41_POPTR-POPULUS
MATRNRTLIFRKYRDALKSVRVPTSSSPSTSSVGGVGGSGGGPVIELASTSLLNPNRKYAPLSTEDPGNSSKGAFTVG
LPPAWVDVSEEIAANVQRARMKMVELAKAHAKALMPSFGDGKEDQRTIEGLTQEITGLLRKSEKQLKRLAAAGPS
EDSNVRKNVQRSLATDLQNLSMELRKKQSTYLKRLRQQKEGQDGDDLEMNLNGGRSIIDDDNLDDMVFNEHQ
MAKLKKSEAFTVEREREIQQVVESVNELAQIMKDLSVLVIDQGTIVDRIDYNIQNVATTVEEGLKQLQKAERTQKRG
GMVMCATVLVIMCAVMLILLILKTILF
>C5XL52|SYP41_SORBI-SORGHUM
MATRNRTPLYRKYRDALRHVRAPSGAPSSSSGAGGGAGGGGPVIEMASLLRSDRPYAPLSTEDPSGSSRGAVTVG
LPPAWVDVSEEISANMQRARTKMAELAKAHAKALMPSFGDGRDDQRAIEVLTHEVTDLLKRSEKRLQKLSMKDS
SEDSNVRKNVQRSLATDLQNLSMEFRKKQSSYLKQLRQQKEGQDGVDLEMNINGTKSTFEDDEFDDVGFTEVQM
SKLKKSEAFTREREREIEQVVESVNELAQIMKDLSVLVIDQGTIIDRIDYNIQNVAASVEEGYKQLQKAERTQKKGGM
VMCATVLVILIFIMIVLLILKKIIF
>D7U2E4|SYP41_VITVI-VITIS
MATMNRTVVFRKHRDAVNSVRAPAGVSSGGAVIEMASTSLFHSNRSSYTPLSTEDPGPSSKDAFTVGLPPAWVD
VSEEVAANIQRSRVKMAELVKAQAKALMPSFEDGKEDQRKIESLTQEITDLLKRSEKRLQKLSARGPSEDSNVRKNV
QRSLATDLQNLSLELRKRQSTYLKRLRQQKEGHDGVDLEMNLNENKFRLDDDEFGDMGFNEHQMAKLKKSEKFT
AEREKEIRQVVESVNELAQIMKDLSVLVIDQGTIVDRIDYNIQSVSASVEEGFKQLEKAERTQRKGGMVTCATILVIM
CFIMLVLLILKEIFL

>D7UA00|SYP41_VITVI-VITIS
MASRNRTLLYRKYRDALKSVRVPVSSSLSSSTPSTSSGGGPVIELVSTSLLNPNRSYVPLSTEDPGNSSKGALTVGLPP
AWVDVSEEISANVQRARTKMAELVKAHAKALMPSFGDGKEDQHRIESLTQEITDLLKKSEKRLQKLSSTGPSEDSN
VRKNVQRSLATDLQNLSMELRKKQSSYLKRLRQQKEGQDGVDLEMNLNGNKSLREDDEFSDLGFNEHQMTKLKK
NEAFTAEREREIQQVVESVNELAQIMKDLSVLVIDQGTIVDRIDYNIQNVAASVEEGFKQLQKAERTQKKGGMVM
CATVLVIMCFVMLVLLILKTILL
>D7UCH1|SYP41_VITVI-VITIS
MSVIDLIERVDAICRRYEKYDLDNHNEALGSQYDVVEAGIEASLQKLEAATTEEDRAFDVAMKADVQQTKAYLLEE
VSELQKLTLERVNVLSKEEFVAQNDLISMLKERIEAISDGIARGAKQTGGWATSASHEGIKIGSTSDKRFGSNYCQQT
EESIRFRQGNEMQKMKQD
>Q9SWH4|SYP42_ARATH-ARABIDOPSIS
MATRNRTTVYRKHRDACKSARAPLSLSASDSFGGPVIEMVSGSFSRSNHSSYAPLNSYDPGPSSSDAFTIGMPPAW
VDDSEEITFNIQKVRDKMNELAKAHSKALMPTFGDNKGIHREVEMLTHEITDLLRKSEKRLQMLSTRGPSEESNLRK
NVQRSLATDLQNLSMELRRKQSTYLKRLQQQKEGQDEVDLEFNVNGKMSRLDEEDELGGMGFDEHQTIKLKEGQ
HVSAEREREIQQVLGSVNDLAQIMKDLSALVIDQGTIVDRIDYNVQNVSTSVEEGYKQLQKAERTQREGAMVKCA
TILLVLCLIMIVLLILKNILF
>Q9SUJ1|SYP43_ARATH-ARABIDOPSIS
MATRNRTLLFRKYRNSLRSVRAPMGSSSSSTLTEHNSLTGAKSGLGPVIEMASTSLLNPNRSYAPVSTEDPGNSSRG
TITVGLPPDWVDVSEEISVYIQRARTKMAELGKAHAKALMPSFGDGKEDQHQIETLTQEVTFLLKKSEKQLQRLSAA
GPSEDSNVRKNVQRSLATDLQNLSMELRKKQSTYLKRLRLQKEDGADLEMNLNGSRYKAEDDDFDDMVFSEHQ
MSKIKKSEEISIEREKEIQQVVESVSELAQIMKDLSALVIDQGTIVDRIDYNIQNVASTVDDGLKQLQKAERTQRQGG
MVMCASVLVILCFIMLVLLILKEILL
>Q39233|SYP21_ARATH-ARABIDOPSIS
MSFQDLEAGTRSPAPNRFTGGRQQRPSSRGDPSQEVAAGIFRISTAVNSFFRLVNSIGTPKDTLELRDKLQKTRLQI
SELVKNTSAKLKEASEADLHGSASQIKKIADAKLAKDFQSVLKEFQKAQRLAAEREITYTPVVTKEIPTSYNAPELDTES
LRISQQQALLLQSRRQEVVFLDNEITFNEAIIEEREQGIREIEDQIRDVNGMFKDLALMVNHQGNIVDDISSNLDNS
HAATTQATVQLRKAAKTQRSNSSLTCLLILIFGIVLLIVIIVVLV
>I1HE07|SYP21_BRADI-BRACHYPODIUM
MSFQDLEAGNGPRGAPRRNGRSAAGAGAGASQAVASGVFQINTAVATFQRLVNTLGTPKDTPDLRDRIHKTRAH
ITQLVKDTSDKLRQASEADHRVEVSATKKIADAKLAKDFQAVLKEFQKAQRLSAEREAAYAPFITQAGLPQSYNSTD
MNNGADKLAEQRTQLLESRRQELVFLDNEIVFNEAIIEERDQGIQEIQHQITEVNEIFKDLAVLVHDQGAMIDDIDS
HIDNSVAATAQAKGQLSKAAKTQKSNSSLICLLMVIFGVVLLIVIIVLAA
>I1J4T0|SYP21_SOYBN-GLYCINE
MSFQDIEAGRSFASRRNLINGKQDPTQAVASGIFQINTAVSTFQRLVNTLGTPKDTPELRDKLHKTRLHIGQLVKDT
SAKLKQASEIDHNVEVNASKKIADAKLAKDFQAVLKEFQKAQRLSAERETAYTPFVPQALLPSSYTASEVDIGSDKTP
EQRALLVESRRQEVLFLDNEISFNEAIIEEREQGIQEIQSQIGEVNEIFKDLAVLVHEQGAMIDDIGSNIEHSHEATAQ
AKSQLAKASKTQRSNSSLTCLLLVIFGIVLLIVIIVLAA
>I1L4Z2|SYP21_SOYBN-GLYCINE
MSFQDIEAGRSFAARRNLINGKQDPTQAVASGIFQINTAVSTFQRLVNTLGTPKDTPELRDKLHKTRLHIGQLVKDT
SAKLKQASEIDHNVEVNTSKKIADAKLAKDFQAVLKEFQKAQRLSAERETAYTPFVPQGALPSSYTASEVDISSDKTP
EQRALLVESRRQEVLFLDNEIAFNEAIIDERDQGIQEIQSQIGEVNEIFKDLAVLVHEQGAMIDDIGSNIEHSHAATV
QAKSQLAKASKTQRSNSSLTCLLLVIFGIVLLIVIIVLAA
>C6TA84|SYP21_SOYBN-GLYCINE
MSFQDIEAGRPFSSRRGLMNGKQDPTQAVAAGIFQINTAVSTFQRLVNTLGTPKDTPDLREKLHKTRLHIGQLVKD
TSAKLKQASDIDHHAEVNASKKIADAKLAKDFQAVLKEFQKAQRLAAERETAYTPFVPQAVLPSSYTASEVDVGSDK
SPEQRALLVESRRQEVLFLDNEIAFNEAIIEEREQGIQEIQQQIGEVNEIFKDLAVLVHEQGAMIDDIGSNIENSHAAT
AQAKSQLAKASKTQRSNSSLTCLLLVIFGIVLLIVIIVLAA
>Q9S7H0|SYP21_ORYSJ-ORYZA
MSFQDLEAGNARGLPRRGGGGRAGAAAAGAGASQAVASGVFQINTAVSTFQRLVNTLGTPKDTPDLRERIHKTR
QHITQLVKDTSEKLKQASEADHRVEVSASKKIADAKLAKDFQAVLKEFQKAQRLAVEREAAYAPFISQAGLPQSYNS

SEVNNGADKLAEQRTALLESRRQELVFLDNEIVFNEAVIEERDQGIQEIQHQITEVNEIFKDLAVLVHDQGQMIDDI
DTHIENAVIATTQAKGQLSKAAKTQKSNSSLICLLLVIFGVVLLIVIIVLAA
>A9SF79|SYP21_PHYPA-PHYSCOMITRELLA
MSFRDIEAGGLPSGPMLQDSTQALASIVFQINTAVSSFKRLVNSLVTDIDTPVLREKLQPTPVVEFVAWAQNAASYS
KLVKETGSKWKVASGHDHNRLVYDNKKLRDAKFAKDFQAVLVEFQNAQRIAQERESCNIQMTCIQCQRKTWNRK
RSMLHKDRKSLPSTLKEAGFP
>A9SGJ6|SYP21_PHYPA-PHYSCOMITRELLA
MSFEDFESGSNAVAIRQDRGDVVASGIFRINTSVSTYKRLVNTLGTPTDNHALREKIHATEQKISKLVEETMARLKEE
NETDHFSSVSTNKKVRDAKLARDFQAVLLEFQSAQKAAQSRQRKYAPVLPPAASLPQLDSGRGGRDEEAPLLQQS
QQLIVQANESEVMFNNVVIEEREQGIQEIQQQIGEVSEIFKDLAQIVSNQGYIIDDIEANIESSASSTVQANMHLTRA
AKSQKSSEYWKCVILAIVGTVLFAFLVIMFA
>A9S044|SYP21_PHYPA-PHYSCOMITRELLA
MSFEDFETGRNAGSRRQDHSDTVAAGIFRINTNVATYKRLVNTLGTPRDDHGLREKIHATEQKISKLVEETVAKLKE
ENETDHLFSASTSKKIRDAKLAKDFQAVLLEFQGAQKAAQSRQRKYGPVLPPASSSVELDSIRGKSDDNVPLLRQSQ
QLNIQANESEVIFNTVVIEEREQGIQEIQQQIGEVSEIFKDLAQIVSNQGHLIDDIEANIESAASSTVQANIHLTRAAKS
HKSSEYWKCVILAIIGTVLFAFLIILFA
>A9S759|SYP21_PHYPA-PHYSCOMITRELLA
MSFLDIEAGGMPPGRRQQDSTQALASGVFQINTAVSSFKRLVSSLGTAKDTPALRDKLHKTRQHIGQLAKETGAKL
KTASEHDHNRPVHGNKKLSDAKLAKDFQAVLVEFQNAQKIAQEREKLYAPFVPEAALPTSQYSGEMKSAPEENQD
QRAFYAAQRSQDFIQLENETVFNEAVIEEREQGIREIHQQIGEVNEIFKDLAVLVHDQGYMIEDIDANVQGAEAATE
QANRQLAKAAKSQKSGTTMTCLILVIVAMAVLVLLLFLTR
>B9MU58|SYP21_POPTR-POPULUS
MSFQDLEAGRPLASSRRELINGKQDATQAVASGIFQINTAVSTFQRLVNTLGTPKDTPELREKLHKTRLHIGQLVKD
TSARLKQASETDHYAGVSQSKKIADAKLAKDFQAVLKEFQKAQRLAAERETAYTPFVPQAVLPSSYTASEIDLSFDKS
PEQRAILVESRRQEVLLLDNEIAFNEAVIEEREQGIHEIQQQIGEVNEIFKDLAVLVHEQGTMIDDIGSHIENSQAATA
QGKSHLVKAAKTQRSNSSLACLLMVIFGIVILIVIVVLAA
>B9GZI3|SYP21_POPTR-POPULUS
MSFQDLEAGRPLASSRRELINGKQDATQAVASGIFQINTAVSTFQRLVNTLGTPKDTPELREKLHRTRLHIGQLVKD
TSARLKQASETDHYAGVSQSKKIADAKLAKDFQAVLKEFQKAQRLAAERETAYAPFVPQAVLPSSYTASEVNVSSEK
SPEQRALLVESRRQEVLLLDNEIVFNEAIIEEREQGIHEIQQQIGEVNEIFKDLAVLVHEQGAMIDDIGSHIESAQAAT
SQGTSQLVKAAKTQRSNSSLACLLMVIFGIVILIVIVVLAA
>C5XFU8|SYP21_SORBI-SORGHUM
MSFQDLEAGHVRGAPLGGGRRNGRGPAGGAGASQAVASGVFQINTAVATFQRLVNTLGTPKDTPDLRDRIHKT
RQHITQLVKDTSDKLKQASEADHRVEVSATKKIADAKLAKDFQAVLKEFQKAQRLAVEREAAYAPFISQAGLPQSY
NSSEVNNGADKLAEQRTQLLESRRQELVFLDNEIVFNEAIIEERDQGIQEIQHQITEVNEIFKDLAVLVHDQGAMID
DIDSHIENAVVATSQAKGQLSKAAKTQKSNSSLICLLLVIFGVVLLIVIIVLAA
>C5Z6Z6|SYP21_SORBI-SORGHUM
MSFQDVCHDLEAGHPLPPRPPPPPPPRAVVAHCVFQINTKVSELRRLAHELGAAGLGGNGDARVVRERIRRARAD
VTRLARNTARRLADPAAAAAVGPNLAADFQAALREFQWVQDRIIEADRQETAAAAAAARLAPPLMFPPPSPPSY
GSPIRSSQLNNANTNATAGAADQQCNIQMQQQQQLVESRRTQELALLDNEIAFNEALVEERGREICKIQQEIAEIN
EIFVELAKLVRDQQWNIDVVESNVEKAAMETSKAEEKLSKAALTHETNSSMNCLLITVFGLVMLIFALVFVA
>A5BYQ4|SYP21_VITVI-VITIS
MSFQDLESGRPLAQSRRDYINAKQDPTQAVASGIFQINTAVSTFQRLVNTLGTPKDTSELREKLHKTRLHIGQLVKD
TSAKLKQASETDHHAEVSASKKIADAKLAKDFQAVLKEFQKAQRLAAERETAYTPFVPQSVLPSSYTASEIDVGPDKS
PEQRALLVESRRQEVLLLDNEIVFNEAIIEEREQGIQEIQHQIGEVNEIFKDLAVLVHEQGVMIDDIGSNIDGAQAAT
AQAKSQLAKASKTQRSNSSLTCLLLVIFGIILLIVIIVIAA
>D7T7N3|SYP21_VITVI-VITIS
MSFEDLEWGRTRPGQSPLALATKRREEDDSSQAVAAGVFRINTAVSAFYRLVNSLGTPKDTLELREKLHKTRLHIGQ
LVKDTSAKLKQASENDQHTEVSASKRIADAKLAKDFQAVLKEFQKAQRLAVERETAYTPFVPKEVLPSSYDARELEIS
SGKNLEQQAVLLESRRQEVVLLDNEITFNEAIIEEREQGIQEIQQQIGEVNEIFKDLAVLVHGQGTMIDDISSNIEKSH
AATGQASTQLEKASKLQKSNSSLSCLLLVIFGVILIIVVIVVVA

>P93654|SYP22_ARATH-ARABIDOPSIS
MSFQDLESGRGRSTRKFNGGRQDSTQAVASGIFQINTGVSTFQRLVNTLGTPKDTPELREKLHKTRLHIGQLVKDT
SAKLKEASETDHQSGVNPSKKIADAKLARDFQAVLKEFQKAQQTAAERETTYTPFVPQSALPSSYTAGEVDKVPEQ
RAQLQESKRQELVLLDNEIAFNEAVIEEREQGIQEIHQQIGEVNEIFKDLAVLVNDQGVMIDDIGTHIDNSRAATSQ
GKSQLVQAAKTQKSNSSLTCLLLVIFGIVLLIVIIVLAA
>A8MS65|SYP23_ARATH-ARABIDOPSIS
MSFQDLEAGRGRSLASSRNINGGGSRQDTTQDVASGIFQINTSVSTFHRLVNTLGTPKDTPELREKLHKTRLYIGQL
VKDTSAKLKEASETDHQRGVNQKKKIVDAKLAKDFQAVLKEFQKAQRLAAERETVYAPLVHKPSLPSSYTSSEIDVN
GDKHPEQRALLVESKRQELVLLDNEIAFNEAVIEEREQGIQEIQQQIGEVHEIFKDLAVLVHDQGNMIDDIGTHIDN
SYAATAQGKSHLVRHQRHKDQILLCLISPSS
>Q9C615|SYP24_ARATH-ARABIDOPSIS
MVRSNDVKFQVYDAELTHFDLESNNNLQYSLSLNLSIRNSKSSIGIHYDRFEATVYYMNQRLGAVPMPLFYLGSKN
TMLLRALFEGQTLVLLKGNERKKFEDDQKTGVYRIDVKLSINFRVMVLHLVTWPMKPVVRCHLKIPLALGSSNSTG
GHKKMLLIGQLVKDTSANLREASETDHRRDVAQSKKIADAKLAKDFEAALKEFQKAQHITVERETSYIPFDPKGSFSS
SEVDIGYDRSQEQRVLMESRRQEIVLLDNEISLNEARIEAREQGIQEVKHQISEVMEMFKDLAVMVDHQGTIDDID
EKIDNLRSAAAQGKSHLVKASNTQGSNSSLLFSCSLLLFFFLSGDLCRCVCVGSENPRLNPTRRKAWCEEEDEEQRK
KQQKKKTMSEKRRREEKKVNKPNGFVFCVLGHK
>Q42374|SY111_ARATH-ARABIDOPSIS
MNDLMTKSFMSYVDLKKAAMKDMEAGPDFDLEMASTKADKMDENLSSFLEEAEYVKAEMGLISETLARIEQYHE
ESKGVHKAESVKSLRNKISNEIVSGLRKAKSIKSKLEEMDKANKEIKRLSGTPVYRSRTAVTNGLRKKLKEVMMEFQG
LRQKMMSEYKETVERRYFTVTGEHANDEMIEKIITDNAGGEEFLTRAIQEHGKGKVLETVVEIQDRYDAAKEIEKSLL
ELHQVFLDMAVMVESQGEQMDEIEHHVINASHYVADGANELKTAKSHQRNSRKWMCIGIIVLLLIILIVVIPIITSFS
SS
>I1GNK5|SYP111_BRADI-BRACHYPODIUM
MNDLMTKSFMSYVDLKKAAMKDLEAGGGDETELTEAGCAAGGVTDERLKGFFKEAEVVKEEMAAIRDALARLHA
INEEGKSLHQPEALRAMRGRVNADIVAVLRRARDIRVRLEAMDRANAAQRRLSAGCSEGTPLDRTRTSVTAGLRK
KLKDLMLDFQALRQRMMSEYKETVERRYYTLTGEVPEDEVIERIISEGRGEEIMGAAVAEHGKGAVLAALHEIQDR
HDAAREVERSLLELHQVFLDMAVVVESQGEQIDDIERHVVNARDYVHTGNKELGKAREHQRSSRKCLCIGLILLLLLI
LIVVVPVVTSFKTS
>I1JD32|SYP111_SOYBN-GLYCINE
MNDLMTKSFTSYVELKKVAMKEDVDLEAGGPGDRKVELSSSTTHLDTDMGLFLEEAEKVKAEMGSLRDILGRLQQ
ANEESKSLHKPEAHKALRSRINAEILAVLKKARAIRTQLEEMDRANAANRRLSALKDGTPAIYRTRIAVTNGLRKKLKE
LMMDFQDLRQRMMTEYKDTVCRRYFTVTGEHPDEDVIEKIIANGNEEEVLAKAIEEHGRGKVLDTVLEIQDRHDA
AKEVEKSLLELHQVFLDMAVMVEAQGEKMDDIEHHVFHASHYVKDGTKSLQSAKEYQKRSRKWMCIGIILLLILILV
IVIPIVTSLSSS
>I1MNS3|SYP111_SOYBN-GLYCINE
MNDLMTKSFTSYVDLKKAAMKEDVDLEAGVVVSSATPRNVELTSSTTHLDTDMGLFLEEAEKVKAEMGSLRDILG
RLQQANEESKSLHKPEALKVLRARINADIVAVLKKARAIRAQLEEMDRANAANRRLSGLKEGTPAIYRTRIAVTNGL
RKKLKELMMEFQGLRQRMMSEYKDTVGRRYFTVTGEHPDEEVIEKIIANGNEEEVLGKAIQEHGRGKVLETVVEIQ
DRHDAAKEVEKSLLELHQVFLDMAVMVEAQGEKMDDIEHHVLHASHYVKDGTKNLQSAKEYQKKSRKWMCIGII
LLLILILFIVIPVVTSLSSS
>Q84R43|SYP111_ORYSJ-ORYZA
MNDLMTKSFMSYVDLKKAAMKDLEAGGDGVELPEVGVTDERLKGFFQETEAVEEEMAAIRDALARLNAANEEG
KSLHQPDALRALRGRVNADIIAVLRRARDIRARLEAMDRANAAQRRLSAGCREGTPLDRTRTALTAALRKKLKDLM
LDFQALRQRIMSEYKDTVERRYYTLTGEVPEEEVIERIISEGRSEELLCAAVAEHGKGAVLATVHEIQDRHDAAREVE
RSLLELHQVFLDMAVVVESQGEQLDDIERHVNSATTYVQGGNKELRKAREHQRSSRKWLCIGIIILLLLVLLVIVPIAT
SFKRS
>B9I5T9|SYP111_POPTR-POPULUS
MNDLMTKSFMSYVDLKKEAMKDLEAGPDPVVEMANASNTMESNLGMFLEEAENVKKEMGSIREILDQLQEANE
ESKTLHKPEALKSLRNKINTDIVTVQKKARSIKSQLEEMDRANAANRRLSGYKEGTPIYRTRIAVTNGLRKKLKELMM
DFQGLRQKMMTEYKDTVGRRYFTVTGEYPDEEVIDKIISDGSGGEEFLKRAIQEHGKGKVLETVVEIQDRHDAAKEI

EKSLLELHQVFLDMAVMVEAQGEQMDDIEHHVLNASHYVKDGTKELKGAKGYQKSSRKWMCIGIILLLIIILVIVIPI
ATSFSHS
>F6H3C6|SYP111_VITVI-VITIS
MNDLMTKSFISYVDLKKEAMKDLEAGPEYDLQMSGTQMDRNLGLFLEEAEKVKQEMGLIREILGRLHEANEESKS
LHKPEALKSLRNRINADIIGVQKKARAIKSQLEEMDRANAANMRLSGYKEGTPVYRTRAAVTNGLRKKLKELMMD
FQGLRQRMMTEYKETVGRRYFTVTGEYPDEEVIEKIISNGEGGEEFLGRAIQEHGRGKVLETVVEIQDRHDAAKEIE
KSLLELHQVFLDMAIMVEAQGEQMDDIEHHVMNAAQYVKDGTKNLKTAKDYQRSSRKCMCLGVILLLILILIVVIPI
ATSFSNS
>Q9ZPV9|SYP112_ARATH-ARABIDOPSIS
MNDLMTKSFLSYVELKKQARTDMESDRDLEKGEDFNFDFSPADEENLSGFFQEIETIKTLIEEITHLLLDLQNLNEETK
STHSTKILRGLRDRMESNIVTISRKANTVKTLIETLEKRNVANRTSFKEGSCVDRTRTSITNGVRKKLRDTMSEFHRLR
ERIFADYREDLKRKYFLATGEEPSNEDMEKMISGSGSCSDLVKTFEVKPEMDLKTKERHEAVNDIKRSLNRLHQVFL
DMAVLVETQGDRIDDIEANVANAGSFVSGGTNSLYYANQMKKKTKSWVLWVSILGVLILLVCVISMLASR
>I1J8T7|SYP112_SOYBN-GLYCINE
MNDLMTKSFLSYVELKKQARKDLEDDDLDLEAGKLNPTEDRNLSQFFQEVEAIKVEMEEISNLLFDLQQLNEEAKC
THGAKVLRGLRDRMESDMVAVLRKARTIKAMLEVLDQSNIANRSLSESYKEGSPIDRTRMSVTNGLRVKLRDMM
NDFLSLRDKILSDHKEDLKRRYYTATGEVPTEEVMEKMVSGSLKVEFLAGKTDADLGTQVRHEAVMDIQRSLNKLH
QVFLDMAILVETQGEKLDNIEDNVVNAGNFIHGGTNSLYYADQMKKKNRKWLCWVFAVGLIILLVCIIAMLSS
>K7KQY1|SYP112_SOYBN-GLYCINE
MNDLMTKSFQSYAELKKQAEKDNLEDSHDIEAGKLNNPTDYHNLSQFFQEVEAIKVEMEEVATLLFDLLQLHEETK
CTDSAKVLRGLRDRMVSDMVTLFCKARIINSRFEVLDQSNITNHTLSESCKEGIQIDDQNVCHKSFCWPGSCGQC
W
>I1LHU2|SYP112_SOYBN-GLYCINE
MNDLMTKSFLSYVELKKQARKDLEDDDLDIEAGKLNPTEDRSLSQFFQEVEAIKFEMEEITNLLFDLQQLNEETKCT
HGAKVLRGLRDRMESDMVAVLRKALIIKAMLEVLDQSNIDNRSLSESYKEGSPIDRTRMSVTNGLRVKLRDMMND
FQSLRDKILSEHKEDLKRRYYTTTGEVPTEEVMEKMVSGSLKVEFLAGKTDADLGTQVRHEALMDIQRSLNKLHQV
FLDMAILVETQGEKLDNIEDNVVNAGNFIHGGTNSLYNANQMKKKNRKWLCWVFAVGLIILLVCIVAMLSS
>F6GWV5|SYP112_VITVI-VITIS
MNDLMTKSFLSYVELKKQAEMDLEAEAEADIEIGKLKPKDEENLSQFFEEVAAIKTVMEEITNLVLDLHNLNEETKST
HSAEVLRGLRDRMDSNALTILRKAKVVKARLEAIDKSNVRNRRISEAYREGSPVDRTRMSVTNGLRSKLRDMMND
FHSLRERILWDHRETLKRRYYNATGSEASEEVVEKMMTGSVQIEAFEGKTGGDLVNRERNEALREIQRSLDKLRQV
LLDMAVLVGSQGEKMDDIEENVAIAGNFISGGTNSLVYAKQMKKGKKWVYWVWAVGLIILLVCFISMLTS
>Q9ZSD4|SYP121_ARATH-ARABIDOPSIS
MNDLFSSSFSRFRSGEPSPRRDVAGGGDGVQMANPAGSTGGVNLDKFFEDVESVKEELKELDRLNETLSSCHEQS
KTLHNAKAVKDLRSKMDGDVGVALKKAKMIKVKLEALDRANAANRSLPGCGPGSSSDRTRTSVLNGLRKKLMDS
MDSFNRLRELISSEYRETVQRRYFTVTGENPDERTLDRLISTGESERFLQKAIQEQGRGRVLDTINEIQERHDAVKDIE
KNLRELHQVFLDMAVLVEHQGAQLDDIESHVGRASSFIRGGTDQLQTARVYQKNTRKWTCIAIIILIIIITVVVLAVLK
PWNNSSGGGGGGGGGGTTGGSQPNSGTPPNPPQARRLLR
>I1GMH7|SYP121_BRADI-BRACHYPODIUM
MNNLFSSSWKRAGDGDLESGGVEMSAPPGAAAGASLDKFFEDVESIKDDLRDLDRIQRSLHDGNESGKSLHDASA
VRALRARMDADAAAAIKKAKVVKLRLESLDRANAANRSVPGCGPGTSTDRTRTSVVAGLRKKLRDSMESFSALRA
RVSSEYRDTVARRYFTVTGAQPDEATLDTLAETGEGERFLQRAIAEQGQGRGEVMGVVAEIQERHGAVADLERSL
LELHQVFNDMAVLVAAQGEQLDDIEGHVGRARSFVDRGREQLQVARKHQKSSRKWKFIAIGILLVIILVIVIPIVLKN
TKNSSSSNNSNQPQQ
>I1JGG8|SYP121_SOYBN-GLYCINE
MNDLFSGSFSRTNDQVSPDHHHVIEMAATASPTAEGSVNLEKFFQEVEQVKEELKELERLHENLRGSHEKSKILHS
AKAVKELRLRMDSDVTLALKNAKLVKVRLEALDRSNQTSQSLPGSGPGSSSDRTRTSVVSGLRKKLKDSMDSFNSLR
QKISSEYRETVQRRYYTVTGENPDDKTIDLLISTGESETFLQKAIQQQGRASVMDTIQEIQERHDTVKEIERNLNELH
QVFLDMAVLVQSQGEQLDDIESHVARANSYVRGGVQQLHVARKHQKNTRKWTCIAIILLIIIILIIVLPIVLRN
>I1JGG9|SYP121_SOYBN-GLYCINE

MNDLFSGSFSRFRSDQSSPDRHHDIEMGATASSGGRGGEVNLDKFFEDVEGVKEELKELEGLAQSLRSSHEQSKTL
HNAKAVRDLRARMDGDVSAALKKAKLIKLKLEALERSNAANRNMPGCGPGSSSDRTRTSVVNGLKKKLKDAMESF
NEIRQLVSSEYRETVQRRYFTVTGENPDDKTLDLLISTGESETFLQKAIQEQGRGRILDTITEIQERHDAVKEIEKNLKE
LHQVFLDMTVLVQHQGEQLDDIESHVARAHSFVRTGAEQLQTARKHQKNTRKWTCYCIILLLVIILFVVLFTVKPW
ENSSSGGNGGGQPAPAQTPPSPSPPVNA
>I1JQ97|SYP121_SOYBN-GLYCINE
MNDLLSGLFSKGKQEEHVIEITEGGGIMELEKFLEEVESVKEDLKELERLHLSLDATNQNGKALHSPKGVRELRSRM
DLDVALSLTKAKHVKGRLAALHRANQATLSLPDCGPGSYSDRTRTALVGALTKNLRQSMASFNKLREQISYEYRDTV
QRRYYAVTGENPDQETIDLLISTGESETFLQKAIQQQGRASVMDTIQEIRERHGTMKEIERSLHELHQVFMDMAVL
IQHQGEHLDDIESHVELANSFVSKGVQHLQVVRNHQKNTRNFTCFAVLLFIIVLVIILPIVFRN
>I1L9L2|SYP121_SOYBN-GLYCINE
MNDLFSGSFSRFRSDQSSPDRHHDIEMGAAAGGPRGGEVNLDKFFEDVEGVNEELKELEGLAQSLRSSHEQSKTL
HNAKAVRDLRSGMDGDVSAALKKAKLIKLKLEALERSNAANRNLPGCGPGSSSDRTRTSVVNGLKKKLKDSMESF
NEIRGLVSSEYRETVQRRYFTVTGENPDDKTLDLLISTGESETFLQKAIQEQGRGRILDTINEIQERHDAVKEIEKNLKE
LHQVFLDMTVLVQHQGEQLDDIESHVARAHSFVRTGAEQLQTARKYQKNTRKWTCYCIILLLVIIFFVVLFTVRPWK
NNSSGGGNNGNQPAPAQTPPPPVNA
>I1L9L3|SYP121_SOYBN-GLYCINE
MNDLFSGSFSRNNDQVLPDHHHHVIEMAAASSPTAEGSANLEKFFQEVEQVKEDLKELERLHENLRGNHEKSKTL
HSAKAVKELRSRMDADVALALKKAKLVKVRLETLDRSNQVSRNLPGLEPGSSSDRTRTSVVSGLRKKLKDSMDSFN
SLRQQISSEYRETVQRRYYTVTGENPDDKTIDLLISTGESETFLQKAIEQQGRASVMDTIQEIQERHDTVKEIERNLNE
LHQVFLDMAVLVQSQGEQLDDIESHVARANSYVRGGVQQLHVARKHQKNTRKWTCIAIILLIIIILIIVLPIVLRK
>I1NAV2|SYP121_SOYBN-GLYCINE
MNDLLSGLFSKGKQQQQQQQRHQHVIEITQGGGMDLDKFFQEVESVKEDLKELERLHLSLRATNQHGKALHSP
KGVRELRSRMDLDVALSLTKAKLVKGRLAALHRTNQATLSLPGCGPGSYSDRTRTALVGALTKNLRQSMESFNKLR
EQISYEYRDTVQRRYYAVTGENPDQETIDLLISTGESETFLQKAIQQQGRATIMDTIQEIQERHDTMKEIERNLHELH
QVFMDMAVLIQHQGEHLDNIESHMELANSFVSIGVQHLQVVRSHQKNTRNCTCFAILLFIIVLVIVLPIVFRN
>Q6F3B4|SYP121_ORYSJ-ORYZA
MNNLFSSSWKRTGGGGGGDGDIESGGGVEMAPPPGAAAGASLDRFFEDVESIKDELRDLERIQRSLHDANEGGK
SLHDAAAVRALRARMDADVAAAIKKAKVVKLRLESLDRANAANRSVPGCGPGSSTDRTRTSVVAGLRKKLRDSME
SFSSLRARISSEYRETVARRYYTVTGEQPDEATLDNLAETGEGERFLQRAIAEQGRGEVLGVVAEIQERHGAVAELER
SLLELHQVFNDMAVLVAAQGEQLDDIETHVGRARSFVDRGREQLVVARKHQKSTRKWTCIAIIILLVLILVVVLPIVL
KFVNNNKSSSSSPAPATPSPPPPTA
>B9H9L1|SYP121_POPTR-POPULUS
MNDLFSGSFSRFHSEEASPDHHVIQMSEAQSTGGGVNLDKFFEDVESIKDELKELERLNENMRSSHEQSKTLHNAR
AVKDMRSKMDADVALALKRAKLIKVRLEALDRSNAANRSLPGCGPGSSSDRTRTSVVSGLRKKLKDLMESFNGLR
QKITTEYRETVERRYFTVTGENPDEKTLDLLISTGESETFLQKAIQQQGRGRILDTINEIQERHDAVKDLENNLKELHQ
VFMDMAVLVEHQGEQLDDIESHVQRANSYVRGGTQQLQTARKLQRNSRKWTCYAIIILLIIILVVLLSVRPWEKKK
>B9IIJ7|SYP121_POPTR-POPULUS
MNDLFSSSFSRFSSEEAPPAHHVIQMSEAPSTGGVNLDKFFEDVESIKDELKELERLNGNLQSAHEQSKTLHNSRAV
KDLRSKMDADVALALKKAKLIKVRLEALDRSNAANRTLPGCGAGSSSDRTRTSVVNGLRKKLKDLMDGFNGLRQKI
STEYRETVQRRYFTVTGENPDEKTIDLLISTGESETFLQKAIQQQGRGRILDTINEIQERHDAVKDLENNLKELHQVFL
DMAVLVEHQGEQLDDIESNMQRANSFVRGGTQQLQTARKLQKNTRKWTCYAIIILLIIILVVLLILRPWK
>C5WY64|SYP121_SORBI-SORGHUM
MNSLFSSSWKRGGGDDGDIESGSVEMSAPPGAAAGASLDRFFEDVESIKDELRDLERIQRSLHDGNEAGKSLHDA
SAVRDLRARMDADVSAAIKKAKVVKLRLESLDRANAANRSVPGCGPGSSTDRTRTSVVAGLRKKLRDSMESFSSLR
SRVASEYRDTVARRYFTVTGTQPDEATLDALAESGEGERFLQRAIAEQGRGQVLGVVAEIQERHGAVAELERSLLEL
QQVFNDMAVLVAAQGEQLDDIEGNVGRARSFVDRGREQLQVARKHQKSTRKWTCIAILILLVIILVIVLPIVLQNTK
KN
>F6I3K6|SYP121_VITVI-VITIS
MNDLFSGSFSRFRSEEPPPSVEMTSSTAGVNLDKFFEDVESIKEELREMESLQQKLHDAHEQSKTLHNANSVKELRS
RMDSHVSLALKKAKLIKLRLEALDRSNAANRSLPGCGPGSSSDRTRTSVVNGLRKKLRDSMDAFTSIRNQISSEYRET

VQRRYFTVTGENPDEKTVDLLISTGESETFLQKAIQEQGRGRVLDTISEIRERHESVKELERNLKELHQVFLDMAVLV
QAQGEQLDDIESQVARANSFVTGGTQQLQTARKHQISSRKWTCYGIIILIVIILLIVLFTVRPWENNGSSSSGSTSSTS
PPPPPPPPPTQA
>Q9SVC2|SYP122_ARATH-ARABIDOPSIS
MNDLLSGSFKTSVADGSSPPHSHNIEMSKAKVSGGSCHGGNNLDTFFLDVEVVNEDLKELDRLCHNLRSSNEQSK
TLHNANAVKELKKKMDADVTAALKTARRLKGNLEALDRANEVNRSLPESGPGSSSDRQRTSVVNGLRKKLKDEME
KFSRVRETITNEYKETVGRMCFTVTGEYPDEATLERLISTGESETFLQKAIQEQGRGRILDTINEIQERHDAVKDIEKSL
NELHQVFLDMAVLVEHQGAQLDDIEGNVKRANSLVRSGADRLVKARFYQKNTRKWTCFAILLLLIIVVLIVVFTVKP
WESNGGGGGGAPRQATPVQAQPPPPPAVNRRLLR
>Q9ZQZ8|SYP123_ARATH-ARABIDOPSIS
MNDLISSSFKRYTDLNHQVQLDDIESQNVSLDSGNLDEFFGYVESVKEDMKAVDEIHKRLQDANEESKTVHDSKAV
KKLRARMDSSVTEVLKRVKMIKTKLVALEKSNAAQRKVAGCGPGSSADRTRTSVVSGLGKKLKDMMDDFQRLRTK
MATEYKETVERRYFTVTGQKADEETVEKLISSGESERFLQKAIQEQGRGQVMDTLSEIQERHDTVKEIERSLLELHQV
FLDMAALVEAQGNMLNDIESNVSKASSFVMRGTDQLHGAKVLQRNNRKWACIATILAIVVVIVILFPILFNTLLRP
>I1GXQ0|SYP123_BRADI-BRACHYPODIUM
MNDLFSTSSFKKYADASPQGAGGGDMEAGGESVANLDMFFEEVEAVKEDMRGFETLYKRLQSTNEETKTAHEAR
AIKSLRSRMDGDVEQVLKRAKAVKAKLEALDKDNANSRKAPGCGPGSSTDRTRTSVVAGLGKKLKDIMDDFQGLR
TRMAAEYKETVARRYYTVTGEHAEESTIESLISSGESESFMQKAIQDQGRGQVMDTISEIQERHDAVKEIERSLMDL
HQVFLDMAALVEAQGHQLNDIESHVAHASSFVRRGTVELETAHEIQKDSRKWMCFAVLGGIAIVIVLVTPVLINLHI
LTLR
>I1KQP4|SYP123_SOYBN-GLYCINE
MNDLFSNSFKKYSNLKQQAHLDDVEAGKETVNLDKFFEDVENVKEEMRTVEKLHRKLQEANEESKVVHNAKTMK
ELRARMDKDVEQVLKRVKVIKGKLEALERSNAANRNIPGCGPGSSADRTRTSVVSGLGKKLKDMMDDFQGLRTR
MQMEYKETVERRYFTITGEKPDKETIENLIWSGESESFLQRAIQEQGKGQIMDTISEIQERHDAVKEIEKNLIELHQV
FLDMAALVESQGQQLNNIESHVAHASSFVRRGTDQLQDAREYQKSSRKWTCYAIILGLVLLLVLLFPILINLLPHLLR
>I1M2C2|SYP123_SOYBN-GLYCINE
MNDLFSSSFKKYSDLKEQSHIDDVEAGKESVNLDKFFDEVENVKEDMRLVEKLYRKLQESNEESKIVHNAKTMKDL
RARMDQDVQQVLKRVKLIKGKLESLERSNAANRNIPGCGPGSSADRTRTSVVSGLGKKLKDLMDDFQGLRARMQ
NEYKETVERRYFTITGEKADEDTIENLISSGESESFLQRAIQEQGRGQIMDTISEIQERHDAVKEIEKNLIELHQVFLD
MAALVESQGQQLNNIESHVARASSFVRRGTEQLQDAREYQKSSRKWTCYAILLGIVLVVVLLFPLLTSLLPHLLY
>Q6H8D0|SYP123_ORYSJ-ORYZA
MNDLFSSGSFKKYADLKNQAALDDMESGGGGGGGGEGANLEQFFEEVEGVKGEMRGLEALHGRLQASHEGSKT
AHDARAVRSLRARMDADVEQVLRRARAVKGRLQALDRANAASRKLPGRGPGSSTDRTRSSIVSGLGTKLKDLMD
DFQGLRSRMAEEYKETVARRYYTVTGEKAEESTVEALISSGESETFLQKAIQEQGRGQVLDTISEIQERHDAVKEIER
GLLDLHQVFLDMAALVEAQGHQLNDIESHVARANSFVRRGAVELETAREYQRSSRKWACIAILAGVVLVVIIVLPIIV
NLHLLTIR
>Q69X85|SYP123_ORYSJ-ORYZA
MNDLFSSSSFKKYADASPASGVGGSDMEAGGEGVVNLDRFFEDVEGVKEDMKGLEALYKRLQSTNEETKTAHDA
RAVKALRSRMDGDVEQVLRRAKAVKGKLEALDRDNATSRKVPGCGPGSSTDRTRTSVVAGLGKKLKDIMDDFQG
LRTRMAAEYKETVARRYYTVTGEKAEDSTIDSLIESGESESFLQKAIQEQGRGQVMDTISEIQERHDAVKDIERSLLDL
HQVFLDMAALVEAQGHQLNDIESHVAHASSFVRRGTVELEVAREHQKSSRKWACVAVLAGIILIAVLILPVLINLRIL
TLR
>A9SFE5|SYP123_PHYPA-PHYSCOMITRELLA
MNDLLSQTFSARKAGGAYDDLESGPSTQMADLGGGDQKLDGFFADVEKIKADMDKIKQLLLKLQEANEESKGVH
RAPAMKALRERMDTDIAQVSKLARGLKGKLEALERGNAASRRVKGCEEGTPTDRTRMTITINQRKKLKDLMGEFQ
VLRERMMNEYRETIERRYYTVTGQQADEDTIETIIETGQSESFLQKAIQEQGRGHVMETIREIQERHDSVKEIEKNLL
ELHQIFMDMAVLVESQGEQLNNIEAQVNRSASYVERGTTHLRVAKSHQRSKRKWTCIAIILLVILLLIIILPILKSNKVI
>A9SA01|SYP123_PHYPA-PHYSCOMITRELLA
MNNLLSNSFGKAMNYVDLKKDIRRGDIELGEASIGDGEVDMSQFFDEVGVIKSEMEKIKHCLEKVKDANEESRTVH
KAQAMKALRSRMDADIAQVTKIAKSIKFKLEELDRANAANRRVRGCEEGTPTDRTRSSITNTLRKKLKDLMGEFQIL
RQKMMEEYKETVERRYYTVTGQHADDETIENIIETGNSETFLQKAIQEQGRGQVLETIKEIQERHDAVKDIERNLIEL

HQIFMDMATLVETQGEQLNDIESQVNKAASFVERGTTQLKIAKNHQRNTRKWMCMGIALVIILILLILLPLLHTVGA
I
>A9SNS7|SYP123_PHYPA-PHYSCOMITRELLA
MNNFLNNPLGKAKNYVDLKKDARRGDIEMGDTAGSGGESEVDMTQFFEEVGVIKSEMDKIKQLLEKVKAANEES
RIVHKAQAMKALRSRMDADIAQVTKIAKSIKLKLEDLDRANAANRRVRGCEEGTPTDRTRTSITGTLRKKLKDLMG
EFQTLRQKMMEEYKETVERRYYTVTGEHADDDTIEHIIETGNSETFLQKAIQEQGRGQVLETIKEIQERHDAVKDIER
NLIELHSIFMDMATLVEAQGEQLNNIESHVNKASSFIDRGTQQLKIAKDHQRNTRKWMCIGIALVIILILIILLPILKSV
GAFDRSAPPPK
>A9SA02|SYP123_PHYPA-PHYSCOMITRELLA
MNDLLSRSFGRDGSNYVDLKKDSRHGDIELGNKASTGPEIDMTKFFDEVAVIKGEMEKIKQLLSKVQDAHDESRTV
TKAQGMKALRARMETDIRQVTKIAKAIKFKLEELDKANVENRRVIGCEEGAPTDRTRTSITSTLRKKLKDLMGEFQIL
RQNMNDEYKESVERRYYTVTGEHADEETIDTIIETGNSETFLQKAIQEQGRGHVLETIKEIQERHDAVKDIERSLLELH
QIFMDMATLVDAQGEQLNDIEQQVNKASSFIQRGTQQLQVAKNTQRSSRKWCLIAIILLIVLILVLAIPLLRSFGLL
>A9U4D6|SYP123_PHYPA-PHYSCOMITRELLA
MNDLFSRSFGREGSNYVDLKKDSRKGDVELGEKSVAGGPEIDMGPFFAEVDKIKSEMEKIKQLLAKVQGAHEESRT
VSKAQAMKDLRVRMDNDIKQVTKIAKTIKSKLEELDKANVENRKVRGCEEGTPTDRTRVSITSTLRKKLKDLMGEF
QILRQNMNEEYKETVERRYYTVTGKHADDETIETIIETGNSETFLQKAIQEQGRGQVLETIKEIQERHDAVKDIERNLL
ELHQIFMDMATLVDAQGEQLNDIEQQMGKASSFIARGAQNLQVAKNNQRSSRKWCCIAIILLIILLLILVIPILHSSGL
I
>A9TRV1|SYP123_PHYPA-PHYSCOMITRELLA
MSDLFKGIWKGGKGGKVGNGEDTFGDVESGCVIQMTSLDADPSSMADFFREIGVVQGKVNDVKILLLKLQSAHE
KTKGTHKASELKEIRAEMDGDIESVTKAAQFMKFKLAELSKSNLANRQVKGCEEGTTTDRQRMALTNSQRKKLKEL
MDEFQALRATMMDEYKETITRRYYNVTGKQADEETTENMIRTGESETFLQQAIRQQGRGQLIETIREVQERHDGV
KEIERHFMEIHNIFTDISVLVDAQGQMVNEIQDNINRATSFTHRGADQLATARRRQIRKRKWTCVSILLLIVLILVLIIA
LKIAKIIP
>A9RDB4|SYP123_PHYPA-PHYSCOMITRELLA
MNDLLARGLNRGVRYDEDDRQNDLENGVKHFPPSVQLTNMKANTDGMGDFLRHIEVVQAEVNKMNQQLVSL
QNVNEKSKGVYRADELKALRAQMDAEIASATKRARFIKVKLEELDRSNIEHRQVRGCEAGTASDRQRISLTENQRK
KVKELMDAFQSLRSKMVDGYKETIERRYYTITGEQADEETIENLISTGESETLLQQAIREQGRGPVLEAVREIQERLD
GVKEIEKHMLELHAIFMDISVLVSAQGDMINDIESNVQRSYSYIKKGGEHLEVAKRYQMSKRRTTIICVLLLIIIIAILVL
VLVLKFK
>B9H229|SYP123_POPTR-POPULUS
MNDLFSSSFKKYTDLKQQAQMDDMEAGKESMNLDRFFEDVENVKEDMKTVERLYKSLQEANEECKTVHNAKT
MKNLRSRMDIDVEQVLKRVKIIKGKLEALDRSNAAHRNIPGCGPGSSADRTRTSVVSGLGKKLKDLMDNFQDLRA
RMAAEYKETVERRYFTITGERASEETIENLISSGESESFMQKAIQEQGRGQILDTISEIQERHDAVKEIEKNLIELHQVF
LDMAALVEAQGHQINDIESHVAHASSFVRRGTEQLSEAREYQKSSRKWTCIAIVAGAVLIIVLLLPFIPHLLALL
>B9HYZ9|SYP123_POPTR-POPULUS
MNDLFSSSFKKYTDLKQQAQIDDMEAGKEGMNLDRFFEDVENIKEDMKTVERLYRSLQEANEESKTVHNAKTMK
NLRSRMDMDVEQVLKRVKIIKGKLEALDRSNAAHRNIPGCGPGSSTDRTRTSVVSGLGKKLKDLMDNFQDLRAR
MAAEYKETVERRYFTITGERASDETIENLISSGESESFMQKAIQEQGRGQILDTISEIQERHDAVKEIEKNLIELHQVFL
DMAALVEAQGHQLNDIESHVAHASSFVRRGTEQLQEAREHQKSSRKWTCIAIIAGVVLIVVMLLPFLPQILALL
>C5Z572|SYP123_SORBI-SORGHUM
MNDLFSSSSFKKYADSSPATSAGGTDAGSESVVNLDKFFEDVEAVKEDMRGLEGMYKGLQSTNEETKTAHDARTV
KSLRSRMDKDVEQVLRRAKAIKGKLEELDRSNATSRKVPGCGPGSSTDRTRTSVVAGLGKKLKDLMDDFQGLRAR
MAAEYKETVARRYYTVTGEKPEDSTIEALISSGESESFLQKAIQEQGRGQVMDTISEIQERHDAVKDIERSLMDLHQ
VFLDMAALVEAQGHQLNDIESHVAHASSFVRRGTVELESAREYQKSSRKWMCIAILASIVLIAVLVLPVLVNLRILTL
PTKR
>F6H865|SYP123_VITVI-VITIS
KSVKMNDLFSSSFKKYTDLKQQTYMDDMESGKEAVNLDKFFEDVENVKQDMGGVEKLYKQLQDANEESKTVHN
AKTMKDLRARMDSDVTQVLKRVKMIKGKLEGLERSNAASRNVPGCGPGSSADRTRSSVVSGLGKKLKDMMDDF
QGLRAKMSTEYKETVERRYYTITGQKADEDTIENLISSGESESFLQKAIQEQGRGQIMDTISEIQERHDAVKEIEKNLI

ELHQVFLDMAALVEAQGQQLNDIESHVAHASSFVRRGTEQLQVAREYQKGSRKWTCIAIILGAIVVGLLLLPILPTLI
SMLPK
>D7TEK3|SYP123_VITVI-VITIS
MNDLFSSSFKRFTDLKPQSFDVEAGGDAGRESVNLEKFFEEVENVKDDMRAVENFYKKLQDLNEESKTVHNAKT
MKDLRARMDTDVVQVLKRVKIIKGKLEALERSNAANRNHPGCGPGSSADRTRTSVVGGLGKKLKDMMDDFQNL
RVRMNAEYKDTIERRYFTITGEKADEETIENLISSGESETFLQKAIQDQGRGQIMDTISELQERHGAVKEIEKNLIELH
QVFLDMAALVEAQGQHLNDIESHVAHASSFVRKGTDQLQIARNYQKSSRKWTCIAVGLAICLIIVILFPVLKSLDMI
HL
>O64791|SYP124_ARATH-ARABIDOPSIS
MNDLFSSSFKKYTDLKQQAQMDDIESGKETMNLDKFFEDVENVKDNMKGVETLYKSLQDSNEECKTVHNAKKVK
ELRAKMDGDVAQVLKRVKMIKQKLEALEKANANSRNVSGCGPGSSTDRTRTSVVSGLGKKLKDLMDSFQGLRAR
MNAEYKETVERRYFTITGEQADEQTIENLISSGESENFLQKAIQEQGRGQILDTISEIQERHDAVKEIEKNLIELHQVFL
DMAALVESQGQQLNDIESHVSKASSFVRRGTDQLQDAREYQKSSRKWTCYAILLFIVVFALLLIPALPHIMLMLK
>Q9SXB0|SYP125_ARATH-ARABIDOPSIS
MNDLFSNSFKKNQAQLGDVEAGQETMNLDKFFEDVENVKDDMKGVEALYKKLQDSNEECKTVHNAKKVKELRA
KMDGDVAMVLKRVKIIKQKLEALEKANANSRNVPGCGPGSSTDRTRSSVVSGLGKKLKDLMDSFQGLRARMNNE
YKETVERRYFTITGEKADEQTIDNLIASGESENFLQKAIQEQGRGQILDTISEIQERHDAVKEIEKNLLELHQVFLDMA
ALVEAQGQQLNNIESHVAKASSFVRRGTDQLQDAREYQKSSRKWTCYAIILFIVIFILLLIPLLPHIMLMLK
>A0A1I9LQP3|SYP131_ARATH-ARABIDOPSIS
MNDLLKGSLEFSRDRSNRSDIESGHGPGNSGDLGLSGFFKKVQEIEKQYEKLDKHLNKLQGAHEETKAVTKAPAMK
SIKQRMERDVDEVGRISRFIKGKIEELDRENLENRTKPGCGKGTGVDRTRTATTIAVKKKFKDKISEFQTLRQNIQQE
YREVVERRVFTVTGQRADEETVDRLIETGDSEQIFQKAIREQGRGQIMDTLAEIQERHDAVRDLEKKLLDLQQVFLD
MAVLVDAQGEMLDNIENMVSSAVDHVQSGNNQLTKAVKSQKSSRKWMCIAILILLIIIIITVISVLKPWTQKNGA
>F4K9K2|SYP132_ARATH-ARABIDOPSIS
MNDLLKGSFELPRGQSSREGDVELGEQQGGDQGLEDFFKKVQVIDKQYDKLDKLLKKLQIYDSVASQLPCASHEES
KSVTKAPAMKAIKKTMEKDVDEVGSIARFIKGKLEELDRENLANRQKPGCAKGSGVDRSRTATTLSLKKKLKDKMA
EFQVLRENIQQEYRDVVDRRVYTVTGERADEDTIDELIETGNSEQIFQKAIQEQGRGQVMDTLAEIQERHDAVRDL
EKKLLDLQQIFLDMAVLVDAQGEMLDNIESQVSSAVDHVQSGNTALQRAKSLQKNSRKWMCIAIIILLIVVAVIVVG
VLKPWKNKSA
>I1H3H3|SYP132_BRADI-BRACHYPODIUM
MNNLLTDSFELPRRDSSRDGDIEMGMHQADASDNLKGFLKKVDGIEGLIAKLTNLLTKLQTANEESKAVTKASAM
KAIKQRMEKDIDEVGKIARTAKTKVDELEKDNLSNRQKPGCGKGSAVDRSREQTTGAVKKKLKERMDDFQVLRESI
RQEYREVVERRVFTVTGNRPDEETIDDLIETGRSEQIFKDAVQQQGRGQVLDTVAEIQERHDAVRDLERKLLELQQI
FLDMAVLVEAQGDMINHIETHVSNATNHIQQGVGALQNAKKLQKNSRKWMCYAIILLLVIVAIIILAVIQPWKK
>I1MNV4|SYP132_SOYBN-GLYCINE
MNDLLTESFEIPRGQGHGGGGDIELGEYARNSGDLGLDSFFKKVQELDKQYAKLDKLLKKLQDAHEESKAVTKAPS
MKAIKQRMEKDVDEVKKTAHYLKTKIEELDKENLANRQKPGCGKGSGVDRSRTATTISLKKKLKDKMAEFQTLREAI
HQEYREVVERRVFTVTGTRADEETIDRLIETGDSEQIFQKAIQEQGRGQIMDTLAEIQERHEAVRDVERKLLDLQQIF
LDIAVLVDAQGDMLDNIETQVSSAVDHVQQGNNALQKAKKLQRNSRKWMCIAIMILLIVVIIIVVAVIKPWVTKKG
A
>Q0D8F0|SYP132_ORYSJ-ORYZA
ATPRLTLSLSLSSPPPPPPRFSLLAARRRDLAAMNNLLTDSFELPRGGSSRDGDIEMGMQADPSDNLKGFLKKVDAI
ESLIAKLTNLLHKLQTANEESKAVTKARDMKAIKQRMEKDIDEVGKIARMAKTKVDELEKDNLSNRQKPGCGKGSA
VDRSREQTTGAVKKKLKERMDDFQVLREAIRQEYRDVVERRVFTVTGSRPDEETVDNLIETGRSEQIFQEAIQQQG
RGQILDTVAEIQERHDAVRDLERKLLELQQIFMDMAVLVDAQGDMINNIETHVSNATNHIQQGVSALQNAKKLQ
KNSRKWMCYAIILLLIIVVIIVVAVIQPWKKGA
>B9I5W3|SYP132_POPTR-POPULUS
MAYVHVHPARSMNYQLEIFVEDEHEGFYFLYSLGMQIFLDMAVMVDAQGDVLDSIELNGSCALQKAKKLQRNSR
KRMCIAIIILLITVVIIVVTVIKPWDNNKGA >B9IPR8|SYP132_POPTR-POPULUS
TRICHOCARPAMNDLLSESFEIPRGQGSRGGDIEMGMNSADLGLESFFKKVQEIEKQNEKLDKLLKKLQDAHEESK
AVTKAPAMKGIKQRMEKDVDEVGKIARSIKSKLEELDKENLSNRQKPGCGKGTGVDRSRTSTTIALKKKLKDKMAE

FQTLRENIHQEYREVVERRVFTVTGTRADEETIDTLIETGDSEQIFQKAIQEQGRGQITDTLAEIQERHDAVRDLERKL
LDLQQIFLDMAVLVDAQGDMLDNIESQVSNAVDHVQSGNVALQKAKKLQRNSRKWMCIAIIILLIIVAIIVVTVLKP
WNNNKGA
>C5XAA1|SYP132_SORBI-SORGHUM
MNNLLTDSFELPRRDSSRDADIEMGMHQADASDNLKDFLKKVDAIESLIAKLTNLLNKLQTANEESKAVTKASSMK
AIKQRMEKDIDEVGKIARQAKTKVDELEKDNLLNRQKPGCGKGSAVDRSREQTTGAVKKKLKERMDDFQTLREAI
RQEYREVVERRVFTVTGNRPDEETIDDLIETGKSEQIFKDAIQHQGRGQILDTVAEIQERHDAVRDLERKLLELQQIF
MDMAVLVEAQGDMINNIETHVSNATNHIQQGVTALQSAKKLQKNSRKWMCYAIILLLVIVVVIVVAVIQPWKKG
>D7U8L0|SYP132_VITVI-VITIS
MNDLLSESFEIPRGQASREGDIELGERALQNSGELGLENFFKKVQEIEKQNDKLNVQLKKLQDAHEESKAVTKAAA
MKAIKKRMEKDVDEVGKIARSIKAKVEELDKENLANRQKPGCGKGTGVDRSRTATTVALKKKFKDKMAEFQVLRE
SIHQEYREVVERRVFTVTGTRADEETIDRLIETGDSEQIFQKAIQEQGRGQIMDTLAEIQERHDAVREVERKLLDLQQ
IFLDMAVLVDAQGDMLDNIESQVSSAVDHVQSGNTALQRAKKLQRSSRKWMCIAIIILLIIVVIIVVAVLKPWSKNG
A
>Q1LYX4|SEC20_ARATH-ARABIDOPSIS
MDEVVVEVEKTKREWEEAYEKTIGHIISIQQYGKSRRGDGGEEKFSLQRLNGLAQDGLSLLNSLQFNLDLLAPQLPS
DDQVQSTQSLLETWKNQYHSLRVNLRSANLQAKDNMRKAAQEERELLLGGGTESTVLRRKRQANAGVTSDAESI
TESLRRSRQLMVQEVERSTNTLVAFDESTGVLKKAESEYKGHRSLLSRTRNLLSTMQRQDVIDRIILIVGFSLFVFAVV
YVVSKRIGILKLQRMATAAIKAQLAGKAANGVGDDVMPLGQQFDGNTVPTVNIPLQQRMHDEL
>I1HL04|SEC20_BRADI-BRACHYPODIUM
MDEVTQAVENLKKEWSQVVTQLELCVAAIESCGKMGKGTEEASSLPRLNGSAQDSLQLLNALQCRLDLLVEQLPS
FEEVQSGQATLGSWKEQYQKLRASLRSANLQAKSNIKKAAQEERELLLGGGEESTIRRRNLQTKTGLTSAAESITESL
RRSRQLMVQEVERSASTLSTFDESTSVLRKAEGEYQGHRSLLMRTRGLLSTMQRQDVLDRIILTIGFLIFFLAVLYVVS
RRIGLLTLQRKLADAIRSGSISAEDIIPKVQHGPAPANPNIAPPIYDEL
>I1HL05|SEC20_BRADI-BRACHYPODIUM
MDEVTQAVENLKKEWSQVVTQLELCVAAIESCGKMGKGTEEASSLPRLNGSAQDSLQLLNALQCRLDLLVEQLPS
FEEVQSGQATLGSWKEQYQNCSPLFIGWCCHFIRITWIAIQDCPISQRFFKHCECRVPAI
>A8I967|SEC20_CHLRE-CHLAMYDOMONAS
MSVASLAAGADVSLFGPDELQVHQQLLKLERDIQVATDRIANSAASSANAPAVTDKQLQELLSSVRGQIRDFELLA
EEQDTDEHTDAVEACVELHQAEYTRLAAGIAAAKGQAKRQQQQTAEQQRRELFAGASLPVLQREYKSVAEAVQA
TSEVTESLQRARALLTQQIDQTGATMAVLDSSNNTLGQAKDEFVGQKQLNKKGAKLLGTIQKQEKHDRRLLWLGL
LLFLLTAGYIGYKRAPAVVRAPVDMALGSAAGAARAVWKQVGAGRLGHWG
>I1L097|SEC20_SOYBN-GLYCINE
MDKVVEEVEKVKKEWDETYKKTQEHIEAIADYGKSARAKEENNSLARLNGIAQDGLALLSSFLFTLDLLAPQLPSEPE
VQSTRALLQSSKTLTQNLRLNLRNANLQAKANLRKAAQEERELLLGGGEESTVRRRNLQTKAGMTSAAESITESLRR
TRQLMVQEVERNTSTLMTLDESTGVLKKAESEYKGHRSLLMRTRNLLSTMQRQDVIDRVIIGVGFLLFSLAVLYVVS
KRIGLLTLQRKVTEAIKAGMVGQAELRPQAVADDVNLHQVRGNRVPNNAEAPLEQRIHDEL
>C6TKD1|SEC20_SOYBN-GLYCINE
MDKVVEEAEKVKKEWDETYKKTQEHIEAIAEYGKPGRAKEEKNSLARLNGIAQDGLALLSSFLFTLDLLAPQLPSEPE
VQSTRALLQSWKTLTQNLRLNLRNANLQAKANLRKAAQEERELLLGGGEESTVRRRNLQTKAGMTSAAESITESLR
RTRQLMVQEVERNTSTLMTLDESTGVLKKAESEYKGHRSLLMRTRNLLSTMQRQDVIDRVIIGVGFLLFSLAVLYVV
YKRIGLLTLQRKVTEAIKAGMVGQAELRPQAVADDLNLHQVRGDCVHNAEAPLEQRIHDEL
>Q6H674|SEC20_ORYSJ-ORYZA
MDKVTQAVENLKEEWNQAVAQLEGCIAAIESCGKMGKGTEEASSLPRLNGSAQDALQLLNSLQCRLDPLAEQLPT
FEEVQSGQATLQSWKEQYQKLRMRLRNANLQANANIKKAAQEERELLLGGGEESTIRRRNLQTKAGMTSAAESIT
ESLRRSRQLMVQEVERSANTLATFDESTSVLRKAEGEYQGHRSLLMRTRGLLSTMQRQDVLDRIILTIGFLIFSLAVLY
VVSRRIGLLTLQRKLADAIRSGSISAEDVVAKVKNVPVPAPAAPAPAPPIYDEL
>A9SW43|SEC20_PHYPA-PHYSCOMITRELLA
MVREDNSFVFGTEDDDDEAIVAAKGKELEGAWAETKQEVQKQMQLLASFGTAGGVADAAMVPRTSALLQDHIA
NLRTLIVRYEMIAQQYCTEEGVQAAMRTVQEWKDQIQAWVHHILSLFSGVFPQIDTFIYACRLRMSSRNANLQAK

RNIDQADQSNATLRKADAEYKGQRSILGVTRSKITSITRQDLIERCKVLFGLQVKLHAVVQNVGVFQIESGCSQMEF
DVPSCNSGLRVPHVELEDRTLLHIKNQNEFILKSAQYSN
>A9S7T6|SEC20_PHYPA-PHYSCOMITRELLA
MDDVERNFDFGEEDDNGAVVIAKGKELENSWAETTQEVQKQMQLLASFGTVGGMADVAMVPRTNALLQDHIA
KLRALIVRYEMIAQQYPTEEGVQASMRTVNDWKDQIQALRMSSRNANLQAKRNIDQAVMSEREQLLSGGQDAD
LRRKQLQTKAGMTSAAEGITDGLRRTRQIMIQEVERNAATLGVLDQSNATLRKADAEYKGQRSILGVTRSKINSMT
RQDLIDRALVIFFVFVFISVCFYITLRRLPIVKHYVRGSSILPLHQPLLKDSAPPHSPPSYPQAPPRDSLSRGEFHEPDVY
DTAPQFHEHQGAQSQVFTARGAPEYVLQTAHDSAKDPWYLEEL
>A9PBC7|SEC20_POPTR-POPULUS
MDEVVEAVEKAKKEWEETYSKAQQHMKAIQDYDAISSSSSSSVSLPRLNGLAQDCLALLQSLQFQLDLLAPQLPTD
DQLKSALRLLDSWKQLYHDLRASLRNANLQAKANMRKAAQQERELLLGGGEESTIRRRNLQTKAGMTSAAESITE
SLRRTRQLMVQEVERSTGTLMTFEESTTVLRKAETEYKGHRSLLSRTRNLLSTMQRQDVLDRVILAVGFFLFSCAVLY
VVSKRIGVLALQRKVTAALKAGMAGKGAIKARAVEDAIPRVIEDGIDVAQVLDNAVPKVEVPVEQAMHDEL
>C5WUD4|SEC20_SORBI-SORGHUM
MDEVTQAVENLKKEWSQAVSQLEENITAIESCGKTGKGTEEANSLPRLNGSAQDALQLLKSLQFRLDLLAQQLPTF
DEVQSGQATLKSWDEQYKKLRVSLRNANLQAKDNIRQAAQEERALLLGGGEESTIRRRNLQTKAGMTSAAESITES
LRRSRQMMVQEVERNASTLATFDESTSVLRKAEGEYQGHRSLLMRTRGLLSTMQRQDVLDRIILTIGFIIFSSAVLYV
VSRRIGLLTLQRKLANAIRSGSLSAEDIVAKAQHGPVALANAPAPPPIYDEL
>C5XZH4|SEC20_SORBI-SORGHUM
MDEVTQAVEDLKKEWSQAVSQLEESIAAIETCGKTGKGTEEATSLPRLNGSAQDALQLLKSLQFRLDLLAQQLPTFD
EVQSGQATLESWDEQYKKLRASLRNANLQAKENIRKAAQEERELLLGGGEESTIRRRNLQTKAGMTSAAESITESLR
RSRQMMVQEVERSASTLATFDESTSVLRKAEGEYQGHRSLLMRTRGLLSTMQRQDVLDRIILTIGFIIFSLAVLYVVS
RRIGLLTLQRKLANAIRSGSLSAADIVAKAQHVPAPANVPAPIYDEL
>D7SZQ1|SEC20_VITVI-VITIS
MDKVTEAVEKAKKDWEETYGKTLDHIKAIEDYGKSSDQKNSLPRLNGLAQDGLALLSSLQFNLDLLAPQLPTDEDIQ
SANTTLQSWKNQIQSLRSSLRNANLQAKANMRKAAKEERELLLGAGGESTIRRRNLQTKTGMTSAAENITESLRRT
RQLMVQEVERNTTMLLTNEESTGVLKKAESEYKGHRSLLMRTRNLLTTMQRQDVLDRIILIVGFVFFSCAVLYVVSK
RIGLLKLQRTVTAAIKAGMVRQANIGQDAAEDGINLAPVHDNAIRRVEVPLEQHMHDEL
>Q9SJL6|MEMB11_ARATH-ARABIDOPSIS
MASGIVEGGGSLSDVYSSAKRILLKARDGIERLERFESSSMDSPDLASSVKRDITEVRSLCSNMDTLWRSIPVKSQRD
LWRRKTEQVGEEAEYLNLSLEKYMSRNQRKMLEAKERADLLGRASGEGAHILQIFDEEAQAMSSVKNSKRMLEES
FSSGVAILSKYAEQRDRLKSAQRKALDVLNTVGLSNSVLRLIERRNRVDTWIKYAGMIATLVILYLFIRWTR
>A8IYN5|MEMB11_CHLRE-CHLAMYDOMONAS
MSDLTSLHSQAKRLILLLREGLERLEALEGATRHQPGTDTTSALARDLRSQLQQLARISTEMDSIWRMQVIRENASK
RDVWKRKVEQVSEELDTLRVAMDRHGSRESKRAAEARDREDTINTTTTHRQQEMDEEAQVMGSVARSKRYLEE
MFESGTNILVNMAGNRERLKSAQKRALDVLNTVGLGESLLRLIERRQRMDMWTAYGGMIVITLVVCLCVWWFW
F
>I1KXX2|MEMB11_SOYBN-GLYCINE
MEGGGGTLSEIHQSAKKLLLRSRDGLERLERLEYSAAAGAAFSGADSELSFAVKKDITQIQTLCVEMDRLWRSIAAK
PQRDLWKRKVEQIAEEAESLRASLDKYNLRNQKRMREANERTELLGRANGDSAHVLRIYDEEAQALQSVRSSSREL
ENANALGEAILSSIHGQRERLKSAHRKALDILNTVGISNSVLRLIERRNRVDQWIKYAGMLLTVVFLFAFIMWRH
>I1N120|MEMB11_SOYBN-GLYCINE
MEGGGGTLSEIHQSAKKLLLRSRDGLERLEYSAPAAAAVSGADSELSFAVKKDITQIQSLCVEMDRLWRSIAAKPQR
DLWKRKVEQIAEEAESLRASLDKYNLRNQKRMREANERAELLGRANGDSAHVLRIYDEEAQALQSVRSSSQELENA
NALGEAILSSIHGQRERLKSAHRKALDILNTVGISNSVLRLIERRNRVDQWIKYAGMLLTVIFLLAFIMWRH
>Q6AT61|MEMB11_ORYSJ-ORYZA
MDFSGGGGGGGGATLSEMYQSARRLLLSARDGVARVERLASAPTSSSYSSAPLVGGGGGAGDSAAAEEVRREVA
QIQGLCAQMDRLWRSIPAKGQRDLWKRKVEQLSEEVDSLKETLDRHSLRQKKRVLEAKERAELFERANGESSHVLR
IFDDEAQAMQSARSSSRMLDEAYETGVAILHKYADQRDRLKSAQRKALDILNTVGLSNSVLKLIERRHRVDKWIAY
AGMMITVVVMFVFWRWTH
>A9RIA8|MEMB11_PHYPA-PHYSCOMITRELLA

MEDHGGAVGGLSLEKVHSRARRALLMVRDGLERLERLELAAQQPSSSSSPSYRGIGGLQDAQPTAPDIVENLKKEI
GELQVASADMDRMWRNQVLAKGQRDLWKRRIEQVAEEVESLKAGIDRYLLRQHRRQVEAQERAELFRRTRGDG
AHILQVHDIEMQALQSAKNSSRMLDDAYATGVAVLGQYAVQRDRLKSAQRKAYDVLNSVGLGNKMMRMIERR
HKVDRCIAYGGMVFTIIIVLFVIRWVR
>A9SG88|MEMB11_PHYPA-PHYSCOMITRELLA
MQGVGGGMPLDRVYSQARRALLMVRDGLERLERLEMAGQHPSSSSTAPDLVEHLKKEIGQLQMSSADMDRM
WRNQILAKGQRDLWKRRIEQVAEEVESLKAGLDRYLMRQHRRQVDAQERAELFRRARGDGAHIMQVHDLDMQ
ALQSAKNSSRMIDDAYATGVAVLAQYAVQRDRLKSAQRKAYDLLNTVGLGNKMMRIIERRHKVDRWIAYGGMF
VTIVILVVVIRWVR
>A9SG86|MEMB11_PHYPA-PHYSCOMITRELLA
MFGVLSMEALDYKRRYSQGACSGSRRRMDDAGRNLKEQLKSVEEQIRGLKECIGRVEEEIFEVHDLDMQALQSAK
NSSRMIDDAYATGVAVLAQYAVQRDRLKSAQRKAYDLLNTVGLGNKMMRIIERRHKVDRWIAYGGMFVTIVILV
VVIRLFFTANTSLLTNYVHFFAQHFCPFGAADMLKFYENITAGFACLFAGFHMRVCTRSLS
>C5WQH7|MEMB11_SORBI-SORGHUM
MEYSGGGATLSEMYQSARRLLLSARDGVARVERLASAPTSSSYSSAPLVGGGAAGDPAVAEEVRREVAQIQGLCA
QMDRLWRSIPAKGQRDLWKRKVEQLSEEVDSLKETLDKHSLRQKKRILEAKERAELFERANGESSHVLRIFDDEAQ
AKQSARNSSRMLEEAYETGVAILHKYADQRDRLKSAQRKALDVLNTVGLSNSVLKLIERRHRVDKWIAYAGMIITV
VVMIAFWRLTH
>D7TL48|MEMB11_VITVI-VITIS
MEGGGTTFSEIYQNSKKLLLRTRDGLERLERLEFSSSNPVDSPELAFAVKKDISQIQSLCVEMDRLWRSISAKSQRDL
WKRKVEQVAEESESLKESLDKYFLRHQKRMMEAKERAELLGRANGDSAHVLRIFDEEAQAMQSARNSSMMLEEA
YSKGVAILTKYADQRDRLKGAHRKALDVLNTVGLSNSVLKLIERRNRVDKWIKYTGMVVSVVVLYTFWRWAH
>Q9FK28|MEMB12_ARATH-ARABIDOPSIS
MASGTVGGLSEVYSSAKRILLRARNGIEKLERFDSDPTDLASSVKRDITEVQSLCSNMDGLWRSIPVKSQRDLWRRK
SEQVGEEAEYLNQSLEKYMWRNQRKMLEAKERADLLGRGSGEGAHILQIFDEEAQGMNSVKNSKRMLEDSFQS
GVAILSKYAEQRDRLKSAQRKALDVLNTVGLSNSVLRLIERRNRVDTWIKYAGMIATLVILYLFIRWTR
>Q9LMP7|GOS11_ARATH-ARABIDOPSIS
MDVPSSWDALRKQARKIEAQLDEQMHSYRRLVSTKALSKSDGNESDLEAGIDLLLRQLQQVNAQMQAWVSSGG
SEMVSHTLTRHQEILQDLTQEFYRHRSSLRAKQEHASLLEDFREFDRTRLDLEDGYGSEQALIKEHMGINRNTAQM
DGVISQAQATLGTLVFQRSTFGGINSKLSNVASRLPTVNTILAAIKRKKSMDTIILSLVAAVCTFLIFIYWITK
>I1IQ54|GOS11_BRADI-BRACHYPODIUM
MEASSWDALRKQARRLEAQLDDQMIAYRKLVSMKSDGSENDIESDIERSLKQLQQVNSQMQTWVSSGGSEVLS
HTLTRHMEILQDLTQEFYRLRSSLRVKQQHASLLDLRDFDRAKFDVEEAGDSADQALLREQAAISRNSGQVDNVIS
QAQATLGALMSQRSTFGGITTKISNVSSRLPTVNHILASIRRKKSMDTIILSLVASVCAFLIFIYWLSK
>A8J213|GOS11_CHLRE-CHLAMYDOMONAS
MSYLLEVPSTGRVATAYMAYRSPAPARPPGQTRVWEDLRKEARRLEGELDVKLAAFTKLCSSFEASYKLNTADNSL
GADQQLAQTKAAEVEDLLQRLSDINDEMAAIVGGSTDSRSHTLARHRDILQEFTQEFRKVNSTLGAALDRVKLLAG
ASDSPHLSVNVQNSSGALLRERGAIQNSANMVDDILSQAANVSGNLLGQRRVFEGALDKLVQVGSRFPVVNGLL
NAIRRKKSKDTLVLAGVIAACVLFTILYVMAK
>I1KGV3|GOS11_SOYBN-GLYCINE
MIDSRNLPLPNLLQARKLEAQLDEQMNSYRKLVSANVSTKADIAESDLGSWIERLLKQLQQVNTQMQAWVSSGG
SEMVSHTLTRHQEILQDLTQEFYRLHSSVKAKQEHASLLEDFKEFDRTRLDLEQGVDSEQHALLKERSSISRSAGHM
DNVISQAQASLGALVFQRSTFGGINSKLGNVSSRLPTVNNILSAIKRKKSMETIILSLVSAVCTFLIFMYWLSK
>Q6EQ07|GOS11_ORYSJ-ORYZA
MEASSWDALRKQARRLEAQLDDQMIAYRKLVSMKSDGSENDIESDIERSLKQLQQVNSQMQTWVSSGGSEVLS
HTLTRHMEILQDLTQEFYRLRSSLRVKQQHASLLDLRDFDRAKFDVESGDSADQALLREQAAISRSSGQMDNVISH
AQATLGTLMSQRSTFGGITTKISNVSSRLPTINHILASIRRKKSMDTIILSLVASVCAFLILVYWLSK
>Q6Z9I2|GOS11_ORYSJ-ORYZA
MEASSWDALRKQARRLEAQLDDQMSAYRKLISMKSDGSENDIESDIERSLKQLQQVNSQMQTWVSSGGSEVLS
HTLTRHMEILQDLTQEFYRLRSSLRAKQQHASLLDLRDFDRAKFDVEEGADSDQALLKEQAAISRSTGQMDTVISQ
AQATLGTLMLQRSTFGGITTKISNVSSRLPTINQILSSIKRKKSMDTIILSLVASVCAFLIFIYWMSK

>A9RLI3|GOS11_PHYPA-PHYSCOMITRELLA
MASGAGWSPKARNAGWDDLRKQARKLESELDVKLASFRRIGTPKDGQGDGSEAEIEKLLQHLNEVNKDMQNW
VSNAGSDVLSHTLARHRNILHELSQEFARIRVNAKVNREHAELLQHFSRGDERNSVMDDGGFGLQQQALLREQG
AISRSTSQMDSMIGHAHETFSALRYQRSTFGDISGKINTIGSRLPSVNGVLTAIRRRRSRDTIIIGSVASLCTILILLYWIT
K
>A9U109|GOS11_PHYPA-PHYSCOMITRELLA
MAMALGAGWTPDAGSVGWDELRKQARRLESELDVKLASFQRGQNAPAVDGQTDGNEVEIERLLQHLNDVNVR
MQNWVSDAGSDVLSHTLVRHQNILHELSQEFARIRVTANANRERAQLLQHFGGTGESKGFLDDRGNGLQSLFRE
QANINRSTAQIDSVIGHAQETYTALRYQRSTFRDITSKIGAISTRMPSVNKVLTAIRRRKSRDTFIVGAVTVFCLVMLL
LYWVAK
>B9GMC1|GOS11_POPTR-POPULUS
MEVTTTSSWDALRKQARKLEAQLDEQMNSYRKLASSKGSTKVDSAENDPESGIDRLLKQLQQVNSQMQAWVSS
GGSEMVSHTLTRHQEILQDLTQEFHRLRSGMRAKQEHALLLEDFREFDRTRLDLEDGVGSADQALLREHASISRNT
GQMDNVISQAQATLGSLVLQRSTFGGINSKLSNVSSRLPTVNQILSAIKRRKSMDSIILSLVASVCTFLIFIYWVTK
>B9GVJ3|GOS11_POPTR-POPULUS
MEVTTSSSSSWDALRKQARKLEAQLDEQMSTFRKLASSKGSTKVDFPENDLESGIDRLLKQLQQVNSQMQAWVS
SGGSEMVSHTLTRHQEILQDLTQEFHRLRSGMRAKQEHALLLEDFREFDRTRLDLEDGVGSADQALLREHASISRN
TGQMDNVISQAQSTLGALVLQRSTFGGINSKLSNVSSRLPTVNQILSAIKRKKSMDAIILSLVASVCTFLIFIYWLTK
>C5XCU1|GOS11_SORBI-SORGHUM
MEASSWDALRKQARRLEAQLDDQMIAYRKLVSMKSDGSENDIESDIERSLKQLQQVNSQMQTWVSSGGSEVLS
HTLTRHMEILQDLTQEFYRLRSSLRAKQQHASLLDLRDFDRAKFDVEDPSDSADQALLREQAAIGRSTGQMDNVIS
QAQATLGSLMTQRSTFGGITTKISNVSSRLPTINHVLSSIRRKKSMDTIILSLVASVCAFLIFIYWLSK
>D7SUY6|GOS11_VITVI-VITIS
MDPPSSWDALRKQARKLEAQLDEQMHLYRKLVSMKVDGDKEKEIDSGIDQLLKQLQQVNSHMQAWVSSGGSEI
FSHTLTRHQEILQDLTQEFYRLRSSFRAKKEHASLLEDFREFDRSRLDLEEGGGSEQALLKEHASISRSTGQMDTVISQ
AQATLGALVFQRSTFGGINSKLSNVSSRLPTVNNILSAIKRKKSLDTIILSLVASVCTFLILIYWLTK
>O22151|GOS12_ARATH-ARABIDOPSIS
MTESSLDLQESGWEELRREARKIEGDLDVKLSSYAKLGARFTQGDTDLVMNYEKVLKCVLVSGYVDTGSPTVGSGR
SWKSMEMEIQSLLEKLLDINDSMSRCAASAAPTTSVTQKLARHRDILHEYTQEFRRIKGNINSLREHAELLSSVRDDI
SEYKASGSMSPGVQVLRERASIHGSISHIDDVIGQAQATRAVLGSQRSLFSDVQGKVKNLGDKFPVIRGLLGSIKRKR
SRDTLILSAVIAACTLFLIIYWLSK
>I1HWS0|GOS12_BRADI-BRACHYPODIUM
MHGGGSSASDEAAAAALELQESGWEELRREARKLEGDLDVKLSSYARLAARSSSASASASAAASSSPTADRSSWKS
TELEIQALLDKLQDVNDAMSRCAAPAAPATSVSQKLARHRDILHEFTQEFRRTRGNLSSMREHADLLSSVRGDITES
KATGGMSPRVHLLRERSSIHGSINQIDEVIGQAQSTRSALSNQRALFGDVQGKVKQLGEKFPVVRGLLGAIKRKKSK
DTIILSAVIAACTIFLIIYWLSK
>I1J7S6|GOS12_SOYBN-GLYCINE
MRDPNLELQESGWEELRKEARKIEGDLDVKLSSYAKLGARFTQGGSGSGYVDSGSPPIGSSRSWKSMEMEIQSLLE
KLLDINDSMSRCAASAGPATSVTQKLARHRDILHEFTQEFRRIKGNINSMREHAELLSSVRDDITDFKTSGSMSPRM
QLLRERAAIHGSISHIDDVISQAQATRAVLGSQRTLFTDVQGKVKVLGDKFPMIRSLLGSIRRRRSRDTLILSAVIAAC
TLFLIIYWLSK
>Q6Z2M4|GOS12_ORYSJ-ORYZA
MMPSASDAAAAAAALELQESGWEELRREARKLEGDLDVKLSSYARLAARSSSAADAASASSPSERSSWKSMEFEI
QSLLDKLQDVNDAMSRCAASTAPTTSVSQKLARHRDILHEFAQEFRRTRGNLSSIREHADLLSSVRDDITESKATGG
MSPRVHLLRERASIHGSINQIDEVIGQAQSTRVALSNQRALFGDVQGKVKQLGEKFPVIRGLLGAIKRKKSKDTIILSA
VIAACTIFLIIYWLSK
>A9TGV1|GOS12_PHYPA-PHYSCOMITRELLA
MEDCEGGWEELRKEARKIEGDLDVKLSSYAKLGGDIRGDGSWKSMELEIESLLEKLLDVNDSMSRCVAAATSTTSV
TQKLARHRDILHEFTQEFRRTRNNISSMREHAELLTSVRNDISDHKASGNTSPVASLLRERGSIHGNIAQMDEVINIA
HATKGTLGAQRTTFTEIQGKVKQLGDRFPAIRGVLGAIKRKKSKDTLILAGVITGCTLFLIIYWLAK
>A9S3Y7|GOS12_PHYPA-PHYSCOMITRELLA

MEDCEGGWEELRKEARKIEGDLDVKLSSYAKLGGMLSHGGDARVEGSWKSMDTEIELLLEKLLDINDSMSRCVAA
ATSTTSVTQKLARHRDILHEFTQEFRRTRNNINSMREHAELLTSVRSDISDHKASGSSSPAASLLRERGAIHGNIAHM
DEVITIAHTTKVALGAQRTTFMEIQGKVKQLGDRFPAIRGVLGAIKRKKSKDTLILAGVITACILFLIIYWLSK
>B9GPD6|GOS12_POPTR-POPULUS
MTDPNLELQESGWEELRREARKIEGDLDVKLSSYAKLGARFTTQGGGYVEGGSPRVGSSRSWKSMEMEIQSSLEK
LLDINDAMSRCAAASAATSVTQKLARHRDILHEFTQEFRRIKGNINSMREHAELLSSVRDDISEYKASGSMSPRVHL
LRERAAIHGSIAHIDDVINQAQTTRAVLGSQRTFFGDVQGKVKVLSDKFPIIRGLLGSIRRRRSRDTLILSAVIAACTLF
LIIYWLSK
>B9IBX3|GOS12_POPTR-POPULUS
MTDTNLELQESGWEELRREARKIEGDLDVKLSSYAKLGSRFTQGGGGGGYVETGSPTVGSSRSWKSMEMEIQSSL
EKLLDINDAMSRCAASAAPATSVTQKLARHRDILHDFTQEFRRIKGNINSMREHSELLSSVRDDISEYKASGSMSPR
VQLLRERAAIHGSISHIDDVINQAQTTRAVLGSQRAFFGDVQGKVKLLGDKFPIIRGLLGSIRRRRSRDTLILSAVIAAC
TLFLIIYWLSK
>C5XT73|GOS12_SORBI-SORGHUM
MVSSSSSDAAAAALELQESGWEELRREARKLEGDLDVKLSSYARLAARSSSSAASASGAASPTADRSSWKSMEFEI
QSLLGKLQDVNDAMSRCAASSANTTSVSQKLARHRDILHEFTQEFRRTRGNLSSMREHADLLSSVRDDITESKATG
GMSPRVHLLRERASIHGSINQIDEVIGQAQSTRVALSNQRALFGDIQGKVKQLGEKFPIIRGLLGAIKRKKSKDTIILSA
VIAACTIFLIIYWLSK
>D7U7V6|GOS12_VITVI-VITIS
MDPNLDLQESGWEELRKEARKIEGDLDVKLSSYAKLGARFTQGGYVDTGSPTVSSSRSWKSMEMEIQSLLEKLLDT
NDAMSRCAASAAPTTSVTQKLARHRDILHEFTQEFRRIKGNMNSMREHAELLSSVRDDISEYKASGSMSPRMQLL
RERAAIHGSISHIDDVISQAQTTRAALNSQRTLFGDFQGKVKLLSDKFPIIRGLLGSIRRRRSRDTLILSAVIAACTLFLII
YWLSK
>Q9SEL6|VTI11_ARATH-ARABIDOPSIS
MSDVFDGYERQYCELSASLSKKCSSAISLDGEQKKQKLSEIKSGLENAEVLIRKMDLEARTLPPNLKSSLLVKLREFKS
DLNNFKTEVKRITSGQLNAAARDELLEAGMADTKTASADQRARLMMSTERLGRTTDRVKDSRRTMMETEEIGVSI
LQDLHGQRQSLLRAHETLHGVDDNIGKSKKILTDMTRRMNKNKWTIGAIIIALIAAIFIILYFKLTK
>Q1PDQ2|VTI11_ARATH-ARABIDOPSIS
MSNVFHEYDQQYSELSINLSKKCSLAFSLKGGEKKEKLSEITYDLENAEKLISKMDHAASNLPPNIKSILLEKLKESKSSL
KRLRNEIKRNTSENLKVTTREEVLEAEKADLADQRSRLMKSTEGLVRTREMIKDSQRKLLETENIGISILENLQRQKES
LQNSQAMLHEIDDTVKESRSIVRSIKIKEFFTVTAPIIIYFLFKLVK
>I1HH11|VTI11_BRADI-BRACHYPODIUM
MSEVFEGYERQYSEISASLSRKCAAASALDAEKKKQKLSEIQSDVQEAESLIRKMDLEARSLQPSVKAGLLAKLREYKS
DLNNIKSEIKRISAANAQQATREELLEAGMSDTLAASSDQRGRLMMTSERLNQSTDRIRESQRTVFETEEIGVSILQ
DLHNQRQSLLHAHTTLHGVDDYIGKSKKILASMSKRMDRNKWIVGGIIAALVLAILFILYFKFAR
>I1HQT7|VTI11_BRADI-BRACHYPODIUM
MSEVFESYERQYCEVSASLSRKCTAASALDGEKKKQRLSEIQSGVEEAESLIRKMDLEARSLQPSVKAGLLAKLREYKS
DLNNLKSELKRISAPNARQATREELLESGMADTLAVSTDQRGRLMMTTERLNQSTDRIKESRITMLETEECGVSILQ
DLHQQRQSLLHAHTTLHGVDDNVGKSKKILAAMSKRMDRNKWIIGGIIAALVLAILIILYFKLAH
>A8HTE8|VTI11_CHLRE-CHLAMYDOMONAS
MDDVSALFNQYENEYCNKSTDISRKITTASTLGAEAKRKKLVEVDVDIKEADTIIKKMDNEARSVAPDRQKQLQNK
VKEYKADLASLKEQLQKARSSTSDFEAGRAELGLGMDYASSSAAQRDRMLSATAKLEQSDERLKQGKALLAETEDL
GAGILANLHSQRETIVRSRDTLHGADDNITKARKILSSMSKRMLQNKLIMFGIIGTLLAAIILIIYFKTRK
>I1KCV4|VTI11_SOYBN-GLYCINE
MSEVFEGYERQYCEQSANLSRQCTAASALDGEQKKQKLSDIKAGLDDADTLIRKMDLEARSLQPSVKAALLAKLRE
YKTDLSNLKSEVKRVTSASVNLTARDDLLESGRADTLAASNDQKGRLLMSTERLNQSSDRIKESRKTMLETEDLGEFI
LRDLHQQRESLLHAHKTIHGVDDNISKSKKILSAMSRRMSRNKWIVSSLMTALVLAILIILYFKLTH
>K7LFP0|VTI11_SOYBN-GLYCINE
MSCGEFEGYERQYCELSANLSKACIDNVAAPLNGELKKQKKSEIKEGIEEGEALIRKMDLDARSLQPDLKAVLLAKVR
EYKADLNNIKREVKKIISADLNPSSARDELLESTMTNAMMKASADHRERERLMISTERLNKSSDRINDSRGTMLETE
DLGISILQDLHSQRQSLLHTHDTLHGVDDNTDKSKKILSNMSRRMDKSKWILSTIAVLLIFVIILIVYFKLS

>C6TGN3|VTI11_SOYBN-GLYCINE
MSNVFEGYERQYCELSANLAKKCTAAGVLNGEQKKQKVSEVKAGIDEAEALIRKMDLEARSLQPNIKGVLLAKLREY
KSDLNNLKSEVKKIVSGNLNPSARDELLESGMADAMTASADQRTRLMVSTERLNKTSDRVKDSRRTMLETEELGV
SILQDLHSQRQSLLHAHNTLHGVDDNIGKSKKILTNMSRRMNKNKWVIGGIVLVLIIAIIVILYFKLSK
>Q8S0N4|VTI11_ORYSJ-ORYZA
MSEVFEGYERQYCEVSASLFRKCTTASALDGEKKKQKLSEIQSGVEEAESLIRKMDLEARSLQPSVKAGLLAKLREYK
SDLNNLKSELKRISAPNARQATREELLESGLADTLAASTDQRGRLMMTTERLNQSNDKIKESRRTILETEELGVSILQ
DLHQQRQSLLHAHTTLHGVDDNVGKSKKILAAMSKRMDRNKWIIGGIIAALVLAILLILYFKLAY
>A9RCL7|VTI11_PHYPA-PHYSCOMITRELLA
MSEIFDGYERQYCELSANLSRKCTSVAALHGEEKKQKLAELKAGMDEADSLIRRMDLEARSLPPTQKATLLAKLREY
KSDLTNLKRDVKKSATTDAAAARDDLLESGMADRANPTHDQRGRLLMSTERLNQSGERIKESKRTLLETEELGVSIL
QDLASQRQTLLHAQNNLHGVDDNIGKSRRILNSMSRRMSRNKWIMGSIIGVLTLAIVFVIYVKVTK
>A9T6G0|VTI11_PHYPA-PHYSCOMITRELLA
MSEIFDGYERQFCELCTHIKKRCKSVSSNETEKKQKYDELKTGLDQAESLIRRMDLEARTLIPPLKIALLTKLREYKSDL
NVLKREAKNFLVSIDSLSARNELIDSRPGGLQSRNAVLQFEADQRDRLVNATDRINRSGEKVKESRRQLIETEDLGVR
ILQDLHVQHQTLLHTQQMMHGVDTNIAKSSRLLSSMSQRFEKHKYIMTGIIAVLFLAIMFVIYIKSHK
>A9U3W6|VTI11_PHYPA-PHYSCOMITRELLA
MSEVFDGYERQYCELSANLSRKCTSVSALHGEEKKHKLTELKSGLDEADSLIRRMDLEARSLPPTQKAALLAKLREYK
SDLNNLKRDVKKSASTDAASARDDLLESGMANHANPTHDQRGRLLMSTERLNQSGERIKESKRTLLETEELGVSIL
QDLASQRQTLLHAQNTLHGVDDNIGKSRRILNSMSRRMSRNKWIMGSIIGVLILAIVLVIYVKLMR
>A9SJ58|VTI11_PHYPA-PHYSCOMITRELLA
MGEIFDGYERQYCELSTNLNKKCNSISSNEVEKKQKFDDLTKGLDLAESLIKRMDLEARTLPPAQKATLLAKLREYKS
DLNVLKREAKKILASNDPFSARDELLNPGLVGRHLGVRLDQRDRLMMATDRMNQTGERIKESRRQLNETEEVGV
NILQDLQLQHQTLLHTQHTIHGVADGIAKSRRLLSSLSQRFERHKYIMGGIIAVCLLAILFIMHVKSGH
>A9P918|VTI11_POPTR-POPULUS
MTDIFEGYERQYCELSSNLSRKCTAALSLDGEKKKQKISEVKAGLEDAEALIRKMDLEARNLQPNVKAVLLAKLREYK
SDLNNLKTEVKRIGSGNLNAAARDELLEAGMTDALMASGDQRSRLMMATERLYHSSDRIKDSRRTMLETEELGVS
ILQDLHQQRQSLLHAHNTLHGVDDNIGKSKRVLTAISRRMNRNKWMISAIIAVLVVAVILILYFKLK
>A9P9H7|VTI11_POPTR-POPULUS
MNEIFEGYERQYCELSANLSRKCTAALALDREQKKQKISEIRAGLEDAESLIRKMDMEARNLQPNVKAVLLAKLREY
KSDLNNLKTEVKRIGSGNLNASARDELLEAGMANSLTASADQRSRLMMTTERLNQSGDRIKDSRRTMLETEELGV
SILQDLHQQRQSLLHAHDTLHGVDGNIGKSKRVLTAMSRRINKNKWIIGAVIAVLVVVISLILYFKLK
>C5XHN3|VTI11_SORBI-SORGHUM
MSEVFEGYERQYCEASASLSRKCTAASALDGEKKKQKLSEIQSGIEEAESLIRKMDLEARSLQPSIKAGLLAKLREYKS
DLNNVKSELKRISAPNARQATREELLESGMADTLAVSTDQRGRLMMTTERLNQSTDRIKESRRTMLETEELGVSIL
QDLHQQRQSLLHAHTTLHGVDDNIGKSKKILAAMSKRMDRNKWIIGGIITALVLAILLILYFKLAH
>F6H5V9|VTI11_VITVI-VITIS
MGEVFDGYERQYCELSANLSKKCTTASLLDGEQKRQQISEIKAGLDEADSLIRKMDLEARGLQPNVKAVLLAKLREY
KSDLNNLKSEVKRIASANSNQAARDDLLESGMADAMSVSANQRARLMMSTDRLNQSSDRIKESKRTMLETEELG
VSILQDLHQQRQSLLHANDTLHGVDDNVSRSKKILTNMSRRMSRNKCIIGSIIAVLVVVIALILYFKLSK
>F6GT59|VTI11_VITVI-VITIS
MSQVFEGYERQYCELSANLSRKCTAASLLNGEQKKQKVSEIKAGLDDADALIRKMDLEARSLQPSVKAMLLAKLRE
YKTDLNNVKNEVKRITSANTNQAARDNLLESGMADTMTVSADQKTRLLMSTGRLNQSGDRIKESRRTMLETEELG
VSILQDLHQQRQSLLHAHNTLHGVDDNISKSKKILTAMSRRMSRNKWIIGSVIAALVLAIILILYFKLAH
>Q9SEL5|VTI12_ARATH-ARABIDOPSIS
MSDVFEGYERQYCELSTNLSRKCHSASVLSNGEEKKGKIAEIKSGIDEADVLIRKMDLEARSLQPSAKAVCLSKLREYK
SDLNQLKKEFKRVSSADAKPSSREELMESGMADLHAVSADQRGRLAMSVERLDQSSDRIRESRRLMLETEEVGISI
VQDLSQQRQTLLHAHNKLHGVDDAIDKSKKVLTAMSRRMTRNKWIITSVIVALVLAIILIISYKLSH
>I1J5U5|VTI12_SOYBN-GLYCINE

MSEVFEGYERQYCELSANLSRKCSSASLVSDQEQKPQKLSEIKAGLDDADVLIRKMDLEARSLQPSVKAMLLAKLRE
YKSDLTNLKKEFKRLTSPNADEAAREELLETGMTDTHLASADQRERLTMSVERLNQSSERIRDSHRTLLETEELGINII
QDLHSQRETLLNSHKRLHGIDDAIDKSKKVLTTMSRRITRNKWIVASVIGALVFAIVIILFYKLSH
>I1JE83|VTI12_SOYBN-GLYCINE
MSEVFEGYERQYCELSANLSRKCSSASLVSGQEQQQQKLSEIKAGLDDADVLIRKMDLEARSLQPSVKAMLLAKLR
EYKSDLTNLKKEFKRLTSPNADEVAREELLETGMANTHLASADQRERLTMSVERINQSGERIRESHRTLLETEELGVN
ILQDLHSQRETLLNSHKRLHGIDDAIDKSKKVLTTMSRRITRNKWIVASVIGALVFAIVIILFYKLSR
>U5G7Q6|VTI12_POPTR-POPULUS
MSEVFEGYERQYCELSANLSRKCNSTSLLPDGVEKNAKVNEIKSGLDDCDVLIRKMDLEARSLQPSVKAMFLAKLRE
YKSDLNKLKREFKRITSGDVSQASREELLEAGMADAHSVSTDQRERLTMSVERLNQSSDRIKESRRAMLETEELGVS
VLEDLHQQRQTLLHAHNKLHGVDDAIDKSKKVLSSMSRRMTRNKWIVGSVIAALVVAIIFIILFKTSYH
>B9HXP4|VTI12_POPTR-POPULUS
MSEVFEGYERQYCELSANLSRKCNSTSLLPDGVEKNAKVNEIKSGLDDCDVLIRKMDLEARSLQPNVKAMLLAKLRE
YKSDLNKLKREFKRITSGDVNQASRVELLEAGMADVHSVSADQRERMTMSVERLNQSGDRIKESRRTMLETEELG
VSILEDLHQQRQTLLHAHNKLHGVDDAIDKSKKVLTSMLRRMTRNKWIVGSVIAALVVAIIFIILFKISHH
>A5B0Y2|VTI12_VITVI-VITIS
MSEVFEGYERQYCELSTNLSRKCTSASVLDGEEKHQKISEIEAGLEEAVILIRKMDLEARSLQAGVKAMLLAKLREYK
ADLNKLKKEVKKITSPDTNQVAREELLESGMVDAHMVSADQKGRLAMSTEKLNESSDRIKESRRTMLETEDLGVSI
LQDLHQQRQTLLHAHEKLHGVDNAIDKSKKILTAMSKRMSRNKWIVGSVISALVLAIIFILYFKLS
>A1A6H8|VTI13_ARATH-ARABIDOPSIS
MEYGSFTVVLYAVDGYFLSTFCSFQVKKMDLEARNLPPNVKSSLLVKLREYKSDLNNFKTEVKRITSGNLNATARDE
LLEAGMADTLTASADQRSRLMMSTDHLGRTTDRIKDSRRTILETEELGVSILQDLHGQRQSLLRAHETLHGVDDNV
GKSKKILTTMTRRMNRNKWTIGAIITVLVLAIIFILYFKLTR
>Q9LVP9|VTI13_ARATH-ARABIDOPSIS
MSQGFERYERQYCEISANLSKKCTSAIALDGEQKKQNLSEIKSGVEEAEALVKKMDLEARNLPPNVKSSLLVKLREYK
SDLNNFKTEVKRITSGNLNATARDELLEAGMADTLTASADQRSRLMMSTDHLGRTTDRIKDSRRTILETEELGVSIL
QDLHGQRQSLLRAHETLHGVDDNVGKSKKILTTMTRRMNRNKWTIGAIITVLVLAIIFILYFKLTR
>Q944A9|NPSN11_ARATH-ARABIDOPSIS
MDPISAVSEELAEIEGQINDIFRALSNGFQKLEKIKDANRQSRQLEELTDKMRDCKSLIKDFDREIKSLESGNDASTNR
MLNDRRQSMVKELNSYVALKKKYSSNLASNNKRVDLFDGPGEEHMEENVLLASNMSNQELMDKGNSMMDDT
DQAIERGKKIVQETINVGTDTSAALKAQTEQMSRVVNELDSIHFSLKKASKLVKEIGRQVATDKCIMAFLFLIVIGVIA
IIIVKIVNPNNKDIRDIPGVGLAPPAMNRRLLWNHY >I1GX14|NPSN11_BRADI-BRACHYPODIUM
DISTACHYONMDLASVNEELAEIDGQIADIFRTLQNGFQKLDKIKDANRRSRQLEELTDKMRDCKRLIKDFERVVKD
EAGRTDQETAKALNDKKQSLIKELNSYVALKKQHASENKRVDLFDGPSVEDGFGEENAMLASNMSNQQLMDHG
GHLMDETDQALARSKQTVQETINVGTETAAALKAQTEQMSRVVNELDSIHFSLKKASKLVKEIGRQVATDRCIMAL
LFLIVIGVIAVIIVKIVNPHNKDIPDIPGLAPPVSRRLLSIVENK
>I1MV96|NPSN11_SOYBN-GLYCINE
MDQLSAISEDLAEIDGHIADNFRALSNGFQKLEKIKDSNRQSRQLEDLTEKLRECKRLIKEFDKEVKTLESSFDRETNK
MLNEKKQSMIKELNSYVALKKQYATNIENKRIELFEGPNEGYAEENGLLASSMTNEQLMDHGNRMMNETDQAIE
RGKKVVQDTINVGTDTAAALKAQTEQMSRVVNELDSIHFSIKKASKLVKEIGRQVATDKCIMALLFLIVIGVIAIIIVKL
VHPENKDIRDIPGLAPPVQNRRLLWSHS
>Q5Z9Q1|NPSN11_ORYSJ-ORYZA
MDLESVNPELAEIDGQIGDILRALQNGFQKLDKIKDANRRSRQLEELTDKMRDCKRLIKDFERVVKDMAGSTDPET
ARMLHDRKQSMIKELNSYVALKKQYASENKRVDLFDGPSVEDGFGEENVLLASNMTNQQLMDQGNQLMDETD
QAIARSKQTVQETINVGTETAAALKSQTEQMSRIVNELDSIHFSIKKASQMVKEIGRQVATDRCIMALLFLIVAGVIA
IIVVKIVNPQNKTIRDIPGLAPPVSRRLLSIVEDI
>B9N2U3|NPSN11_POPTR-POPULUS
MDPLSSISEELAEINGQIADIFRALSNGFQKLEKIKDVNRQSRQLEELTGKLRECKRLIKEFDREMKDMESRNDPDTN
KMLNEKKQSMIKELNSYVALKKQYATNLENNKRVDLFDGPNEELHDDNVLLASSMTNQQLVDHGNQMMDETD
QAIERGKKVVQDTINVGTETAAALKAQTEQMSRIVNELDSIHFSIKKASQLVKEIGRQVATDKCIMALLFLIVVGVIAI
IIVKLVNPSNKDIRDIPGLAPPAPSRRLLWIPNQETS

>C5Z1V3|NPSN11_SORBI-SORGHUM
MDLASVNEELAEIDGQIGDILRALQNGFQKLEKIKDANRRSRQLEELTDKMRDCKRLIKDFERVSKDEAGHTDPATA
KMLHDRKQSMIKELNSYVALKKQQASENKRIDLFDGPSVEDGFGEENVQLASNMTNQQLMDQGNQLMDETDQ
AIARSKQTVHETINVGTETAAALKAQTEQMSRVVNELDSIHFSIKKASQLVKEIGRQVATDRCIMAMLFLIVAGVIAII
IVKIVNPHNKDIRDIPGLAPPVSRRLLR
>D7TTK0|NPSN11_VITVI-VITIS
MSSLAGISEELAEIDGQISDIFRALSNGFQKLEKIKDTSRQSRQLEELTGKMRECKRLIKEFDREVKDLEIRNDPETNK
MLNEKKQSMIKELNSYVALKKQYATNLENKRIDLFDAPADDVGEENVLLASSMTNQQLMDNGNRMMDETDQVI
ERSKKVVQDTVNVGTETAAALKSQTEQMSRIVNELDSIHFSIKKASQLVKEIGRQVATDRCIMALLFILVIGVIAVIIVK
LVNPNNKDIRDVPGLAPPALTRKLLWHPN
>Q9LNH6|NPSN12_ARATH-ARABIDOPSIS
MASELPMSPHLEQIHGEIRDHFRALANGFQRLDKIKDSSRQSKQLEELAEKMRDCKRLVKEFDRELKDGEARNSPQ
VNKQLNDEKQSMIKELNSYVALRKTYLNTLGNKKVELFDTGAGVSGEPTAEENVQMASTMSNQELVDAGMKRM
DETDQAIERSKQVVHQTLEVGTQTASNLKGQTDQMGRVVNDLDTIQFSLKKASQLVKEIGRQVATDKCIMAFLFLI
VCGVIAIIIVKIVNPNNKDIRDIPGLAPPAQSRKLLYFRE
>I1GRW7|NPSN12_BRADI-BRACHYPODIUM
MAASDVPMSPELEQVDGEIQDIFRALQNGFQKIDKIKDSNRQSKQLEELTGKMRECKRLIKEFDRVLKVEEKKSTSE
VNKQLNDKKQFMIKELNSYVTLRKTYQSSLGNKRIELFDTGNDDQLAGVNVQMASEMSNQQLIDSGMKQMDET
DQALERSKMVVKQTVEVGAQTAATLTQQTDQIKRIGNELDSVHFSLKKASQMVKEIGRQVATDKCIMAFLFLIVCG
VIAIIVVKVVNPHNKNIPDIPGLAPPAQNRKLLSIEAYRML
>A8ITN9|NPSN12_CHLRE-CHLAMYDOMONAS
MAPKKREPAKDEEIAAPETIVFERSEQEEQCEKIFRELDKMFKKLAKINKPDKIHSSIRDITAKLKQAKELIKDFEREAR
ADGVPQNELAARKKALAAELNGFIALKKEFAQTEGTKADLLNGAAPEAEQALEGMSMQQLMKKGRTDIQDIDKT
LERSERIVEDTKAVGTQVAATLNDQTKKLEKIVDDLNEIEFTMKKASAVIRDITRGLLTDKCIAFLLLLTVVGVVVIIVLK
IINPNKKKIAQGASAALNQTINAINDTAAGSSAIGAVTGAVNTAINQTSNAVGSIGGRRSLLSRPVLTKALLHLMELN
ATL
>I1LYG4|NPSN12_SOYBN-GLYCINE
MASNLQMSPQVEQIHGEIRDNFRALANGFQKLDKIKDANRQSNQLEELTDKMRECKRLIKEFDREIKDEEGRNPPE
VNKQLNDEKQSMIKELNSYVALRKTYMNTIGNKKLELFDNGAGVSVPTAEENVQLASEMSNQELINAGTKTMDET
DQAIERSKQVVHQTIEVGTQTAATLKGQTEQMGRIVNELDSIQFSIKKASQLVKEIGRQVATDKCIMLFLFLIVCGVI
AIIVVKIVHPNNKDIRDIPGLAPPVPTRRLLYARSGDHLD
>Q8H5R6|NPSN12_ORYSJ-ORYZA
MASDVPMSPELEQVDGEIQDIFRALQNGFQKMDKIKDSNRQSKQLEDLTGKMRECKRLIKEFDRILKEDEKKNSAD
VNKQLNDKKQLMIKELNSYVTLRKTYQSSLGNKRIELFDTGNDQVAEDNTVQMASEMSNQQLMDAGRKQMDQ
TDQVIERSKKVVAQTVEVGSQTAAALSQQTEQMKRIGNELDSVHFSLKKASQMVKEIGRQVATDKCIMAFLFLIVC
GVIAIIVVKIVNPHNKNIRDIPGLAPPAQNRKLLSIESFGSL
>A9SQW5|NPSN12_PHYPA-PHYSCOMITRELLA
MASELPPQLVNIEKEVCDAFKLLSTGFQRLDKIKDAGRQTKQLEELTAKMREAKRLIKEFDREINEGSDRVLPETAKL
LNEKKQSLIKELNSYVALRKTYTSSIGSRQELLDGGLHAGAARGAHVRVASTMSNQELVEAGRKQMDETDQTIERS
KQVVEDTINIGVQSATTLKGQTEQLGRINNEMDTLQFSLKKASGLVKEIGRQMATDRCIMFFLFLVVAGIIAVVVVK
VVNSSNKHGQAPELPPPAARRRLLLTRI
>A9SL82|NPSN12_PHYPA-PHYSCOMITRELLA
MGPGLPPQLASIEKEARDIFKLLTTGFQKLDIVKDVDKQNKQLEDLTAKMREAKRLIKEFDKETKEAESTISSESVKTL
NEKKQALIKELNSFVALRKTYTSSIGNREEHLDGGLHARSARGSTQMDSQPMDEATKTREKSSKVLEHNIHIGTETA
TTSEDRIGQTVEEQPSKPNDQVGTTRFSMKDASFKEIVRKMATNRCIMVVALIILVAIIVIVIMTLVHPEGEVTRSTP
PPRRRRLLMTAMDFQC
>A9S9X3|NPSN12_PHYPA-PHYSCOMITRELLA
MRFWHAILKFYLLIVIFSEGFQRLDKIKDLEKQRKQLEELTGKMRGVKRILKDFDQGIKDDPTNLELNKTVAEKKKSLI
RELNTYIALRKTFSSTISSKAELFEGGSQAGTATNQAYRVASTMSNQELLQVGRKQMDEMDKSIERSKRVVEDTLHI
GTETAVTLKAQTEQLGRIVNELDTIQFSIKKAAQLVREVGKQVRI
>A9T174|NPSN12_PHYPA-PHYSCOMITRELLA

MALGIPPQLASIEKEVQGIFKFLATGFQRIDKIKDAGRQSKHLEELSARMRGAKSRIKEFDVEIKVEEGKINPEFNKLL
TEKKQFMITERNSYVAKRRTYTSLIGNRQESLDKDSHAGSAQDADVCVASTLSHQELMQPGRK
>A9TDW6|NPSN12_PHYPA-PHYSCOMITRELLA
MSSVGPRMNLSPGISTVSFATSAAFFMGSWSSHCTWSPSSTFVKKLLSFIHLAPSLPRMHSCDLVPQPLGSASGFSL
EGFVSGSCELRMEEVAAAYSYEVEKTRFALQFFDNRLKGRIRNWSYSPFGLNSGGISSGDAAMVSGLPSQVASIEKE
VREIFLILASGFQRTDKIKDVDKKNKQLEELTAQMRDAKRLIKGFDKVMKYEESLTNLEFNKMLNEKKQSLINELNSF
VALRKTYPSSIGRREELLGGGSYTASLRDVDVRLASSVANEDPTQTARQPMDGTDKTTESSCEVYHMSIGTETATTA
ELTTLKGEVNSFEVRFSFCFLVDFEKEQSSKTKDDVDTTRFSTKTGSLKELGCKLATNRFVMLILLLITISLIVTVIVKLVH
PEGGLPSSSPPARRRRLLMTELDLLKTISS
>B9HXA1|NPSN12_POPTR-POPULUS
MASELQMSPQLEQIHGEIKDNFRALSNGFQRLNNIKDSNRQSKQLEELTGRMKECKRLIKEFDREIKVEESKNPPEV
NKQLNDEKQSMIKELNSYVQLRKTYMNSLDNKRVELFDMGAGASEPMAEENVRMASAMSNQELVDAGMKTM
NETDQAIERSKQVVEQTIEVGTQTAGTLKGQTEQMGRIVNELDTIQFSIKKASQLVKEIGRQVATDKCIMLFLLLIVC
GVIAIIIVKIVNPSNKDIRDIPGLAPPAPSRRLL
>C5X3B5|NPSN12_SORBI-SORGHUM
MGSDVPMSPELEQVDGEIQDIFRALQNGFQKMDKIKDSSRQSKQLEDLTAKMRECKRLIKEFDRILKDEEKKNTPD
VNKQLNDKKQFMIKELNSYVTLRKTYQSSLGNKRIELFDTGNDQVADETPVQMASEMSNQELISAGRKQMDQTD
QAIERSKMVVAQTVEVGAQTAATLSQQTDQMKRIGNELDSVHFSLKKASQMVKEIGRQVATDKCIMAFLFLIVCG
VIAIIVVKIVNPHNKNIRDIPGLAPPAMNRKLLSVDAFGGLRAL
>D7T687|NPSN12_VITVI-VITIS
MATDLQMTPQLEQIHGEIRDNFRALANGFQKLDKIKDPNRQSKQLEELTGRMRECKRLIKEFDREMKDEERGNPP
EVNKQLNDEKQSMVKELNSYVALRKTFMSSLGNKRLELFDMGAGASDPTADDNVQVASSMSNQELIQAGNKTM
DETDQTIERSKQVVAQTIEVGTGTAVTLKGQTDQMGRIVNELDTIQFSIKKASQLVKEIGRQVATDKCIMLFLFLIVC
GVIAIIIVKIVNPNNKSIKDVPGLAPPAPSRRLLYLKASDYLE
>Q9LRP1|NPSN13_ARATH-ARABIDOPSIS
MASNLPMSPQLEQIHGEIRDHFRALANGFQRLDKIKDSTRQSKQLEELTDKMRECKRLVKEFDRELKDEEARNSPE
VNKQLNDEKQSMIKELNSYVALRKTYMSTLGNKKVELFDMGAGVSGEPTAEENVQVASSMSNQELVDAGMKR
MDETDQAIERSKQVVEQTLEVGTQTAANLKGQTDQMGRVVNHLDTIQFSIKKASQLVKEIGRQVATDKCIMGFLF
LIVCGVVAIIIVKIVNPNNKDIRDIPGLAPPAQSRKLLYLRNQDYM
>Q9SYG8|USE11_ARATH-ARABIDOPSIS
MGIGKTEINFMRLLSAAPNQQNQSKLMHYVATLREQLEQLSEEKTLEGLPRVTNAKVNEYYEKIEAVVSRIVAQVP
HTEVSDEAFAKDSTNDSSPKVEDDTRTPNSPQLRRRIVPASSKEQSYDADPSKPIKLDTAAQAQVNKQRKLQEDLT
DEMVVLARQLKERSQMISQSVQNTEKILDSTEEAIEQSLASTGHATVRATKIYSESSKTSCFQWLLILAMTCVFIMVV
MLIRVT
>Q6NKR3|USE12_ARATH-ARABIDOPSIS
MMGISKTEINLRRLLSAAPNQQNQSKLMHYVATLREQLEQLSEEKTPEGLPRVTKAKVNEYYEKIEAVASKIASQEP
ETEVSDEPFAKDSTSGSSPKIEDEPRSPTSPQLRRRIVPASSKEQSFDAADADSSKPIKLDTAAQAHIDKHRKLQEDLT
DEMVVLARQLKERSQAISQSVQNTEKILDSTEEAIEQSLASTGHATTRATKIYSQSSKTSCFQWLLIFAMICVFIMVV
LLIRVT
>I1I8Z1|USE12_BRADI-BRACHYPODIUM
MVFSKVEVNLRRLLEAAPRQQNQAKLVHYITTARELLEQLGTEITPEGVSSVSKAKLGEYSEKIEALAATLAATVPEN
ENPVEESIEEQSSYEREKVGSPISLSSGLRRRPTTQVEVGPSSYERKERDTGAPIKLDAEAQAHIEKHRKLQDDLTDE
MVDLARQLKESSLLMNQSVQDTEKILDSTERVVEHSLASTGRATARASEVYSLASKTTCFQWLLIFMMTCMFVMV
VLLIRVT
>I1LVK5|USE12_SOYBN-GLYCINE
MGISKTEVNLKRLLAAAPQQQNQAKLVHYVATLREQLEQLAEERAPEGLPRISKAVLNDYSEKIEAIASKLVNHVSD
PPVPKKDFERNSVEENSSEIEETKQILLSSGLRRRPVPASSTEERAHKPAETDHISPVKLDAAAHAHIEKHRMLQDNL
TDEMVVLAKQLKESSLMMSQSLQNTEKILDSTEKAIEHSLASTGRANVRATAIYSESSKTSCLTWLVMFVMTCVFV
MVILLIRVT
>I1M717|USE12_SOYBN-GLYCINE

MGISKTEVNLRRLLAAAPQQQNQAKLVHYVATLREQLEQLAEERTLEGLPRISKAMLNDYSEKIEAIASKLVNHVIDT
QVPGKDFERNFVKEKHSEIEEKKQILLSSGLRRRPVPASSTEDRAHEPAETDHTSPIKLDATAHAHIEKHRKLQEDLT
DEMVVLAKQLKESSLTMSQSLQNTEKILDSTEKAIEHSLASTGRANVRATAIYSESSKTSCLTWLVMFVMTCVFVM
VILLIRVT
>K7MK36|USE12_SOYBN-GLYCINE
MGISKTEVNLKRLLAAAPQQQNQAKLVHYVATLREQLEQLAEERTPEGLPRISKAVLNDYSEKIEAIASKLVNRVSDP
PVTKKDFERNSVKENSSETEETKQILLSSGLRRRPVPASSTEDRAHEPAETGLTSPVKLDATAHAHIEKHRKLQDDLT
DEMVVLAKQLKESSLMMSQSLQNTEKILDSTEKAIEHSLASTGRANVRATAIYSESSKTSCLTWLVMFVMTCVFVM
VILLIRVT
>Q6H4Y9|USE12_ORYSJ-ORYZA
MGISKTEVNLLRLLDSAPRQQNQAKLIHYVTTSRELLEKLAAENTSEGISSIAKGRLNEYSERIEELAARLASLVPGYEN
AVEAIRKEESYLEGEQIRSPIALSPGLRRRLTALQEIEQPTNAKERNVAEPLRLDEAAQANIEKYRNLQEDLTDEIVELA
RQLKDSSLMMNQSVQATEKILDSTERAVAYSLAGTDRANAQAVEVSSLTSKTTCFQWFLLFVMTCMFIMVVLLIR
VT
>Q6K2Z1|USE12_ORYSJ-ORYZA
MVLSKVEVNLIRLLEAAPRQQNQTKLVHYVTTARELLEQLGSETTPEGISSVSKAKTSEYSEKIEALAARLAAPEPENE
MPVDENREVESSYEGDKPGSPISLSSGLRRRPM
>Q6YZG2|USE12_ORYSJ-ORYZA
MFCENQIILQGICLFISVSLHRDLSEGFRERHIDILNLPKGLTPRSVHVLILWPRFLTFAGTGPENEMPVDENREVESS
YEGDKPGSPISLSSGLRRRPIAHNVVGPSSHERKDRDIGAPIKLDEEAQAHIEKHRKLQEDLTDEMVELARQLKESSL
TMHQSVQETEKILDSTERAVEHSLASTGRATTRAAEVYSLASKTTCFQWLLIFMMTCMFIMVVLLIRVT
>Q6YZC6|USE12_ORYSJ-ORYZA
MALSKVEVNLLRLLEAAPRQQNQAKLVHYVTTARELLEQLGAETTPEGISSVSKAKISEYSEKIEALAARLAVPEPENE
VPVDENREVESSYEGEKPGSPISLSSGLRRRPIAHTDVGPSSHARKDRDIGAPIKLDAEAQAHIEKHRKLQEDLTDEM
VELARQLKESSLTMHQSVQETEKILDSTERAVEHSLASTGRAATRAAEVYSLASKTTCFQWLLIFMMTCMFIMVVLL
IRVT
>A9RSU7|USE12_PHYPA-PHYSCOMITRELLA
MGLSKAEVNLKRLLRAQVNQANEAKLVHYVATMRTLLAELIGDPARQELATIPASRAREYAEQIELVAQKIRDCKFA
DNDRDQAAIRDSDASQGPEMSVSGMRRRLRGDELGNLGARESSRPPARVDASLQSLMQRHRELQEGLTDEMV
MLSAQLKESSLMMDQALHETDKVLESTEDAVEYSLASTNRANVRMGALSSQSWKTSCLTWLILFVVFGMFMFM
VVLIRVT
>B9IN18|USE12_POPTR-POPULUS
MGISKTEINLRRLLSSAPQQQNQAKLVHYIATLREQLEQLAEERTADELPRVSKAVLNDYSEKIEAIASKLVNSLPEIEA
PQETFAGDSGKESPKADGGDQIAPSPGLRRRFAPVSNFEDKTRDSIKADASAPVKLDAAAQAHIEKHRKLQDDLTD
EMVGLAQQLKESSLLMSQSLQNTEKILDSTEQAVEQSLASTGHANVRTMDIYSKTSKTTCFTWLLMFLMTCIFIMV
VLLIRVT
>C5YHR0|USE12_SORBI-SORGHUM
MRLNKVEVNLIRLLEAAPRQQNQAKLVYYVTTARELLEQLGAETTPEGISSISKAKLSEYSEKIEALASRLAASVPENEK
PIIESRDEISYEEAKPESPISLSSGLRRRSTAHAEVGPSHQEGKGDIGAPIKLDAEAQAHIEKHRKLQEDLTDEMVELA
RQLKESSLAMNQSVQETEKILDSTERAVEQSLASTGHATSRAAEVYSLASKTTCFQWLLMFVMTCMFVMVVLLIRI
T
>D7TIM5|USE12_VITVI-VITIS
MPFNHALIDSFCIPLKASKKSIRKRTYRSLGFQGRPNRSHRRDPTGQRPRRSSTVVQDLVMGISRTEVNLRRLLAAA
PQQQNRAKLIHYVATLREQLEQLTEERTLEGLPRVSKQKVNEYAEEIEAIASKIIAPSSDVQVSQEPLSGTYGQESHSK
DEEDSIPHSPGLRRRFVPASNVEDRTHDTVKADASGPVKLDAAAQAHVEKHRKLQEDLTDEMVGLAKQLKQGSLL
MSQSIKNSEKILDSTEKAVEHSLASTGRVNTRAMEINSNGFKTTCFTWILIFVMTCIFIMVVLLIRVT
>D7SQE4|USE12_VITVI-VITIS
MGISRTEVNLRRLLAAAPQQQNQAKLIHYVATLREQLEQLTEERTLEGLPRVSKPKVNEYSEKIEAIASKIGSPSSNV
QVSQEPFSGTSGKEGTSKTEEEDHMPLSPGLRRRFVPASKFEDRSHDTIKADASGPVKLDAAAQAHIEKHRKLQED
LTDEMVGLAKQLKQSSLLMSQSIQNTEKILDSTEKAVEHSLASTGHVNTRAMDIYSNSFKTTCFTWIMIFVMTCIFI
MVVLLIRVT

>D7U927|USE12_VITVI-VITIS
MGISRTEVNLRRLLAAAPQQQNQAKLIHYVATLREQLEQLSEERTLEGLPRVSKQKVNEYAEEIEAMASKIIAPSSDV
QVSQEPLSGTYGQEGHSKEGEDSIPHSPGLRRRFVPASNVEDRTHDTVKADTLGPVKLDVAAQAHIEKHRKLQEDL
TDEMVGLAKQLKQGSLLMSQSIENSEKILDSTEKAVEHSLASTGRVNTRAMEINSNGFKTTCFTWILIFVMMCIFIM
VVLLIRVT
>Q9M2J9|BS14A_ARATH-ARABIDOPSIS
MNPRREPRGGRSSLFDGIEEGGIRAASSYSHEINEHENERALEGLQDRVILLKRLSGDINEEVDTHNRMLDRMGND
MDSSRGFLSGTMDRFKTVFETKSSRRMLTLVASFVGLFLVIYYLTR
>I1I026|BS14A_BRADI-BRACHYPODIUM
MNSRRDFRSHRAALFDGIEEGGIRGSAYSSREIHEHENDQAVDNLHERVSILKRLTGDIHDEVENHNRMLDRMGN
DMDTSRGFLSGTVDKFKMVFETKSSRRMATMVASFVAAFLLLYYLTR
>A8IWY7|BS14A_CHLRE-CHLAMYDOMONAS
MLGTSRDRRGLSRDKVDIESMEQENDRELDHLADRVALLKNVTHGINKEVNDQHKLLENMDGSFASVGGLMSA
VTDKFRAVYNDKANKRLIYGAVAVAAGLFLVWYFMLR
>I1JP17|BS14A_SOYBN-GLYCINE
MNARRDNRNNRVSLFDGIEEGGIRASSVYSSSHEIDEHDNEQALDGLQDRVNLLKRLSGDINEEVDSHNRMLDR
MGNDMDSSRGVLSGTMDKFKMVFETKSNQRMFTLVASFVVLFLIIYYLTRY
>C6SZI2|BS14A_SOYBN-GLYCINE
MNARRDGRNNRVALFDGIEEGGIRASSLYSSSSSHEIDEHDNEQALDGLQDRVNLLKRLSGDINEEVDSHNRMLD
RMGNDMDSSRGVLSGTMDKFKMVFETKSSRRMFSLVASFVVLFLIIYYLTR
>Q6ZBW9|BS14A_ORYSJ-ORYZA
MNSRRDFRSHRAALFDGIEEGAIRSSAYSSQIHEHENDQAMDSLHDRVSVLKRLTGDIHEEVENHNRMLDRMGN
DMDASRGFLSGTVDKFKMVFETKASRRMATMVASFISVFFLIYYLTK
>A9TP30|BS14A_PHYPA-PHYSCOMITRELLA
MNTRRDYRATRSQLFDSLEDGGLRSSAPYASTAEIAEQENDRSLGELHDRVNILKRLTGDIHEEVESHNKLLEGMG
NAMDVSRSLMAGTMDRFTRAFETKSSRNLATIVVSCVVIFLLVYYLTKS
>A9TX52|BS14A_PHYPA-PHYSCOMITRELLA
MNPRRDYRGTRSQLLEMEEGGLRTSAPYASSAEIAEQENERSLGELEDRVKLFKRLAGDVQDEVDSHNKLLEGMG
NAMDASRSMMAGTMARFTRVFETKSSRNIATIVASCVVIFLLVYYLAR
>B9GS38|BS14A_POPTR-POPULUS
MNSRRDVRNNRAALFDGIEEGGIRASSSYSSHEIDEQDNERALEGLEDRVSLLKRLSGDINEEVDSHNLMLDRMGN
DMDSSRGVLSGTMDRFKMVFETKSSRRMLTLVASFVVIFLIIYYLTR
>B9H8J7|BS14A_POPTR-POPULUS
MNSRRDIRNNRAALFDGIEEGGIRASSSYSSHEIDEQDNERALEGLQDRVILLKRLSGDINEEVDNHNLMLDRMGN
DMDSSRGVLSGTMDRFKMVFETKSSRRMFTLVASFVVLFLIIYYLTR
>D7TSA1|BS14A_VITVI-VITIS
MNSRRDHRGNRAATFDGIEEGGIRASSSYSHEIDEVDNETAVDGLEDRVVMLKRLTGDIHEEVESHNRMLDRVGN
EMDASRGILSGTMERFKMVFETKSSRRMFTLIASFVVIFLVIYYLTR
>D7U2W9|BS14A_VITVI-VITIS
MNSRRDYRGNRIALFDGIEEGGIRASSSYSSHEIDEHDNERAVDGLQDRVNLLKRLTGDIHEEVESHNRMLDRVGN
DMDASRGILSGTMDRFKMVFETRSSRRMFTLVASFVVIFLIIYYLTR
>Q94CG2|BS14B_ARATH-ARABIDOPSIS
MNFRRENRASRTSLFDGLDGLEEGRLRASSSYAHDERDNDEALENLQDRVSFLKRVTGDIHEEVENHNRLLDKVG
NKMDSARGIMSGTINRFKLVFEKKSNRKSCKLIAYFVLLFLIMYYLIRLLNYIKG
>K7LGR9|BS14B_SOYBN-GLYCINE
MSYRRDNRSSRSSLVDGFDSLEEGGLRASSSYSREINEHDNDKAIENLQDRVSFLKRLTGDIHEEVESHNQLLDRVG
NKMDGSRGVMMGTMDRFKKVFEKKSARKTCSLVGYFTLAFIFIYYLIRMLGYFTLG
>I1NEQ6|BS14B_SOYBN-GLYCINE
MSYRRDNRSSRSSLVDGFDSLEEGGLRASSSYSREINEHDNDKAIESLEDRVSFLKRLTGDIHEEVESHNQLLDRVGI
KMDGSRGMMMGTMDRFKNVFEKKSARKTCSLVVYFTLAFIFIYYLIRMLGYFTLG
>Q8VXX9|BETL1|SFT11_ARATH-ARABIDOPSIS

MASNPHRSGAGGSLYGGAAPYRSREGLSTRNAAGSEEIQLRIDPMHSDLDDEITGLHGQVRQLKNIAQEIGSEAKF
QRDFLDELQMTLIRAQAGVKNNIRKLNMSIIRSGNNHIMHVVLFALLVFFVLYIWSKMFKR
>Q8L9S0|BETL2|SFT11_ARATH-ARABIDOPSIS
MASNRGAGGSLYGGADPYRSREGLSTRNASGSEEIQLRIDPMHSDLDDEILGLHGQVRQLKNIAQEIGSEAKSQRD
FLDELQMTLIRAQAGVKNNIRKLNLSIIRSGNNHIMHVVLFALLLFFILYMWSKMFKR
>I1GT19|SFT11_BRADI-BRACHYPODIUM
MANPMYGSGPLRSRNASSSDEIQLRIDPVHGDLDEEIDGLHSRVRLLKGVAQEINSEAKFQNDFLNELQMTLMKA
QAGVKNNMRRLNKSIIQQGSNHIVHVVLFALLCFFVVYFLSKFSRR
>A8J818|SFT11_CHLRE-CHLAMYDOMONAS
MAGYPSRGGLSSRQQDQVTINVGKFDFDSEVEGLRGHVKKIKQLSLAIEDERKEQGEIINSLEDTMERAKLVMRRA
MGRLNIAARQARSNHMLYLVLFAVAMFTVLYVLGKVYRIGRAVLGG
>C6T1H3|SFT11_SOYBN-GLYCINE
MVANSHRVGSSYGGAAPYRSRDGLSPRPVGASEEIQLRIDPLDLDDEITGLHRQVRRLKHVAEEIGTEVKYQKNFLE
ELQMTMIKAQAGVKNNLRRLNKSIIQSGSNHIIHVILFALVCFFVVYLWSKMIRK
>I1K9T5|SFT11_SOYBN-GLYCINE
MPPLIDPGLFVFYSLFPFLLFNSISCVNCREGLSTRPVASSDEIQLHIDPGIDFDDEITGLRGQVKKLKNVAEEIGSEVK
FQRDFLEQVQMVMIQAQAGVKNNLRRLNKSIVKNGSNNIVHVIAFALVCFFIVYFWSKMSRK
>C6T099|SFT11_SOYBN-GLYCINE
MAANSHRLGSSYGGAAPYRSRDGLSTRPVGASEEIQLRIDPLDLDDEITGLHRQVRRLKHVAEEIGTEVKYQKTFLEE
LQMTMIKAQAGVKNNLRRLNKSIVQSGSNHIIHVIIFALVCFFVVYLWSKMFRK
>Q6ZL92|SFT11_ORYSJ-ORYZA
MANPLHGSGPLRSRNASSSDEIQLRIDPVHGDLDEEIDGLHSRVRLLKGVAQEINAEAKFQNDFLSQLQMTLIKAQ
AGVKHNMRRMNKSIIQQGSNHVVHVVLFALFCFFVVYLLSKFSRR
>A9SK71|SFT11_PHYPA-PHYSCOMITRELLA
MAWTDDSLGRRRNPNYSYNSDQVQLRVDPRDQLDEEIYGLRNKVAQLKQVAQHIDTETKYQNELLNQLEETVAK
GQAILKITMKRLNRTLNQRGISPLYLAIVFALLCFFSVYLYIKFHRRT
>C5XCF2|SFT11_SORBI-SORGHUM
MAHPMYGSGPLRSRNAANSDEIQLRIDPVHGDLDEEIDGLHSRVRMLKGVAQEINSEAKFQNDFLHELQMTLAK
AQAGVKNNMRRLNKSIIQQGSNHVLHVVLFALFCFFVVYLLSKFSRR
>D7SST6|SFT11_VITVI-VITIS
MANLYRSREGLSARPVANSDEIQLRIDPVHADLDEEITGLHKKVTMLKTVAQEIESEAKFQNDYISQLQMALSKTQA
GLKNNMKRLNRAMAQKGSNHVLHVILFGLACFSVVYLWSKHLRR
>E0CRS3|SFT11_VITVI-VITIS
MASTSHRGAPYYGADPYRSREGLTTRAAAGSDEIQLRIDPLNADLDDEITGLRSQIRQLKGVAQEIESEATFQNDLIN
KLQMTLVRAEAGVKNNLRQLKRSIIQEGSSHVIHVVLFALLCFTVIYLWSKISRR
>Q9SA23|SYP51_ARATH-ARABIDOPSIS
MASSSDSWMRAYNEALKLSEEINGMISERSSSAVTGPDAQRRASAIRRKITIFGNKLDSLQSLLAEIHGKPISEKEMN
RRKDMVGNLRSKANQMANALNMSNFANRDSLLGPDIKPDDSMSRVTGMDNQGIVGYQRQVMREQDEGLEQL
EGTVMSTKHIALAVSEELDLQTRLIDDLDYHVDVTDSRLRRVQKSLAVMNKNMRSGCSCMSMLLSVLGIVGLAVVI
WMLVKYM
>F4I2U7|SYP51_ARATH-ARABIDOPSIS
MILERSSLAETSSYARGHASYMRRKITILANGVQTLKNLLAESQGKSISAKEMSRCKDMVEDLRSKAYQMASALDM
LKFSNIGSLLGQDDIMSRVIDMDNQEIVGFQRTTMKVQDKALEMLEKGVMHLKREALAMNMELGLQTRLIDRLD
HHVDVSASDVEELQRSIDTGVCMTLLLSVVVVVFGLSEFVKFGRRRILG
>F4I2U8|SYP51_ARATH-ARABIDOPSIS
MASLCDSWIREQNETLKLSEEIDGMILERSSLAETSSYALRHASSMRRKITILATRVQTLKYLLAESQGKSISGKEMSR
RKGTFENLRSKANQMASALDMLKFSNIDILLRPEKDDIMSRVIGLDNQGIVGLHRQVMKEHDEALDMLEETVMR
VKHNALVMNEQIGLQTRLIDGLDHHVDVSDSGVRVIHIRA
>I1M6L1|SYP51_SOYBN-GLYCINE
MASSSDSWVKEYNEALKLADDISGMISEQSSFPASGPETQHHSSAIRRKITILGTRLDSLQSLLSKLPGKQPISEKEM
NRRKDMLSNLRSKVNQMASTLNMSNFANRDSLLGPERKPDAMTRMVGLDNNGLVGLQRQIMKEQDDGLEQLE

ETVASTKHIALAVNEELDLHTRLIDDLDQHVDVTDSRLRRVQKNLAVLNKRTKGGCSCMCMLLSVVGIVALIVVIWL
LVKYL
>F6HD99|SYP51_VITVI-VITIS
MNRRKDMLSNLRSKVSQMASTLNMSNFANRDSLLGPEIKPADAMSRTTGLDNQGLVGLQRQIMKEQDEGLDNL
EETVISTKHIALAVNEELDLHTRLIDTLDQHVDTTDSRLGRVQKNLAILNKRTKGGCTCFCMLLLVIGIVILIVVILVLIKY
L
>Q94KK7|SYP52_ARATH-ARABIDOPSIS
MASSSDPWMREYNEALKLSEDINGMMSERNASGLTGPDAQRRASAIRRKITILGTRLDSLQSLLVKVPGKQHVSEK
EMNRRKDMVGNLRSKTNQVASALNMSNFANRDSLFGTDLKPDDAINRVSGMDNQGIVVFQRQVMREQDEGL
EKLEETVMSTKHIALAVNEELTLQTRLIDDLDYDVDITDSRLRRVQKSLALMNKSMKSGCSCMSMLLSVLGIVGLAL
VIWLLVKYL
>Q946Y7|SYP61_ARATH-ARABIDOPSIS
MSSAQDPFYIVKEEIQDSIDKLQSTFHKWERISPDMGDQAHVAKELVATCGSIEWQVDELEKAITVAAKDPSWYGI
DEAELEKRRRWTSNARTQVRNVKSGVLAGKVSSGAGHASEVRRELMRMPNSGEASRYDQYGGRDDDGFVQSES
DRQMLLIKQQDEELDELSKSVQRIGGVGLTIHDELVAQERIIDELDTEMDSTKNRLEFVQKKVGMVMKKAGAKGQ
MMMICFLLVLFIILFVLVFLT
>I1HVG3|SYP61_BRADI-BRACHYPODIUM
MSSAQDPFYIVREEIQGSIGKLQTTFQRWEQIASNTGEYVHLTKELLTSCESIEWQVDELEKTISVASRDPAYYGLDE
VELSRRRNWTGSARSQVGAVRRAVEKGKNNSAMARHQDLMGTSRNHYSSQDNDDYIASESDRQLLLMRQQDD
ELDELSASVQRIGGVGLTIHEELSGQERILNDLSLEMETTSNRLDFVQKKVAVVMKKAGIKGQIMLILFLVILLIILFVLV
FLT
>A8JFM0|SYP61_CHLRE-CHLAMYDOMONAS
MNFKQHYLRTQDNKVISYFEGHRLADMQFSEPFNIIRADIDEQVRVLAKGSGELTAAVWAESADVARLAGNLGYD
CDALLAELGEVERAMDIVAAEPQRFHVSPAEVQARRSWISTTRDAVRRTAENVAPHRPPPVPRQQPVAIGVPVTV
AGPLTCTFISPEPYSAASDPCMTHRSSPPLSGSMRVASAIAGAAHANAAYAASATASAAVSTITPVRGAAAAARTA
ASSSASAAHRAASAAVGAGHTAAAAGTGAVLGGAGGFISGQLQAQQRAIRRQDGLLDDLSASLDRIADQGRRIG
RELEEQTGALSQLEAGVDAAVGGLRGAHRRALGLLRGR
>Q8S4W5|SYP61_CHLRE-CHLAMYDOMONAS
MPSNDPFYLIRQEIQDSVNELQQRMSRFHGLTATNPERKKIAQTVEEGCGSLSWQLNELDTAVDRASENPQRFNL
TPEELSSRRRWITNTRRQLDGMKDTLRTATAPAPAVSAAESKAIAQNDKFLTGQYESQQLVMKRQDQDLEDIEQA
VIRIGRQGREIGNELAEQERMLDELDQDVDTTHSRLKAAQKKMQELIRKSGSNTQLVLIVVLIVILVLLATFAFM
>C6TNI1|SYP61_SOYBN-GLYCINE
MPSAQDPFYVVKQEIQESIDKLKSTFHQWENTADVGERSSLSKEVLASCESIAWQVDELDKAISVAARDPSWYGID
EVEVENRRKWTSDARTQVNKAKRTVEAGKGSNNASLSGMHRELMRLPSSHQTTSNQYAAQDNDDFIESESDRQ
MLLIKRQDEELDELSLSVQRIGGVGLTIHEELLAQEKIIDELGNEMDGTSNRLDFVQKKVAMVMKKASAKGQIMMI
LGLLAMFIFLFILVFFT
>C6TJZ6|SYP61_SOYBN-GLYCINE
MPSAQDPFYVVKAEIQDSIDKLQSTFHQWESKSGAAEQGHLTKEVLAGCESIEWQVDELDKAIAVASRDPSWYGI
DEAEVESRRRWASSARSQVGTMKKAMESGKGSSTTSHASVNGMRRELMRLPNSHQTDSSNQYAARDNDDFIQS
ESDRQTLLIKRQDEELDELSESVRRIGGVGLTIHDELTAQEKILDELGSEMDSTTNRLDFVQKKVAMVMKKASAKGQ
IMMILGLLALFIFLFILVFFT
>I1M6Q8|SYP61_SOYBN-GLYCINE
MPSAQDPFYVVKQEIQESIDKLQSTFHQWEKTADAGERSNLSKEVLGSCESIEWQVDELDKAISVASRDPSWYGID
EVEVENRRKWTSDARTQVSTAKKAVQAGKGLNNASLNGMHKELMRLPSSHQTTSNQYAAQDNDDFIESESDRQ
MLLIKRQDEELDELSLSVQRIGGVGLTIHEELLAQEKIIDELGNEMDSTSNRLDFVQKKVAMVMKKASAKGQIMMI
LGLLALFIFLFILVFFT
>Q5JMS0|SYP61_ORYSJ-ORYZA
MSSAQDPFYIVREEIQDSIDKLQTTFHRWEKTPSNTGEHVHLTKELHTSCESIEWQVDELEKTILVASRDPAYYGLDE
VELSRRRNWIGSARNQVAAVRRSVEKGKSNSTFSAHQDMGTSRSNHYTAQDNDDFIASESDRQLLLMRQQDEEL
DELSESVQRIGGVGLTIHEELSGQERILNDLSLEMETTSNRLDFVQKRVAMVMKKAGIKGQIMLILFLVVLFIILFVLV
FLT

>A9SMJ2|SYP61_PHYPA-PHYSCOMITRELLA
MNGKELCQEDKSIRPHSSPSYSVSNGHSNSQGFEIENYALLFKEQDEDLEGLRESVERLGNMGKSINEEISIQERLIG
DVEHNIDTTTTRLHFVQKKMEIMIVKAGSNGQIFMIVFLIVLLIVLVVLIFYT
>A9S878|SYP61_PHYPA-PHYSCOMITRELLA
MSASDPFYLVKDEIQDTVTKLLSTLVRWEKLPTSSTERSVIGGEMLSSCESLEWQVDELDKATSVAEKDPARFKLDA
VEIKRRKSWTSSTRNQVHSITEKLQSKSLTNAGGADAPTSRPGFLRIDDQFQQAPTRSNYDSHIANESDRQELLLRE
QDEGLDDLSASLTHVGHVGVSIHEELSLQGQLMEKFSEDTDGTASRLDVVQKKLATVMKMAGWKGQVFMIVFLV
VLLVILIFLVFSG
>A9SDQ0|SYP61_PHYPA-PHYSCOMITRELLA
MSALDPFYLVREEVQDSVVKLQVTLGRWEQLPSSAAERIVLHKELLSGCESIEWQVVNELDRAIGVAERDPARFSV
DSAEIERRKKWTASTRSQVTTVLSVVVEKNTEASNGQISRRELMRLENQYQPTSNHGVDDVYESDRQALILKEQDE
DLDDLSATVERLGDVGLSIHEELSVQGHLMDELTNDMDSTANRLDFVQKRIAGVLKKAGWKGQVMTIVFLVVLLLI
LTLLVFSG
>B9H2P0|SYP61_POPTR-POPULUS
MSSAQDPFYIVKEEIQESIDKLQSSFHQWERISSDSGEQVRLTKELLAACESIEWKVDELDKAISVAARDPSWYGIDE
AELEKRRRWTSTARTQVGNVKKAVVAGKELNLSGTASVNGMRRELMRMPDAQQTDKSNQYTQDNDDFIQSES
DRQMLLIKQQDDELDELSASVERIGGVGLTIHEELLAQERIIGDLDTEMDTTSNRLDFVQKKVAMVMKKASAKGQL
MMILFLVVLFIILFVLVFLT
>C5XIP8|SYP61_SORBI-SORGHUM
MSSAQDPFYIVREEIQDSIDKLQSTFHRWEQTASNTGEYVHLTKELLTSCESIEWQVDELEKTISVASRDPAYYGLDE
VELSRRRNWTGSARKQVGTVKRAIEKGKSNAATSKYQDTSRTNHYSAQDNDDFISSESDRQLLLMRQQDEELDEL
SESVQRIGGVGLTIHEELSGQERILNDLSLEMETTSNRLDFVQKRVAMVMKKAGIKGQIMLIAFLVVLFIILFVSVFLT
>F6HB30|SYP61_VITVI-VITIS
MHTTNTISILFYVIVINSERPINLRLSTSRSVDHSGEGNVIDCKMSSAQDPFYIVKEEIQESIDKLLSTFHQWERIPVET
GEQVHLTKELLASCESIEWQVDELDKTISVAAKDPAWYGIDEVELDKRRRWTSTAHTQVSNVKKSVVAGKESNGT
GTANVNGMRREMMRMSNPHQADRSNQYGSQDNDDFISSESDRQLLLIRRQDEELDVLSASVERIGGVGLTIHEE
LLAQEKIIDELGSEMDSTSNRLDFVQKKVAMVMKKASAKGQIMMILFLVVLFIVLFVLVFLT
>Q9SF29|SYP71_ARATH-ARABIDOPSIS
MTVIDILTRVDSICKKYDKYDVDKQREANISGDDAFARLYGAFETQIETALEKAELVTKEKNRAAAVAMNAEIRRTK
ARLSEEVPKLQRLAVKRVKGLTTEELAARNDLVLALPARIEAIPDGTAGGPKSTSAWTPSSTTSRPDIKFDSDGRFDD
DYFQESNESSQFRQEYEMRKIKQEQGLDMISEGLDALKNMASDMNEELDRQVPLMDEIDTKVDRATSDLKNTNV
RLKDTVNQLRSSRNFCIDIVLLCIVLGIAAYLYNVLK
>I1H9N0|SYP71_BRADI-BRACHYPODIUM
MTVIDILTRVDSICQKYDKYDAEKLNGANVAGEDPFARLYGSVDAYISQCVEKAELAKQEKNRAAVVALNAEIRRTK
AKLLEEDLPKLQRLALKKVKGLTREELATRTDLVAALPDRIQSIPDGSATATKKNGTWGGASGSRTGGGIKFDSTSD
GNFDDEYFKGTEESNKFRQEYEMRRMKQDEGLDVIGEGLATLKNMASDMNEELDRQVPLMDEMDHKVDIANA
DLKNTNVRLKQTILQMRSSRNFCVDIVLLCVILGIAAYLYNVLKK
>I1HGP7|SYP71_BRADI-BRACHYPODIUM
MTVIDILTRVDAICQKYDKYDVDKLNGANVAGEDPFARLYGSVDAEISQCVEKAELAKQEKNRAAVVALNAEIRRT
KAKLLEEDLPKLQRLALKKVKGLTREELATRTDLVTALPDRIQSIPDGSATATKKNGTWGGASGSRTGGGIKFDSTSD
GNFDDEYFKGTEESNKFRQEYEMRRMKQDEGLDVIGEGLATLKNMASDMNEELDRQVPLMDEMDDKVDRANA
DLKNTNVRLKQTILQMRSSRNFCIDIILLCVILGIAAYLYNVLKK
>A8I202|SYP71_CHLRE-CHLAMYDOMONAS
MSIYDLIQRTNIILKKYEKYDAPIKTRGNKTDDPFMEEYQEVEAELEKLLEAASDVALEQNRALVAAKNAEIRRAKNV
LLTEAVQALEKKVKKGKGLNKHIIADRQQKIKELIEKVYAVPDGMSMAASRRPTRNYQKGKKAEAVYINGEFESNP
ANQEGYYSHTEATQAFEKEWDEAKKQQDKRLERIEAGVVELGDMARNIGEEVDRQNPIIDDIEQQMDKVTNNLK
TNNQKLQGVLKNMRSSRNFCVDIILITVLLAIGAYIYAMFM
>A8I205|SYP71_CHLRE-CHLAMYDOMONAS
MASIYDLIQRTNIILQKYERYDAPIKTRGNKTDDPFMDEYQEVEAELEKLLEAASDVALEQNRALVAAKNAELRRAK
NVLLTDGIQALEKKVKKGKGLNKHIIADRQEKIKELIERIYAVPDGMSMAGARRPARAYVKGKKGDPIYINGEFESNA

ASQDGYYAHTEATQAFEKEWDDAKRKQDERLDRIHEGVTQLGEIAVTLGEEVNRQAPIIDDIEKQMDKVTGTLKT
NNQKLAGVLKNMRSSRNFCVDIILITVLLAIGAYIYSMFM
>C6TKX1|SYP71_SOYBN-GLYCINE
MSVIDILTRVDSICKKYDKYDVQSQRDSNLSSDDAFAKLYASVDADIEALLQKADTASKEKSKASTVAINAEIRRTKAR
LLEEVPKLQRLAMKKVKGLSSQEFAARNDLALALPDRIQAIPDGTPAASKQSGSWAASASRPGIKFDSDGKFDDEYF
QQTEESSGFRKEYEMRKMKQDQGLDMIAEGLDTLKNMAHDMNEELDRQVPLMDEIDTKVDRASSDLKNTNVRL
RDTVNQLRSSRNFCIDIVLLIIILGIAAYLYNVLKK
>I1L8U0|SYP71_SOYBN-GLYCINE
MSVIDILTRVDSICKKYDKYDVDKHRDANVSGDDAFARLYASVDADIEALLQKAESASKEKGKASAVAINAEIRRTKA
RLLEEVPKLQRLAVKKVKGLSSQEFAARNDLVLALPDRIQAIPDGAPPVPKQTGGWAASASRPEIKFDSDGRFDDEY
FQQSEQSNQFRQEYEMRRMKQDQGLDVIAEGLDTLKNMAHDMNEELDRQVPLMDEIDTKVDKASSDLKNTNV
RLKDTVNQLRSSRNFCIDIVLLIIILGIAAYLYNVLKK
>K7LVG0|SYP71_SOYBN-GLYCINE
MSVIDILFRVDDICQKYDKYDIDKQRELNAYGDDLFARLYAAVESSIQSALNKSEVASTEKNRASAAALNAEVRRTK
GRLMDELPKLRKLVHKKVKGLTKEDMAIRQDLVLALPERIQAIPDGISGAAVQTAGWTATSSQPHIKFDSSEGHLDS
DYFQQSEESSQFRQEYEMRRTKQDEGLDIISEGLETLKDLAQDMNEEIDRQVPLMDEIDRKVDRAAADVRNTNVR
LKKTLTEIRSSRNFCIDIVLLCVLLGIVLYLYNALR
>I1NA77|SYP71_SOYBN-GLYCINE
MSVIDILTRVDSICKKYDKYDVEKQRDSNLSADDAFAKLYASVDADIEALLQKAETADKEKSKASTVAINAEIRRTKAR
LLEEVPKLQRLAMKKVKGLSSQEFAARNDLALALPDRIQAIPDGAPAAPKQTGSWAASASRPGIKFDSDGKFDDEY
FQQTEESSRFRQEYEMRKMKQDQGLDMIAEGLDTLKNMAHDMNEELDRQVPLMDEIDTKVDRASSDLKNTNV
RLRDTVNQLRSSRNFCIDIVLLIIILGIAAYLYNVLKK
>Q6AUC0|SYP71_ORYSJ-ORYZA
MSVIDILTRVDSICKKYDKYDVERLNGANVAGEDPFARLYGSIDADINECVEKAEAAKQEKNRATVVALNAEIRRTK
AKLVEEDLPKLQRLALKKVKGLTKEELATRSDLVAALPDRIQSIPDGSSSAKKNGTWGASGSRTGGAIKFDTSDGNF
DDEYFKGTEESNQFRREYEMRKMKQDEGLDIIGEGLETLKNMASDMNEELDRQVPLMDEMDEKVDRANTDLKN
TNVRLKETVLQLRSSRNFCIDIVLLCVILGIAAYLYNVLKKVRPDCAMDLFSTSSGDGWMSFDVSTSCSLYFYLICVIVV
WCSSGTCEHPC
>A9SNI4|SYP71_PHYPA-PHYSCOMITRELLA
MSLIDILARVDVLCKKYEKYDVDKQGGADSVSGQDQFAKLYAVVEADIEATLQKAEAAKNEKNRAAVATLNSEVRR
SKAALRAELPKLQKLAAKKIRDITKEQQAARLDMVEELAARIDEISDGVVVNRQSTMLGGKAGMSKPMEIRVDTM
DPDDLMRPEHYEDTEESTSFRQEFQARKARQDQGLDVIAEGLSTLKDIAADINEELDKQEPLINEVDTKIDKAAADL
KNTNVKLKDTVTKLRSSRNFCIDLILIAIILGIAGSLYS
>A9SQ71|SYP71_PHYPA-PHYSCOMITRELLA
MSTIDLLTRVNGVIKKYEKYDTDNIRGPEIASHDYFLRLYKSIEDDLNGALKKAAEAESEKNRAVIATLNADLRRTKAA
LRSELPKLHKLAAKKVKGVPPEEILTRPNMALALSARIEEVPDGVSVSKKKVAKGTPLEIKIDNYSPEDILRQGVKDHS
VESKGFQEEFETRRKKQDEGFMAIEQGLNTLKDMAQDIGEELNKQEQLVDEADSKIDKAGSDLKSTNSRLKESLTA
MRSSRNFCVDVTLLVIILGIAAYLYKFVPAFSWMLHPPLHLTHCCELELNFAVLKLD
>U5GAJ4|SYP71_POPTR-POPULUS
MSVIDILTRVDAICNKYDKYDVEKQKDLNVSGDDAFARLYAAVDADIEAALQKAELASKEKSKASAVAINAELRRTK
ARLLEEVPKLQRLAIKKVKGLSIEELAARNDLVHALPDRIQAIPDGSSAALKQTGGWGSSAPRTEIKFDSDGQFDNEY
FQESETSSQFRQEYEMRKMKQDQGLSMISEGLDTLKNMAHDMNEELDRQVPLMDEIDTKVDKAAADLKNTNVR
LKDTVNQLRSSRNFCIDIVLLIIILGIAAYLYNVLKK
>B9IJ24|SYP71_POPTR-POPULUS
MSVIDILTRVDAICNKYDKYDIEKQKDLNVSGDDAFARLYAAIDSDIEAAHQKAELASKEKSKASAVAINAELRRTKA
RLLEEVPKLQRLAVKKVKGLSTEELAARNDLVLALPDRIQAIPDGTAAAPKQTGGWGTSAPRAEIKFDSDGQFDNEY
FQETETSSQFRQEYEMRKMKQDQGLDMISDGLDTLKNMAHDMNEEIDRQVPLMDEIDTKVDKAAADLKNTNV
RLKDTVNQLRSSRNFCIDIVLLIIILGIAAYLYNVLKK
>C5YV35|SYP71_SORBI-SORGHUM
MTVIDILTRVDAICQKYDKYDVDKLNGANVAGDDPFARLYASVDADINQCVEKAETAKQEKNRAAVVALNAEIRRT
KAKLIEEDLPKLQRLAVKKVKGLTREEIATRSDLVAALPDRIQSIPDGSSTATKKNGTWGGASGSRTGGAIKFDSTAD

GNFDDEYFKGTEESNQFRREYEMRRMKQDEGLDVIGEGLETLKNMASDMNEELDRQVPLMDEMDDKVDRANA
DLKNTNVRLKETVLQLRSSRNFCIDIILLCVILGIAAYLYNVLKK
>D7SKW3|SYP71_VITVI-VITIS
MSVIDIIFRVDQICKKFDKYDVDKQRDLNAYGDDAFARLYASVEANIESALHKSEISLIETNRAAAVALNAEIRRTKAR
LLEEVVKLQKLVLKKVKGLTKEELSIRNDLVLALPERIRAIPDGSMAGAKQTANWAASASHKNIMFDSSDGNFDSEF
FQQSEESSQFRQEYEMRRMKQASLDVISEGLDTLKNLANDMNEELDRQVPLIDEIDTKVDKATSDIKNTNVRLKET
VTKMRSSQNFCIDIILLCVILGIASYLYNVLK
>D7TQJ1|SYP71_VITVI-VITIS
MTVIDILTRVDAICKKYDKYDIDKQKDLNVSGDDAFARLYAVVEADIEAALQKADTASNEKNRASAVALNAEIRRTK
ARLLEEVPKLQRLAIKKVKGLSTEELAARNDLVLALPDRIQAIPDGAATAPKQTGGWAASASRTEIKFDSDGRFDSEY
FQQTEESSQFRQEYEMRKMNQDQGLDVIAEGLDTLKNMAHDMNEELDRQVPLMDEIDTKVDKATADLKNTNV
RLKDTVNQLRSSRNFCIDIILLCIILGIAAYLYNVLKK
>Q94KK6|SYP72_ARATH-ARABIDOPSIS
MPVIDIIFRVDEICKKYDKYDIDKHREIGASGDDAFSRLFTSIDSDIEAVLRKAELASTEKNRAAAVAMNAEVRRTKAR
LAEDVVKLQKLAVKKIKGLTREERESRCDLVIALADRLQAIPDGNEHGAKQANSDWGGASAPNKNIKFDMSEEDM
DDGFFQQSEESSQFRQEYEMRRKKQDEGLDIISEGLDALKNLARDMNEELDKQVPLMEEMETKVDGATSDLKNT
NVRLKKQLVQMRSSRNFCIDIILLCVILGIVSYIYNALN
>F4JEA3|SYP73_ARATH-ARABIDOPSIS
MGVIDLITRVDSICKKYEKYDINRQRDANVSGDDAFSRLYSAVEYALETVLQKTEDLSSETNKAKAVAMNAEIRRTK
ARLLEGIPKLQRLSLKKVKGLSKEELDARNDLVLSLRDKIEAIPESSAPVVGGWEASTSYSNIRFDTNVSDDRIGSEYFQ
PTGESDQFKQEYEMKRIKQDQGLDYIAEGLDTLKNMAQDINEELDRQEPLMDEIDTKIDKAATDLKSTNVRLKDTV
TKLRSSRNFCIDIILLCILLGIAAFIYNSVK
>Q9SD96|SNAP29_ARATH-ARABIDOPSIS
MAPKNSSWNPFDDEKEAAKSFSLNPFDDDDDDKEVEKRFTSSLKPSGGKENQTVQELESYAVYNSEETTKTVQGC
LKVAEEIRCDASKTLVMLNEQGDQITRTHQKTVDLDHHLSRGEKILGRLGGVFSRTWKPKKSRSITGPVITKGDSPK
RKVIDLKTREKLGLNPSLKPKSKTLPEAVDAYQKTQIAKQDEALTDLSALLGELKNMAVDMGTAIERQTNELDHLQD
NADELNYRVKQSNQRARYLLRK
>I1GSQ6|SNAP29_BRADI-BRACHYPODIUM
MPVSRAAKPVSSKPNPFDSDSESEFTSKPARASSSYSVDPSTKSQYKNGFHDSGGFENQSVQELESYAEYKADETT
QRVNDCLRLAENIREGATNTLITLHKQGEQINRTHETAANIDQDLSRSETLLGSLGGLFSKTWKPKKTRQIKGPAIITR
DDSFKRRANHLEQREKLGLSSSPREKLNSQKYSDPTNAMEKVQVEKDKQDNALSDLSDILGQLKGMALDMGSEID
RQTKAMDGLQDDVVELNSRVKGANQRARRLLWK
>I1IA62|SNAP29_BRADI-BRACHYPODIUM
MPVSKPSSSKPAPIDSDSDDDLVPKRPATKYTAPAGAKKQQYKDGFRDAGGLENQSVQELENYAAYKAEETTDTL
NGCLRIAENIREDASNTLITLNKQGEQISRTHDKAVEIDQDLAKGESLLNSLGGFFSKPWKPKKTRQIKGPAQVSRD
DSFKKKANRMEQRDKLGLSPRGKGNSRTYDDPTNAMDKVQVEKQKQDDALDDLSGVLGQLKGMAVDMGSELD
RQNQALDNLQDDVEELNSRMKGANQRARKLVAK
>A8I4X5|SNAP29_CHLRE-CHLAMYDOMONAS
MWGKKNASAAQVVRERKEEEVARNALFAGQEGEIVIDPTQLPVEEQTTDQLVRTAQQTHKETTQAAQRALKVIE
ESKQIQAETMKNLEDQNKKMWDIHTRMDDMNEELTYAEKLLSYMRRCCCCWLCDSCTGADPEEQRKRAWQRR
VAKGKTAVPAGRGQPPQQGRGGAGEKANGGGGGNGAGGKEQGGTYRNPDIRLPDEHRGVEMGLHEETRKQD
EVIDQIHAGLEHLLEGARGMHGELAAQNKELDALDDKAAATRDRINDVNKNSQLRNMARGKPKPSKEEPLVPGLP
SKNDVALAAAKRLAGV
>C6T803|SNAP29_SOYBN-GLYCINE
MFGSKKTPLRDAKPTSVDHAHNPFDSDEDTKDNKYNSSKKTLTNTNPFDDDHVNATGHSSSSSYGLSSTHRNRYK
NDFRDSGGLESQSVQELENYAVYKAEETTNSVNSCLKIAEEMREDATKTLVMLHQQGEQITRSHHVAADIDHDLS
RGEKLLGSLGGMFSKTWKPKKTGTITGPVVFGEDPVRKKGNHLEQREKLGLTSAPKGQSKSRTTLSEPSNVLEKVEV
EKGKQDDALSDLSDLLGELKGMAIDMGSEIGSQNQALDGFDNDMEKLIVRVNGANQRGRRLLGK
>I1JZK8|SNAP29_SOYBN-GLYCINE
MSGSKKSPLKVAKPSSAESWTNPFDSDDEGKDSKKYSSSRKTSSERALATLEVNTNPFDDDIDANKKSSSTSYAFQS
ANRNRYKNDFRDSGGLENQSVQELESYAVYKAEETTNSVNNCLKIAENIREDATKTLVTLEQQGEQITRSHHVAADI

DHDLSRGEKLLGSLGGLFSKTWKPKKTRAITGPVIVGDDPVRRKDNHLEQRDKLGLTSAPKGQSKLRTPPPEPTNAL
EKVEVEKKKQDDALSDLSDLLGELKDMAVDMGSEIERHNKALNHLYDDVDELNFRVKGANQRGRRLLGK
>I1KCY1|SNAP29_SOYBN-GLYCINE
MFGSKKTPLRDAKPSSVGHAHNPFDSDEETNDKKYNSSKKTLTNTNPFDDEVDVDATGHSSSSYGLSSSHRNRYK
SDFRDSGGLESQSVQELENYAVYKAEETTNSVNSCLKIAEEMREDATKTLVMLHHQGEQITRSHHVAADIDHDLSR
GEKLLGSLGGMFSKTWKPKKTGTITGPVVFGDDPVRRKGNHLEQREKLGLTSAPKGQSKSRPTLSEPSNALEKVEV
EKGKQDDALSDLSDLLGELKGMAIDMGSEIGRQNQALDGFDNDMEKLTVRVNGANQRSHRLLGK
>C6TJG5|SNAP29_SOYBN-GLYCINE
MFGSKKSPLKVAKPSSVESWTNPFDSNDEGMDTKKYSSSRKTSSERALTTLGVNTNPFDDGTDANKKSSSTLYGFQ
SANWNKYKNDFRDSGGLENQSVQELESYAVYKAEETTNSVTNCLKIAENIREEATQTLVTLHQQGEQITRSHHVAA
DIDHDLTRGEKLLGSLGGLFSKTWKPKKTRAITGPVIVGDDPVRRKGNHLEQREKLGLTSAPKGQSKLRSPPQEPTN
AFEKVEVEKNKQDDALSDLSDLLGELKGMAVDMGSEIERHNKALNHLYDDVDELNFRVIGANQRGRRLLGK
>Q6H766|SNAP29_ORYSJ-ORYZA
MPVSRGGKAASSKADPFDSDSDSDLVPKKKPGAYTAPSGAAKARYKDDFRDSGGLEQQSVQELENYAAYKAEETT
DALGGCLRIAENIREDAANTLVTLNKQGQQISRTHEKAVEIDQDLSKGESLLGSLGGFFSKPWKPKKTRQIKGPAHV
SDDSFKKKASHIEQREKLGLSPSGKSANRSYAEPTTAMEKVQVEKQKQDDALDDLSGVLGQLKGMACDMGSELD
RQNKALDDLQGDVDELNSRVKGANQRARKLIEK >A9SYL3|SNAP29_PHYPA-PHYSCOMITRELLA
PATENSMYRTLNMHDDKVPEPRGHWCCPTPYWRRGSRSLITAQMRSKFQKFSNSSVGSCPFVTANFIHTSTVTLS
TPICYNNLLRVAHQPARSSLSGQPLQTLAAVDVANYRACWYWKNSYLIIAAASQELRIGGMLIEGSGVHRADVRG
APFETAMAVGHCGKQSTNPFETSDSEVSYVNPFDDDDDALEVSSAHFASSASVPKEVNRGSYSCEEDGEDLIPVKV
ERTSDKVRHPLMEDDEALSDLSNLIGQLKNMSLLINSEISKQTEGLAHLVYDVEELNARVKGANVRGHQLRR
>A9T617|SNAP29_PHYPA-PHYSCOMITRELLA
MARRGPRNPFDDEDAYPKPPRGGSDGKKGVIKTQSADDDELFSYGNSRAKGGYRDGSGDHDDEFVNQAVADLE
KHAVKKSQETSDTLRNCLRVAEDTMAVGSQTLISLVDQGDQITRTHDKAVDIDQHLSRGEKLLGSLGGFFSKSWKP
TKTKKITGPMVGRVNQNNKESAEDREALGLNGTNEKKKSGSGSYHDKETFQGQINAERETQDDMLDDLSNVLSV
MKEMSMDMNKEIERQAPGIEHLREDVEELNRRTRDANIRGQRLLRR
>A9TIB5|SNAP29_PHYPA-PHYSCOMITRELLA
MARRAPRNPFDDEETYSKSRPDGSAGRKGVIKTQTADEDELFSYSSSKPKPGYNNGGGEDSEEFVNQAVLDLEKH
AVKKSQETTSTLKNCLRVAEDTMGIGAQTLISLHEQGVQIERTHEKAVHIDQHLSRGEKLLGSLGGVFSKSWKPKKT
KKITGPMVGRANNNNNNKESAEDREALGLNGSQAKKTSGSGSQHDRETFQGQIAAERETQDDMLDDLSNVLSQ
MKEMSMDMNTEIERQAPGIEHLHDDIQEVNARVKRANIRGQRLLRR
>B9I2W3|SNAP29_POPTR-POPULUS
MFGSKKSPLKISEHNKADPECPAPSRSNHFESENAVGTKKSLKPPRKTSSEPNLTSPNFRTNPFDNDEERGLSSSSTY
SLALSTRHKYKNDFLDLGGLENQPVQELENYAVYKAEETTKAVNGCLKIAEEMREGATTTLITLHQQGEQITKTHSV
AVEIDHDLSRGEKLLGSLGGMFSKTWKPKKNRAIRGPVITRDASPRRRGNHLEQREKLGLNLAPKERSSTKTPLPET
ANAFQKVEFEKSKQDDALSDLSNVIGELKNMAVDMGAEFDRQVGALDHVQDDVDELNSRLGGANKRGLHLLRK
>B9IE11|SNAP29_POPTR-POPULUS
MFGSKKSPLKISKHTRVDPECPAPSRPNPFDSDDEFDTKKTLKTSRKTSSEPNLTAPCFSTNPFDGDEERGSSASSTY
FLASAARQKYNNDFRDSGGLENQSVQELESYAVYKAEETTKSVNGCLKIAEDMREGATRTLITLHQQGEQITRTHD
AAVEIDHDLSRGEKLLGSLGGMFSKTWKPKKNRPITGPVITRDASPQRRGNQLEQREKLGLNPAPKGQSSMRTPLP
EPTNAFQKVELEKSKQDDSLSDLSNILGELKNMAVDMGTEIDRQTNSLDHLQNDVDELNYRVRGANQRGRRLLGK
>C5XTD7|SNAP29_SORBI-SORGHUM
MPLGGKPAASSKPNPFDSDSDSESNNKPAAKKSGAYQAPADAKKRYKDGFRDAGGLENQSVEELQHYAAYKAEE
TTDALEGCLRIAEDIKKDASDTLVTLHKQGEQISRTHEKAVEIDQDLSKSESLLGSLGGFFSKPWKPKKTKQIKGPAHV
SRDDSFKKKASRMEQRDKLGLSPRGKRDPRHYAEATDAMDKVQIEKKKQDDALDDLSGVLGQLKGMAVDMGSE
LDRQNEALDNLQGDVDELNSRVKGANQRARKLVAK
>F6GT32|SNAP29_VITVI-VITIS
MFGLKKSPLHKFAKHNSVDPGYPHSSSTNPFDSHDDLDGKKALKPSRRTSSEPMLTTQPNPNPFDDDVGKGSSSSS
SNFLLSSERNKYKNDFHDSGGIENQSVQELENYAVYKAEENTKMVNNCLKIAEDIREDATKTLVTLHQQGQQITRT
HMNAADIDHDLSRGEKLLGSLGGIFSKTWKPIKTRPITGPITTRDDLSYRKGSHMEQREKLGLAPAPKGRLATRTPP

PEPTNALQKVEVEHAKQDDTLSDLSNLLDELKYMAVDMGTEIESHNRALAPMEDDVDVLSIRIKDGNQRARRLLG
K
>Q9LMG8|SNAP30_ARATH-ARABIDOPSIS
MFGFFKSPGNNKLPNESSNNKGGTITAGRRTSSEPILITPDFDDDDKYKNGFNDSGGLQSQTTEELEKYAVYKAEET
TKGVNNCLKIAEDIRSDGARTLEMLHQQGEQINRTHEMAVDMDKDLSRGEKLLNNLGGMFSKPWKPKKTKNITG
PMITPDKPSKKSENHKEEREKLGLGAKGRSSSQPALDQPTNALQKVEQEKAKQDDGLSDLSDILGDLKSMAVDMG
SEIDKQNKALDHLGDDVDELNSRVQGANQRARHLLSK
>I1J5Y3|SNAP30_SOYBN-GLYCINE
MFGFRKTPAPTESDKKTTLTAEKRTASEPVLPVPKSKGNYFDYDDDDWGRKPSSSTASKDKDRYKNGFSNSGGLEN
QSVQELENYAVYKSEETTKSVNNCLRIAEDIRGDATRTLDMLHQQGEQITRTHNMVVDTEKDLSRGEKLLNNLGG
MFSKPWKPKKTREIQGPIITPDKPSKKNVHSKEDREKLGLAPLPKGRSAPTTPPNESSDAYQKVDYEKAKQDDALED
LSDILGDLKGMAISMGSELDKQNKALDHLADDVDELNSRVKGANQRARKLVG
>K7K7S4|SNAP30_SOYBN-GLYCINE
MFGFRKAPAPTESDKKTTLTAEKRTTSEPVLPVAKSKGNYFDDDDDDWGRKPSSSTASKDKDRYKNGFSNSGGLE
NQSVQELENYAVYKSEETTNSVNNCLRIAEDIRGDATRTLDMLHQQGEQITRTHNMVVDTEKDLSRGEKLLNNLG
GMFSKPWKPKKTREIQGPIITPDKPSKKNVHNKEDREKLGLAPLPKGRSAPTTPPNESSNAYQKVEHEKAKQDDAL
EDLSGILGDLKGMAIGMGSELDKQNKALDHLGDDVDELNSRVKGANQRARKLVG
>A9PB46|SNAP30_POPTR-POPULUS
MFGFRKSPANNKPTKQSSVNPPASFTSNPFDSDDESNAKQTLHHGKRTYSEPMLNVPNDAFDDRDGYKNDFRDS
GGLENQSVQELEKYATYKAEETTKSVNNCLRIAEDIRQDATRTLDMLHAQGEQITRTHNMAVDMDKDLGKGEKLL
NNLGGIFSKPWKPKKTRDITGPLITADKPSKLNQYNRGEREKLGLAPRGRSAPTTPPPEPTNALQKVEAEKGKQDD
ALSDLSNILGDLKGMAIDMGSELDSQNKSLDHLSDDVDELNSRVKGANQRARRLLGK
>A5BGC6|SNAP30_VITVI-VITIS
MMFGFMKSPMNKVFKHNAVDPGPGPSNPFDSDTENDSNQIHRDSKRAASEPTLPGLNFNIEDQYKNDFRNSGG
LENQSVQELENYAAYKAKETTKTVNNCLKIAEDIREDATNTLATLHQQGEQITRTHMMAADMDKDLSKGEKLLGN
LGGMFSRTWKPKKSKDITGPLITSEDSKGGPASLEQRQKLGLAPAPKGRTTSRAPGPEPTAAMQQVEGEMLKQD
DALSDLSNILGDLKNMAADMGSELDRQNKALDHLSDDVDELNARVKGANQRARRLLAK
>Q9S7P9|SNAP33_ARATH-ARABIDOPSIS
MFGLRKSPANLPKHNSVDLKSSKPNPFDSDDESDNKHTLNPSKRTTSEPSLADMTNPFGGERVQKGDSSSSKQSLF
SNSKYQYKNNFRDSGGIENQSVQELEGYAVYKAEETTKSVQGCLKVAEDIRSDATRTLVMLHDQGEQITRTHHKAV
EIDHDLSRGEKLLGSLGGMFSKTWKPKKTRPINGPVVTRDDSPTRRVNHLEKREKLGLNSAPRGQSRTREPLPESAD
AYQRVEMEKAKQDDGLSDLSDILGELKNMAVDMGSEIEKQNKGLDHLHDDVDELNFRVQQSNQRGRRLLGK
>Q94AU2|SEC22_ARATH-ARABIDOPSIS
MVKMTLIARVTDGLPLAEGLDDGRDLPDSDMYKQQVKALFKNLSRGQNDASRMSVETGPYVFHYIIEGRVCYLT
MCDRSYPKKLAFQYLEDLKNEFERVNGPNIETAARPYAFIKFDTFIQKTKKLYQDTRTQRNIAKLNDELYEVHQIMTR
NVQEVLGVGEKLDQVSEMSSRLTSESRIYADKAKDLNRQALIRKWAPVAIVFGVVFLLFWVKNKLW
>C6TGR3|SEC22_SOYBN-GLYCINE
MVKLTMIARVTDGLPLAEGLDDGRDLKDAEFYKQQVKALFKNLSRGHYEASRMSVETGPYVFHYIIEGRVCYLTMC
DRAYPKKLAFQYLEELRNEFERVNGSQIETAARPYAFIKFDTFMQKTKKLYQDTHTQRNIAKLNDELYEVHQIMTRN
VQEVLGVGEQLDQVSQMSSRLSSESRIYADKARDLNRQALIRKWAPVAIVFGVVFVLFWIKNKLW
>D7TYI6|SEC22_VITVI-VITIS
MFINSPPFSSFAFHILGCLQVKTRAKTNEQLSKISSNEEMKEVGSFYEICHSKLPSDSLSQLHSCRVVMMSKKTEHDV
LVRFPYRMSSCENFRGEASGGVNGIMYSALKKKWEMVGQQSLTYSYVVRGTGILNEYTEFTVDRVEDDFNKRCG
GEKVAVAVILDLNTGSDPKLKEHMQYCVDHPEGSTNLPKRRA
>F4KDI1|YKT61_ARATH-ARABIDOPSIS
MKITALLVLKCAPEASDPVILSNASDVSHFGYFQRSSVKEFVVFVGRTVASRTPPSQRQSVQHEGCAPFLILDLPGLC
PGFNFLRFYLIVHAYNRNGLCAVGFMDDHYPVRSAFSLLNQVLDEYQKSFGESWRSAKEDSNQPWPYLTEALNKF
QDPAEADKLLKIQRELDETKIILHKTIDSVLARGEKLDSLVEKSSDLSMASQMFYKQAKKTNSCCTIL
>I1HTQ7|YKT61_BRADI-BRACHYPODIUM

MKITAILVLKPSSSGAGGSSTSAGGGGPGSEAFVLANATDVSHFGYFQRGAAREFIVFVARTVAQRTQPGQRQSV
QHEEYKVHSHNRNGLCVVAFMDDHYPVRSAFSVLNKVLDEYQKAFGDSWKSATADSAQQWPFLTDALTKFQDP
AEADKLTKIQRDLDETKIILHKTIENVLERGERLDSLVEKSSDLSAASQMFYKQAKKTNSCCTIL
>A8J6T1|YKT61_CHLRE-CHLAMYDOMONAS
MKLVGVGLLTYKGDATPPAFIGMAVDVSNFGYFQRGAVREGIMFIARTITQRTAPGMRQTVKNEEYLCHVHVKD
NGVAGIVVADGEYPTTAAFSVIGKVLDDFMQQHANDDSWRTLEADSTLANPLLEAALTKYQDHTQADKIAKIQKD
LDETKIILHQTIDSVLKRGEKLDALVDKSNDLSLASQMFYKRARQTNSCCRFM
>I1L2D3|YKT61_SOYBN-GLYCINE
MKITALLVLKCTGEGSDPVILANASDVSHFGYFQRSSVKEFIVFVGRTVASRTPQGQRQSVQHEEYKVHTYNRNGL
CALGFMDDHYPIRSAFSLLNQVIDEYQKNFGESWRNAQEDSTQPWPYLNEALAKFQDPAEADKLMKIQRELDETK
IILHKTIDSVLARGEKLDSLVEKSSDLSAASQVLYLFHAFLAFGFNLFLHR
>C6SVB1|YKT61_SOYBN-GLYCINE
MKITALLVLKCTGEGSDPVILANASDVSHFGYFQRSSVKEFIVFVGRTVARRTPQGQRQSVQHEEYKVHTYNRNGL
CALGFMDDHYPIRSAFSLLNQVIDEYQKNFGESWRNAQEDSTQPWPYLNEALAKFQDPAEADKLMKIQRELDETK
IILHKTIDSVLARGEKLDSLVEKSSDLSAASQMFYKQAKKTNQCCTIL
>Q5N9F2|YKT61_ORYSJ-ORYZA
MKITALLVLKPSSSGGGASSSGGGGGSGGPEAVVLANATDVSHFGFFQRGAAREFIVFVARTVAQRTQPGQRQSV
QHEEYKVHSHNRNGLCAVAFMDDHYPVRSAFSLLNKVLDEYQKAFGDSWKAATKDATDAAQQWPFLTDALTKF
QDPAEADKLMKIQRDLDETKIILHKTIESVLQRGERLDSLVEKSSDLSAASQMFYKQAKKTNQCCTIL
>A9TB08|YKT61_PHYPA-PHYSCOMITRELLA
MKITALLVLKAPVAQGGESLMLANASDVSHFGFFQRQAAKEFILFASRTIAGRTPLGQRQTVEQDEYVVHCYNRSG
LCGLVFADKEYPLRSAFSVINKVLDEYQKTFGDSWKIKNTDSTDAWPFLPEALVKYQNPTEADKLAKIQKDLDETKVI
LHKTIESVLKRGENLDNLVDKSNDLSSASQMFYKQAKKANQCCTIL
>A9S7P6|YKT61_PHYPA-PHYSCOMITRELLA
FASRTISGRTPLGQRQSIEQDEYVVHCYNRSGLCGLVFADKEYPLRSAFSVINKVLDEYQRTFGDSWKIKNTDSTDA
WPYLPEALAKYQNPTEADKLAKIQKDLDETKVILHKTIESVLKRGENLDNLVDKSNDLSSASQMFYKQAKKANQCC
TIL
>B9HAR1|YKT61_POPTR-POPULUS
MKITALLVLKCNPEGSDPVILANAMDVSHFGYFQRSSVKEFIFFVGRTVAKRTPPGQRQSVQHEEYKVHTYNRNGL
CALGFMDDHYPVRSAFSLLNQVIDEYQKNFGESWRAAQADSTQPWPYLNEALTKFQDPAEADKLLKIQRELDETK
IILHKTIDSVLARGEKLDSLVEKSSDLSAASQMFYKQAKKTNQCCTIL
>A5BHS0|YKT61_VITVI-VITIS
MKITALLVLKCNPDGSDAIILANASDVGHFGYFQRSSVKEFIVFVGRTVAKRTPPSQRQSVQHEEYKVHSYNRNGLC
ALGFMDDHYPVRSAFSLLNQVIDEYQKNFGDSWRTVQADNTQPWPYLNEALAKFQDPAEADKLLKIQRELDETKI
ILHKTIDSVLARGEKLDSLVEKSSDLSTASQMFYKQAKKTNQCCSIL
>D7UAC6|YKT61_VITVI-VITIS
MKITALLVMKWQGDSIDPVILANACDVSQFGYFQRSGAKEFIVFVGRTVAKRTPPAQRQSVQHQEYKVHSYNRN
GLCALGFMDDNYPVRSAFSLLNKVLEEYQKNFGDSWRNAQADVTQPWPYLNEALTKFQDPAEADKLLKIQRDLD
ETKIILHMTIDSVLARGEKLDSLVEKSSDLSMASQMFYRQARKTNQCCTIL
>Q9LVM9|YKT62_ARATH-ARABIDOPSIS
MKITALLVLKCDPETREPVILANVSDLSQFGKFSFYRSNFEEFIVFIARTVARRTPPGQRQSVKHEEYKVHAYNINGLC
AVGFMDDHYPVRSAFSLLNQVLDVYQKDYGDTWRFENSSQPWPYLKEASDKFRDPAEADKLLKIQRELDETKIILH
KTIDGVLARGEKLDSLVEKSSELSLASKMFYKQAKKTNSCCTLL
>Q9ZRD6|YKT61_ARATH-ARABIDOPSIS
MKITALLVLKCAPEASDPVILSNASDVSHFGYFQRSSVKEFVVFVGRTVASRTPPSQRQSVQHEEYKVHAYNRNGLC
AVGFMDDHYPVRSAFSLLNQVLDEYQKSFGESWRSAKEDSNQPWPYLTEALNKFQDPAEADKLLKIQRELDETKII
LHKTIDSVLARGEKLDSLVEKSSDLSMASQMFYKQAKKTNSCCTIL
>O49377|VAMP711_ARATH-ARABIDOPSIS
MAILYALVARGTVVLSEFTATSTNASTIAKQILEKVPGDNDSNVSYSQDRYVFHVKRTDGLTVLCMAEETAGRRIPF
AFLEDIHQRFVRTYGRAVHTALAYAMNEEFSRVLSQQIDYYSNDPNADRINRIKGEMNQVRGVMIENIDKVLDRG
ERLELLVDKTANMQGNTFRFRKQARRFRSNVWWRNCKLTVLLILLLLVIIYIAVAFLCHGPTLPSCI

>I1H0J8|VAMP711_BRADI-BRACHYPODIUM
MAILYAVVARGTAVLAEHSAAATNAGAVARQVLERLPDGGADSHVSYTQDRYVFHAKRTDGITALCMADDAAGR
RIPFAFLEDIHGRFVKTYGRAALTALAYAMNDEFSRVLSQQMDYYSNDPSADRINRMRGEISQVRSVMIDNIDKVL
ERGDRLDLLVDKTANMQGNTVRFRRQARRYRSSVWWRNVKLTAALILLLLVIIYIALFFVCHGFTLPTCIR
>I1JIW7|VAMP711_SOYBN-GLYCINE
MGILYALVGRGSVVLAEFSGTTTNASAIARQILEKIPGNNDTHVSYSQDRYIFHVKRTDGLTVLCMADDTAGRRIPF
AFLEDIHQKFVRTYGRAVHSAQPYGMNEEFSRVLSQQMEYYSSDPNADRINRLKGEMSQVRNVMIENIDKVLDR
GDRLELLVDKTANMQGNTFRFRKQARRFRSTVWWRNVKLTIALIVVLLVIVYVVLAFVCHGPALPSCF
>I1LZR3|VAMP711_SOYBN-GLYCINE
MGILYGMVARGQVVLAEFSATQSNASVVAKQILSKINQGSDNNNDSNVSFSHDRYVFHVKRTDGLTVLCMADDA
FGRIAPFAFLEDIHKKFVKTYARAILSAPAYAMNDEFSRVLNQQMEYYSNDPNADRLNRLKGEMTQVRTVMIDNI
DKVLERGGRLELLVEKTSAMNSNSIRFKRQSRRYKNNLWWSNVRLTVALVIIFVIVVYIILAFICHGPLLSSCWR
>I1MU52|VAMP711_SOYBN-GLYCINE
MGILYGMVARGQVILAEFSATQSNASVVAKQILSKINQGSDDTNNDSNVSFSHDRYVFHVKRTDGLTVLCMADD
AFGRMIPFAFLEDIHKKFVKTYGRAILSAPAYAMNDEFSRVLNQQMEYYSNDPNADRLNRLKGEMTQVRTVMID
NIDKVLERGGRLEMLVEKTSAMNSNSIRFKRQSRRYKNNLWWSNVRLTAALIIIFFIVVYIILAFICHGPLLSSCRR
>Q9LWK1|VAMP711_ORYSJ-ORYZA
MAILYAVVARGTVVLAEHSAAATNAGAVARQVLERLPGGGADSHVSYTQDRYVFHAKRTDGITALCMADEAAGR
RIPFAFLEDIHGRFVKTYGRAALTSLAYGMNDEFSRVLSQQMDYYSNDPNADRINRMRGEISQVRTVMIDNIDKVL
ERGDRLDMLVDKTANMQGNTIRFKRQARRFRNTTWWRNVKLTIALIFLLTVIIYVVLVFMCHGFTLPTCIR
>A9PGC9|VAMP711_POPTR-POPULUS
MAILYALVARGSVVLAEFSSTATNASAIARQILDKIPGNDDSNVSYSQDRYIFHVKRTDGLAVLCMADETAGRRIPF
AFLEDIHQRFARTYGRAVITAQAYAMNDEFSRVLSQQMEYYTNDPNADRINRLKGEMSQVRNVMIENIDKVLER
GDRLELLVDKTANMQGNTFRFRKQARRFSSAVWWRNVKLTVALILLLLVIIYVVLAVVCHGVTLPTCRK
>A9P9E7|VAMP711_POPTR-POPULUS
MAILYALVARGSVVLAEFTSTATNASAIARQILDKIPGNDDSNVSYSQDRYIFHVKRTDGLTVLCMADETAGRRIPFA
FLEDIHQRFVRTYGRAVITAQAYAMNDEFSRVLSQQMEYYTNDPNADRINRLKGEMSQVRNVMIENIDKVLERG
DRLELLVDKTANMQGNTFRFRKQARRFRSTVWWRNVKLTVALILLLLVIIYVVLAFVCHGLTLPTCLK
>B9IMU4|VAMP711_POPTR-POPULUS
MGILYGMVARGPVVLAEFSSIAQTNANTVARQILDKIPRGNEDSNSSYSHDRYIFHVKRTDDLTVLCMADDATGRR
IPFEFLEDIHQRFVKTYGRAIHTSAPYAMNEEFSRIMSQQMDHFSNDPNADRLNRLQGEMSHVRSVMIDNIDKVL
QRGDRLALLVEKTSTLQGNTIRFRRQTQRFRNTQWWRNFKLKATLILFLLIFIYTVLALFCHGPSLHSCLK
>C5Z5C0|VAMP711_SORBI-SORGHUM
MAILYALVARGTVVLAEHSAAATNAGAIARQVLERLPDGGADSHVSYTQDRYVFHAKRTDGITALCMADDSAGRR
IPFAFLEDIHGRFVKTYGRAALTALAYAMNDEFSRVLSQQMDYYSNDPNADRINRMRGEINQVRSVMIDNIDKVL
ERGDRLELLVDKTANMQGNTVRFKRQARRFRNTTWWRNVKLTAALILLLLVIIYVVLVFMCHGFTLPTCIR
>D7SUW1|VAMP711_VITVI-VITIS
MTILYALVARGSLVLAEFSGTSTNASAIARQILEKIPGDNDSNVSYSQDRYIFHVKRTDGLTVLCMADDTAGRRIPFA
FLEDIHQRFVRTYGRAVHSAQAYAMNDDFSRVLSQQMEYYSNDPNADRINRLKGEMGQVRNVMIENIDKVLDR
GDRLELLVDKTANMQGNTFRFRKQARRFRSTVWWRNVKLTVALIIILLVIVYVILAFACHGLALPSCVK
>Q9SIQ9|VAMP712_ARATH-ARABIDOPSIS
MSILYALVARGTVVLAELSTTSTNASTIAKQILEKIPGNGDSHVSYSQDRYVFHVKRTDGLTVLCMADEDAGRRIPFS
FLEDIHQRFVRTYGRAIHSAQAYAMNDEFSRVLNQQIEYYSNDPNADTISRIKGEMNQVRDVMIENIDNILDRGER
LELLVDKTANMQGNTFRFRKQTRRFNNTVWWRNCKLTLLLILVLLVIIYIGVAFACHGPTLPSCV
>Q9LFP1|VAMP713_ARATH-ARABIDOPSIS
MAIIFALVARGTVVLSEFSATSTNASSISKQILEKLPGNDSDSHMSYSQDRYIFHVKRTDGLTVLCMADETAGRNIPF
AFLDDIHQRFVKTYGRAIHSAQAYSMNDEFSRVLSQQMEFYSNDPNADRMSRIKGEMSQVRNVMIENIDKVLDR
GERLELLVDKTENMQGNTFRFRKQARRYRTIMWWRNVKLTIALILVLALVVYIAMAFVCHGPSLPSCFK
>Q9FMR5|VAMP714_ARATH-ARABIDOPSIS

MAIVYAVVARGTVVLAEFSAVTGNTGAVVRRILEKLSPEISDERLCFSQDRYIFHILRSDGLTFLCMANDTFGRRVPF
SYLEEIHMRFMKNYGKVAHNAPAYAMNDEFSRVLHQQMEFFSSNPSVDTLNRVRGEVSEIRSVMVENIEKIMER
GDRIELLVDKTATMQDSSFHFRKQSKRLRRALWMKNAKLLVLLTCLIVFLLYIIIASFCGGITLPSCRS
>I1I088|VAMP714_BRADI-BRACHYPODIUM
MAIVYALVARGTVVLAEFAAVTGNAGAVARRILEKLPSEAEARLCFAQDRYIFHVLRSPADGLTFLCMANDTFGRRI
PFIYLEDIQMRFMKNYGRVAHSALAYAMNDEFSRVLHQQMEFFSSNPSADTLNRLRGEVSEIHTVMVDNIEKILDR
GDRISLLVDKTSTMQDSSFHFRKQSKRLRRALWMKNAKLLAVLTAVIVLLLYLIIAAFCGGLSLSSCRS
>A8J924|VAMP714_CHLRE-CHLAMYDOMONAS
MAQAQKILFSLVARGQVVLAENRVTGNVNVIAVRILEKLPHEDTRVSYTQERFMFHVMVSDGITYMAVAEEGFGR
RIPFAFLDDVRGRFVAMYGVAAKEAVAYEYNTEFSRVLAERAAHFSDPSADAINRVKGELQEVKHIMIENIEKVLER
GERLDLLVDKTEGLQQVSLAFRREARRLKHTMWWKNAKLWVLVCAATALLIYFILGMVCGFTLKRC
>C6TME8|VAMP714_SOYBN-GLYCINE
MAIFYALVARGTVVLAEFSAVTGNTGAVSRRILEKLPAESDSRLCFSQDRYIFHILRSDGITYVCMANDTFGRRIPFSY
LEDIQMRFMKNYSRVANYAPAYAMNDEFSRVLHQQMEFFSSNASADTLNRVRGEVGEIRTIMVDNIEKILERGDR
IELLVDKTATMQDSAFHFRKQSKRLRRALWMKNFKLLALLTCFIVIVLYLIIAACCGGISLPSCRS
>Q33B02|VAMP714_ORYSJ-ORYZA
MAIVYAVVARGTVVLAEFSAVSGNAGAVARRILEKLPPDAESRLCFAQDRYIFHVLRSPPPAAADGLTFLCMANDT
FGRRIPFLYLEDIQMRFIKNYGRIAHNALAYAMNDEFSRVLHQQMEYFSSNPSADTLNRLRGEVSEIHTVMVDNIE
KILDRGERISLLVDKTSTMQDSAFHFRKQSRRLRRALWMKNAKLLAVLTAVIVLLLYLIIAAFCGGLSLPSCRS
>A9THE7|VAMP714_PHYPA-PHYSCOMITRELLA
MTILYALVARGTVLLAEFSAASGNGSTIARRILEKIPPGGDSRVSYSQDRHIFHIMKADGLTFLCMASDSFGRRIPFSY
LEDIHMRFMKNYGKVASTALAYAMNDEFSRVLHQQMEYFSRNPNADTINRVKGEIAEVRAVMVENIDKVLERGD
RIELLVDKTSTIQDNTFRFKKQSRRLRQAMWMKNAKLLATLTCIIIVFLYIIISFFCGGIFLRGCRS
>A9ST40|VAMP714_PHYPA-PHYSCOMITRELLA
MTILYALVARGTVVLAEFSAASGNSSTIARRILEKIPPGGDSRVSYSQDRHIFHIMKADGLTFLCMASDTFGRRIPFSY
LEDIHMRFMKSYGKVASTALAYAMNDEFSRVLHQQMEYFSSNPNADTINRVKGEIAEVRAVMVENIDKVLERGD
RIELLVDKTSTIQDNTFRFKKQSRQLKQAMWMKNAKLLATLTCLIIVLLYIIISFFCGGIFLRGCRG
>A9S6X1|VAMP714_PHYPA-PHYSCOMITRELLA
MAILYALVARGTVVLAEFSAASGNASTIARRILEKLPPGGDSRVSYSQDRHIFHILKADGLTFLCMATDSFGRRIPFAY
LEDIHMRFMKNYGKVASTALAYTMNDEFSRVLHQQMEYFSTNKQADSINRVKGEIVEVRAVMVENIDKVLERGD
RIELLVDKTATIQDNTFRFKKQSRRLRQAMWIKNAKLLATLTCLIIVLLYIIISFFCGGIFLRSCRG
>B9GI08|VAMP714_POPTR-POPULUS
MAIIYAVVARGTVVLSEFSAVTGNTGAVARRILEKLPSEADSRLCFSQDRYIFHILRSDGLTFLCMANDTFGRRIPFTY
LEDIHMRFMKNYGRVAHYAPAYAMNDEFSRVLHQQMEFFSSNPSADTLSRVRGEVGEIRTIMVENIEKILERGDRI
ELLVDKTATMQDGAFHFKKQSKGLRRALWMKNAKLLALLTCVIVLLLYIIIAACCGGITLPSCRS
>B9HPW6|VAMP714_POPTR-POPULUS
MAIIYAVVARGTVVLSEFSAVTGNSGAVARRILEKLPSEADSRLCFSQDRYIFHILRSDGLTYLCMANDTFGRRIPFSY
LEDIQMRFMKNYGRVAHHAPAYAVNDEFSRVLHQQMEFFSSNPSADTLSRVRGEVGEIRTIMVENIEKILERGDRI
ELLVDKTATMQDGAFHFKKQSKRLRRALWMKNAKLLALLTCVIVLLLYIIIAACCGGITLPSCRS
>C5YLC8|VAMP714_SORBI-SORGHUM
MAIVYALVARGTVVLAEFAAVSGNAGAVARRILEKLPPDAESRLCFAQDRYIFHVLRSDAAGITFLCMANDTFGRRI
PFLYLEDIQMRFMKNYGRVAHSALAYAMNDEFSRVLHQQMEFFSSNPSADTLNRLRGEVSEIHTVMVDNIEKILD
RGDRISLLVDKTSTMQDSSFHFRKQSKRLRRALWMKNAKLLVVLTVVIVVLLYLIISAFCGGLSLSSCRS
>D7TCR8|VAMP714_VITVI-VITIS
MAIVYALVARGTVVLSEFSAVSGNTGAVARRILEKLPAEADSRLCFSQDRYIFHILRADGLAFLCMANDTFGRRIPFS
YLEDIQMRFMKNYGKVAHYAPAYAMNDEFSRVLHQQMEFFSSNPSADTLNRVRGEVGEIRTIMVENIEKILERGD
RIELLVDKTATMQDSSFHFKKQSKRLRRALWMKNAKLLALLTCLIVVLLYIIIAACCGGITLPSCRS
>Q9ZTW3|VAMP721_ARATH-ARABIDOPSIS
MAQQSLIYSFVARGTVILVEFTDFKGNFTSIAAQCLQKLPSSNNKFTYNCDGHTFNYLVEDGFTYCVVAVDSAGRQI
PMSFLERVKEDFNKRYGGGKAATAQANSLNKEFGSKLKEHMQYCMDHPDEISKLAKVKAQVSEVKGVMMENIEK
VLDRGEKIELLVDKTENLRSQAQDFRTTGTQMRRKMWLQNMKIKLIVLAIIIALILIIVLSVCHGFKC

>P47192|VAMP722_ARATH-ARABIDOPSIS
MAQQSLIYSFVARGTVILVEFTDFKGNFTSIAAQCLQKLPSSNNKFTYNCDGHTFNYLVENGFTYCVVAVDSAGRQI
PMAFLERVKEDFNKRYGGGKAATAQANSLNKEFGSKLKEHMQYCMDHPDEISKLAKVKAQVSEVKGVMMENIE
KVLDRGEKIELLVDKTENLRSQAQDFRTQGTQMRRKMWFQNMKIKLIVLAIIIALILIIILSICGGFNCGK
>I1KMA2|VAMP721_SOYBN-GLYCINE
MGQDQNQRSLIYAFVSRGTVILAEYTEFSGNFNTIAFQCLQKLPASNNKFTYNCDGHTFNYLVDNEFTYCVVADESI
GRQVPVAFLERAKDDFVAKYGGGKATTAAANSLNKEFGSKLKEHMQYCVEHPEEISKLAKVKAQVSEVKDVMME
NIEKVLDRGEKIELLVDKTENLHHQAQDFRNSGTKIRRKMWLQNMKIKLIVLAILIALILIIVLSVCRGFNCGK
>I1L0A9|VAMP721_SOYBN-GLYCINE
MGQKSLIYAFVSRGTVILAEYTEFSGNFNSIAFQCLQKLPATNNKFTYNCDAHTFNYLVDNGYTYCVVADESIGRQL
PMAFLERVKDEFVSKYGGGKAATAPANSLNKEFGPKLREHMQYCVDHPEEISKLAKVKAQVSEVKGVMMENIEK
VLDRGDKIELLVDKTENLHNQAQDFRTSGTRIRRKMWLQNMKIKLIVLGILIALILIIVLSVCRGFNCGK
>A9P970|VAMP721_POPTR-POPULUS
MNQKSLIYAFVSRGTVILAEFTEFSGNFNSIAFQCLQKLPATNNKFTYNCDGHTFNYLADNGFTYCVVADESAGRQ
VPMAFLERVKDDFVSKYGGGKAATAQANGLNKEFGPKLKEHMKYCADHPEEISKLAKVKAQVSEVKGVMMENIE
KVLDRGEKIELLVDKTENLHSQAQDFRSQGTQIRRKMWLQNMKVKLIVLGILIALILIIVLSVCKGFNCGK
>B9ICK8|VAMP721_POPTR-POPULUS
MSQKSLIYAFVSRGTVILADYTEFSGNFNSIAFQCLQKLPATNNKFTYNCDGHTFNYLVDNGFTYCVVAAESAGRQ
VPIAFLERVKDDFVTKYGGGKAATAQANGLNKEFGPKLKEHMQYCADHPEEISKLAKVKAQVSEVKGVMMENIEK
VLDRGEKIELLVDKTENLHQQAQDFRSQGTQIRRKMWLQNMKVKLIVLGILIVLILIIVLSICKGFNC
>A5BQJ0|VAMP721_VITVI-VITIS
MGQKSLIYAFVARGTVILAEYTEFSGNFNSIAFQCLQKLPATSNKFTYNCDAHTFTYLLDNGYTYCVVADESVGRQV
PMAFLERIRDDFVARYGGEKAATAPANSLNKDFSSKLKEHMQYCVDHPEEISKLAKVKDQVSEVKGVMMENIEKV
LDRGEKIELLVDKTHNLHEQAQDFRSAGTKIRRKMWLQNMKIKLIVLGILVALILIIVLSVCHGFNCGK
>D7U3G1|VAMP721_VITVI-VITIS
MGQQSLIYSFVARGTVILAEFTEFSGNFTSIAAQCLQKLPATNNKFTYNCDGHTFNYLVEDGYTYCVVAVEAVGRQI
PIAFLERVKEDFTKRYGGGKAATAVANSLNKEFGPKLKEQMQYCVDHPEEISKLAKVKAQVSEVKGVMMENIEKVL
DRGEKIELLVDKTENLRSQAQDFRQQGTKMRRKMWLQNMKIKLIVLGILIALILIIVLSICGGFNC
>B9DH97|VAMP722_ARATH-ARABIDOPSIS
MAQQSLIYSFVARGTVILVEFTDFKGNFTSIAAQCLQKLPSSNNKFTYNCDGHTFNYLVENGFSESKYCSISYCVVAV
DSAGRQIPMAFLERVKEDFNKRYGGGKAATAQANSLNKEFGSKLKEHMQYCMDHPDEISKLAKVKAQVSEVKGV
MMENIEKVLDRGEKIELLVDKTENLRSQAQDFRTQGTQMRRKMWFQNMKIKLIVLAIIIALILIIILSICGGFNCGK
>Q8VY69|VAMP723_ARATH-ARABIDOPSIS
MAQQSLFYSFIARGTVILVEFTDFKGNFTSVAAQYLENLPSSNNKFTYNCDGHTFNDLVENGFTYCVVAVDSAGREI
PMAFLERVKEDFYKRYGGEKAATDQANSLNKEFGSNLKEHMQYCMDHPDEISNLAKAKAQVSEVKSLMMENIEK
VLARGVICEMLGSSESQPQAFYIKRTQMKRKKWFQNMKIKLIVLAIIIALILIIILSVCGGFNCGK
>O23429|VAMP724_ARATH-ARABIDOPSIS
MGQESFIYSFVARGTMILAEYTEFTGNFPSIAAQCLQKLPSSSNSKFTYNCDHHTFNFLVEDGYAYCVVAKDSLSKQI
SIAFLERVKADFKKRYGGGKASTAIAKSLNKEFGPVMKEHMNYIVDHAEEIEKLIKVKAQVSEVKSIMLENIDKAIDR
GENLTVLTDKTENLRSQAQEYKKQGTQVRRKLWYQNMKIKLVVLGILLLLVLIIWISVCHGFNCTD
>I1H2E7|VAMP724_BRADI-BRACHYPODIUM
MASPAPKEGGGKGEWLIYAFVARGIAVLAEYTEFTGNFPALAAQCLQRLPAGSANPGSMPARLSYGCDGHTFNFL
LDRGYAYCVVAKESVPKNLTVAFLERMKDDFMKRYGGGKADTALAKSLNKDYGPVIKQHMQYVLDHSDEIEKTLK
VQAQVSEVKNIMLNNIEKTLDRGEKLTELQDKTSDLCSQAQEFKKQGVKIRRKTWLQSMKLKLVILGILLLLVIIVWV
SVCQGFDCTKH
>I1KN03|VAMP724_SOYBN-GLYCINE
MSQESFIYSFVARGTMVLAEYTEFTGNFPAIAAQCLQKLPSSNNKFTYNCDHHTFNFLVEDGYAYCVVAKESVSKQI
SIAFLERVKADFKKRYGGGKADTAVAKSLNKEFGPVMKEHMKYIIDHAEEIEKLIKVKAQVSEVKSIMLENIDKAIDR
GENLTILADKTETLHSQAQEFKKQGTHVRRKMWYQNMKIKLVVLGILLLLVLVIWLSVCHGFNCAN
>I1L108|VAMP724_SOYBN-GLYCINE

MSQESFIYSFVARGTMLLAEYTEFTGNFPAIAAQCLQKLPSSNNKFTYNCDHHTFNFLVEDGYAYCVVAKESVSKQI
SIAFLERVKADFKKRYGGGKADTAIAKSLNKEFGPVMKEHMKYIINHAEEIVKLIKVKAQVSEVKSIMLENIDKALDR
GENLTILADKTEALRSHAQDFRKQGTQVRRKMWYQNMKIKLVVLGILLVLVLVIWLSICGGFDCTNWQ
>I1MGK7|VAMP724_SOYBN-GLYCINE
MSQESFIYSFVARGTMVLAEYTEFTGNFPAIAAQCLQKLPSSNNKFTYNCDHHTFNFLVEDGYAYCVVAKESVSKQI
SIAFLERVKADFKKRYGGGKADTAIAKSLNKEFGPVMKEHMKYIIDHAEEIVKLIKVKAQVSEVKSIMLENIDKAIDRG
ENLTILADKTETLRSQAQDFRKQGTQVRRKMWYQNMKIKLVVLGILLFLVLVIWLSICGGFDCTN
>Q8H3D2|VAMP724_ORYSJ-ORYZA
MASPPGKKGEGGGDGGGGKAEWLIYAFVARGTAVLAEYTEFTGNFPALAAQCLQRLPASGGGGSGGGAPARFSY
ACDGHTFNFLLHRGYAYCVVAKESVPKNVSVAFLERLKDDFMKRYGGGKADTALAKSLNKEYGPVIKQHMQYVLD
HSEEIEKTLKVQAQVSEVKNIMLENIEKTLGRGEKLSELQDKTSDLQSQAQEFKKKGVKIRRKTWLQNMKIKLVVLGI
LLLLVIIVWVSVCQGFDCTKH
>U7E0Y2|VAMP724_POPTR-POPULUS
MSQESFIYSFVARGTMILAEYTEFTGNFPAIATQCLQKLPSSNDKFTYNCDHHTFNFLVEDGYAYCVVAKETVSKQIS
IAFLERMKADFKKRYGGGKADTAAAKSLNKEFGPIMKEHMKYIIDHAEEIEKLIKVKAQVSEVKSIMLGNIDKAIDRG
EAIATLADKTETLRDQAQAYKKQGTQIRRKMWYQNMKIKLVVLGVLLILVLIIWLSICHGFDCSN
>D7T6E9|VAMP724_VITVI-VITIS
MSQESFIYSFVARGTMVLAEYTEFTGNFPAIATQCLQRLPSANNKFTYNCDHHTFNFLVEDGYAYCVVAKESVGKQ
VSIAFLERMKADFKKRYGGGKADTATAKSLNKDFGPIMKEHMQYIIDHAEEIEKLLKVKAQVSEVKSIMLENIDKTLE
RGENLTILADKTEDLRSQAQQFKKQGSQVRRKMWFQNMKIKLVVLGILLILALVIWVSICHGFNCSK
>O48850|VAMP725_ARATH-ARABIDOPSIS
MDRSVVPISLAPFQFLLVFWIFLTSVHTNPNKQQKTVVSLSLWWNSKNRIRGCVWFFFLLRVTRTMGQQNLIYSFV
ARGTVILVEYTEFKGNFTAVAAQCLQKLPSSNNKFTYNCDGHTFNYLVENGFTYCVVAVESVGRQIPMAFLERVKE
DFNKRYGGGKATTAQANSLNREFGSKLKEHMQYCVDHPDEISKLAKVKAQVTEVKGVMMENIEKVLDRGEKIELL
VDKTENLRSQAQDFRTQGTKIRRKMWFENMKIKLIVLGIIITLILIIILSVCGGFKCT
>F4J7T4|VAMP725_ARATH-ARABIDOPSIS
MVVDRNGYNYLTQQLEQRVLVLLAHPEEISKLAKVKALVTKMKGVMMENIEKALDRSEKIKILVDLRSKYSNLPFPS
YGQEDIITPGTKITRKMWFQNMKFKLIVLGTSSSRFVLITERRRRLR
>I1H2V0|VAMP725_BRADI-BRACHYPODIUM
MGQQSLIYAFVARGTVVLAEYTEFTGNFTTIAAQCLQKLPASNNKFTYNCDGHTFNYLVEDGFTYCVVAVESVGRQ
TPIAFLDRVKDDFTKRYGGGKAATAGASSLNREFGSKLKEHMQYCVDNPEEINKLAKVKAQVSEVKGVMMENIEK
VLDRGEKIELLVDKTENLRSQAQDFRQQGTKVRRKMWLQNMKIKLIVLGIIIALILIIILSVCHGFKCNK
>I1KZB1|VAMP725_SOYBN-GLYCINE
MGQQSLIYSFVARGTVILAEYTEFTGNFTGVAAQCLQKLPSSNNKFTYNCDGHTFNYLVDNGFTYCVVAVESAGRQ
IPIAFLERIKEDFTKKYAGGKAATAAAQSLNREFGPKLKEQMQYCVDHPEEISKLAKVKAQVSEVKGVMMENIEKVL
DRGEKIELLVDKTENLRSQAQDFRQQGTKIRRKMWFQNMKIKLIVLGIIIALILIIVLSVCGGFNCGK
>B9H095|VAMP725_POPTR-POPULUS
MGQQSLIYSFVARGTVILAEYTEFKGNFTGIAAQCLQKLPASNNKFTYNCDGHTFNYLVEDGFTYCVVAVESAGRQI
PIAFLERVKEDFNKRYGGGKAATAVANSLNREFGSKLKEHMQYCVDHPEEISKLAKVKAQVSEVKGVMMENIEKV
LDRGEKIELLVDKTENLRSQAQDFRQQGTKMRRKMWIQNMKMKLIVLGIIIALILIIVLSVCHGFNC
>C5XCJ4|VAMP725_SORBI-SORGHUM
MGQQSLIYAFVARGTVVLAEYTEFTGNFTTIAAQCLQKLPASNNKFTYNCDGHTFNYLVEDGFTYCVVAVESVGRQ
IPIAFLDRVKEDFTKKYGGGKAATAAANSLNREFGSKLKEHMQYCVDHPEEVSKLAKVKAQVSEVKGVMMENIEK
VLDRGEKIELLVDKTENLRSQAQDFRQQGTKVRRKMWLQNMKIKLIVLGIIIALILIIILSVCHGFKCGSK
>D7UDM2|VAMP725_VITVI-VITIS
MVGQQSLIYSFVARGTVILAEYTEFTGNFTSIAAQCLQKLPASNNKFTYNCDGHTFNYLVENGFTYCVVAVESAGR
QIPIAFLERVKDDFNKRYGGGKAATAVANGLNKEFGPKLKEHMQYCVDHPEEISKLAKVKAQVSEVKGVMMENIE
KVLDRGEKIELLVDKTENLRSQAQDFRQQGTKMRRKMWMQNMKIKLIVLGIIIALILIIVLSICHGFKC
>Q9MAS5|VAMP726_ARATH-ARABIDOPSIS

MGQQSLIYSFVARGTVILAEYTEFKGNFTSVAAQCLQKLPSSNNKFTYNCDGHTFNYLADNGFTYCVVVIESAGRQI
PMAFLERVKEDFNKRYGGGKASTAKANSLNKEFGSKLKEHMQYCADHPEEISKLSKVKAQVTEVKGVMMENIEKV
LDRGEKIELLVDKTENLRSQAQDFRTQGTKMKRKLWFENMKIKLIVFGIIVALILIIILSVCHGFKCT
>Q9M376|VAMP727_ARATH-ARABIDOPSIS
MSQKGLIYSFVAKGTVVLAEHTPYSGNFSTIAVQCLQKLPTNSSKYTYSCDGHTFNFLVDNGFVFLVVADESTGRSV
PFVFLERVKEDFKKRYEASIKNDERHPLADEDEDDDLFGDRFSVAYNLDREFGPILKEHMQYCMSHPEEMSKLSKLK
AQITEVKGIMMDNIEKVLDRGEKIELLVDKTENLQFQADSFQRQGRQLRRKMWLQSLQMKLMVAGAVFSFILIV
WVVACGGFKCSS
>F4J7U3|VAMP727_ARATH-ARABIDOPSIS
MTLSTALDRSEKIELLVDKIREPLFTGTRFQSARNENDKMWFQNMKIKLIVLGIIIFLSVNSHRHHRVNSHNSHIALQ
VDGKLFPFSSGKSPVQLTNPGDHTLLFQTPKGIIEICPRTPFKPRSAVFVTLIN
>I1I9H0|VAMP727_BRADI-BRACHYPODIUM
MNGGSSKQTLIYSFVAKGSVVLAEHTAFSGNFSTIAVQCLQKLPSNSTRSTYSCDGHTFNFLVDRGFVFLVVAEEAV
GRSVPFVFLERVKEDFMQRYGSSIDEEGQHPLADDADEDDFLFEDRFSIAYNLDREFGPRLKDHMQYCINHPEEISK
LSKVKSHLSEVKGIMMDNIEKILDRGEKIELLVGKTENLQSQADSFHRHGRELRRKMWLQNLRFKLMVGGGVAFLI
LILWLMVCKGFKC
>A8IT48|VAMP727_CHLRE-CHLAMYDOMONAS
MPLIYSSVSQGTVTLAEYAAFSGNFGAVAKDYLEKAGKNEGKFTFNVDGHTFNFLNRGGFTYLVVADEAYGRAIPS
AFLDKMASEFAMKFADKAAGAKEGGLNGSFGKQLKSMMEHATQYPEEYSKVASVQKKVDEVKGIMTENIEKVLA
RGEKLELLTDKTENLMNEADRFQRTGRTLRRKMWWQNCKMKIVVALAVILLAVVIFLLVCFSGGNCLK
>A8IT75|VAMP727_CHLRE-CHLAMYDOMONAS
MPLIYAFVARGTTVLAEYTPYSGNFNTVAIECLQKLANPEPKFTIACDRHTFNFLVANGFTYLVVADEAYGRQIPFAF
LERVRDEFEEKYAEKARTAAALSMDRTFGPRLKSHMEYCMDHPEEISKIAAVQKKVNEVKDVMVENIEKVLERGEK
IELLVDKTDDLRNQAEQFQKKGRQLRNKMWWQNCRMKLIVLFAILLLAVVIFLLVCFTGGKNCTK
>A8IT73|VAMP727_CHLRE-CHLAMYDOMONAS
MPLVYSCISLLHGVTLAEYAAFAGNFGAVAKEYLARTTGEGKLSYAVDGHTFTVLCRGGFVFLVAADEATGKTIPSA
FVDKVADEFTSKYADKAAGLAGKEGGLQSSFGKQLKSTMEHATQYPEEYSKVASVQKKVDEVKGIMTENIDKVLA
RGEKLELLTDKTENLMFESDRFVRTGRALRRRMWMQNCKMKIVVALAVILLAVVIFLLVCFSGGNCLK
>A8IT49|VAMP727_CHLRE-CHLAMYDOMONAS
MPLVYVSVCQGSVALAEYAGFQGNFAVVARDYLDKATKLEGKSRYEVDGHSLNFLNRGGFTYLVIASVDSGVALPS
AFLDKVEAEFRAKYGAGLQLGAAAGSLNATFGKQLKQLTENATQHPEEFSKVAAVQKKVDEAKAVMVDNIDAVL
KRGEKLEQIQEKTEDLMAEADRFRDGAVRVKRKLWWQNCKMKIVVALAVILLAVVIFLLVCFSGGNCLK
>A8J9R8|VAMP727_CHLRE-CHLAMYDOMONAS
MPTRKPQEPRKAIPAPQPVAPPETPRVHKKAGKRIVLAVLKLRRHLQHKFKKLVTEGRPTVPAPAPAIPPLPPPEALE
DRISHLTLKVEEVTAAARELTAQVLARGENLEVLCEKAEQLLQASQDFGKRCKRLKGMPWWARLATAGAVTVAAV
VCAVWGVRRGAPPFGRVSPPGGRVFFTRSRRYDRMHFWPAGRLMEKQ
>A8J9S0|VAMP727_CHLRE-CHLAMYDOMONAS
MPTHKAQEPRAARPEPQPGAPPETLGIPKKVVKKIVLSVLKLRRRLQRKFKKLVTAVRPVEPAAPAAPAIAPPLPPPL
PPPLPPPLPPPLSRPRPPPGGEEDRLSRLAMKQVEEVTAVARQRTDELLARGEKLEDLNELAEQLQHESQEFERRCR
RLKGIPWWARLAAAGAAVAAVVVACAVWGAGHPPRLAARRRRNSRVSMMNY
>I1LF04|VAMP727_SOYBN-GLYCINE
MSQRGLIYSFVAKGTVVLAEHTQYTGNFSTIAVQCLQKLPSNSSKYTYSCDGHTFNFLLDNGFVFLVVADESIGRSV
PFVFLERVKDDFMKRYGASIKNDGAHPLADDEDDDDLFEDRFSIAYNLDREFGPALKEHMQYCMNHPEEMSKLSK
LKAQITEVKGIMMDNIEKVLDRGEKIELLVDKTENLQFQADSFQRQGRQLRRKMWLQNLQMKLMVGGGILILIIIL
WVIACGGFKC
>I1NFE1|VAMP727_SOYBN-GLYCINE
MSQRGLIYSFVAKGTVVLAEHTQYTGNFSTIAVQCLQKLPSNSSKYTYSCDGHTFNFLLDTGFVFLVVADESAGRSV
PFVFLERVKDDFMKRYGASIKNEGAHPLADDDDDDDLFEDRFSIAYNLDREFGPALKEHMQYCMNHPEEMSKLSK
LKAQITEVKGIMMDNIEKVLDRGEKIELLVDKTENLQFQVCIIIIILIKHLYLSFLNKSFVYPKETLVFESTWPNSPVILLM
LLYFPFKFRLTASRGRAGS
>Q6YZI8|VAMP727_ORYSJ-ORYZA

MNGNKQSLIYSFVAKGSVVLAEHTAFSGNFSTIAVQCLQKLPPNTSKSTYSCDGHTFNFLVDRGFVFLVVADEAVG
RSVPFVFLDRVKEDFMQRYGSSIDEEGQHPLADDADDDDFLLEDRFSIAYNLDREFGPRLKDHMLYCINHPEEISKL
SKVKAHLTEVKGIMMDNIEKILERGEKIELLVGKTETLQSQADSFHRHGRELRRKMWLQNLRFKLMVGGAVAALIL
FLWLIICGGFKC
>A9TFG8|VAMP727_PHYPA-PHYSCOMITRELLA
MGTQSLIYSFVARGSTVLAEYTAFSGNFSTIAVQCLQKLPPNNNKFTYTCDRHTFNYLVEEGYTYLVVADEEFGRQIP
FAFLERVKEDFKRRYAGGKADSAIANSLDKEFGPKLKDHMQYCVDHPDEMNKISKIKSQVAEVKGIMMDNIEKVL
DRGEKIELLVDKTENLRFQADNFQRQGKQLRRKMWFQNMKVKLIVLAIIIVIIIIIWLSICRGFTCSNR
>A9TGQ9|VAMP727_PHYPA-PHYSCOMITRELLA
MEEGLIYSFVSRGTTVLAEYASVSGNSNRIAAQCLAKLPGGNNKHTYVCDRHTFNFLVEDGFTFLAVADEDFSRQIA
FAFLDRVKNDFQHRYQGGRADLAVTYSLNAEFGPRLKEHMDFVAANPEEIKKMSKIKSQVAEVKEIMMVNIEKLL
DRNERIDLLVGKTDDLHSNAHVFEKQGNQIRRRAWCAHFKLKLLVLVLIIIVAFIIYLSICRDFICHNPGMPGTTPANV
PPAE
>A9TMZ3|VAMP727_PHYPA-PHYSCOMITRELLA
MGDANLIYSLVSRGTTVLAEYTSFAGNFSQIAMQCLVKLPAANNKHTYVMDRHTFNFLVQDGFTYLVVAEEDFGR
QIPFAFLDRVKDDFKHRYQGGKADLAVSHSLDAEFGPRLKEHMDFCERNPEEIRKMSKIKSQVAEVKGIMMENID
KVLVRNEKIDLLVDRTSHLQSDAHNFQRQGKKIRYKLWCQNYRLKLLVLVLIIIVAFIIYLSICRGFVCYNPGVPGTPPA
PGTPPGQGL
>A9SDL2|VAMP727_PHYPA-PHYSCOMITRELLA
MGDARLIYSFVARGTTVLAEHAIYAGNFSQIAVQCLLKLPAGTSKQTYVMDRHTFNFFVENGFTFLVVAEEALGRLI
PFAFLERVKDDFKHHYQGGRADLAVSHSLDAEFGPKLKEHMDFCMENPEEIKKISRIKSQVAEVKGIMMENIDKVL
DRSDKIDLLVDRTTHLQSSAAEYQRAGVRIRRRLWWQHFRLKLLVLLLIVVVAFIIYLSICRGFICHNPAVPGTPPAPG
TPPGPL
>B9HKP0|VAMP727_POPTR-POPULUS
MSSQRGLIYSFVAKGNVVLAEHTSYSGNFSTVAVQCLQKLPSNSSKYTYSCDGHTFNFLIDNGFVFLAVADESAGRG
LPFVFLERVKDDFKQRYSASIKNEAHPLADDDDDDDLFEDRFSVAYNLDREFGPRLKEHMQYCLNHPEEISKLSKLK
AQITEVKGIMMDNIEKVLDRGERIELLVDKTENLQFQADSFQRQGRQLRRKMWLQNLQMKLMVGGGVLVVILIL
WFVACGGFKC
>A9PGA3|VAMP727_POPTR-POPULUS
MSSQKGLIYSFVAKGNVVLAEHTSYSGNFSTIAVQCLQKLPSNSSKYTYSSDGHTFNFLIDNGFVFLVVADESVGRG
VSFVFLERVKDDFNQRYGASIKNEAHPLADDDDDDDLFEDRFSIAYNLDREFGPRLKEHMQYCVNHPEEISKLSKLK
AQITEVKGIMMDNIDKVLDRGERIELLVDKTDNLSFQADSFQRQGRELRRKMWLQNLKVKLVLGGTVLALIVIVWI
SVCGGFKC
>D7TNZ0|VAMP727_VITVI-VITIS
MSQKGLIYSFVAKGTVVLAEHTSFSGNFSTIAVQCLQKLPSNSSKYTYSCDGHTFNFLIDSGFVFLVVADESAGRGAP
FVFLERVKDDFKQRYGGSIRSDGPHPLADEDDDDDLFEDRFSIAYNLDREFGPKLKEHMQYCMNHPEEISKLSKLK
AQITEVKGIMMDNIEKVLDRGERIELLVDKTENLQFQADSFQRQGRQLRRKMWLQNLRLKLMVGGIVLVLIIILWLI
ACKGFKC
>Q69WS1|VAMP725_ORYSJ-ORYZA
MGQQSLIYAFVARGTVVLAEYTEFTGNFTTIAAQCLQKLPASNNKFTYNCDGHTFNYLVEDGFTYCVVAVESVGRQ
IPIAFLDRVKEDFTKRYGGGKAATAAANSLNREFGSKLKEHMQYCVDHPEEISKLAKVKAQVSEVKGVMMENIEKV
LDRGEKIELLVDKTENLRSQAQDFRQQGTKVRRKMWLQNMKIKLIVLGIIIALILIIILSVCHGFKCK
