# Supplementary material for: Vesicle Transport in Plants: A Revised Phylogeny of SNARE Proteins
Source: Evol Bioinform Online. 2020 Oct 15;16:1176934320956575. doi: 10.1177/1176934320956575 (PMC7573729; doi:10.1177/1176934320956575)
Supplement: f763b0b446636_EvoBioRevSupplTable2_xyz46636386aed71 – Supplemental material for Vesicle Transport in Plants: A Revised Phylogeny of SNARE Proteins [file f763b0b446636_EvoBioRevSupplTable2_xyz46636386aed71.pdf]

Supplementary Table 2 Locus number and function of Arabidopsis R-SNAREs.

**Name**		**Locus No.**	**Function**	**Reference**
R	AtSEC22	At1g11890	Mediates both anterograde traffic between ER and Golgi and retrograde traffic within the Golgi apparatus. SEC22 is essential for gametophyte development and maintenance of Golgi-stack integrity.	Tsui et al. (2000); Sanderfoot and Raikhel, (2003); Bubeck et al. (2008); EI-Kasmi et al. (2011)
	AtYKT61	At5g58060	Mediates many different trafficking events in the cell.	McNew et al. (1997); Tsui et al. (2000); Bubeck et al. (2008)
	AtYKT62	At5g58180		
	AtVAMP711	At4g32150	Regulates ABA-mediated inhibition of PM H^{+}-ATPase activity and drought stress response by regulating stoma closure. Forms a SNARE complex with SYP22, VTI11 and SYP5.	Fujiwara et al. (2014); Xue et al. (2018)
	AtVAMP712	At2g25340	Induces stomatal closure in response to drought stress.	Leshem et al. (2010)
	AtVAMP713	At5g11150	Forms a SNARE complex with SYP22 to mediate vesicle transport.	Vieten et al. (2007); Ebine et al. (2011); Fujiwara et al. (2014)
	AtVAMP714	At5g22360	Interacts with SYP121 and SYP22 to mediate vesicle transport from the Golgi apparatus to the vacuole. VAMP714 is required for the exocytic localization of PIN proteins to the plasmamembrane via the Golgi, and for polar auxin transport.	Szponarski et al. (2003); Fujiwara et al. (2014); Cao et al. (2019); Gu et al. (2019)
	AtVAMP721	At1g04750	Forms a SNARE core complex with SYP121 to drive secretory pathways. VAMP721 and VAMP722 interact with the same K+ channels and that this interaction suppresses channel activity, and VAMP721 assembles with SYP121 to coordinate K+ channel gating during SNARE assembly and vesicle fusion. Involved in cell	Kwon et al. (2008); Zhang et al. (2011); Kanik et al. (2013); Yun et al. (2013); Zhang et al. (2015, 2017); Zhang et al. (2017); Uemura et al. (2019)
	AtVAMP722	At2g33120		

			plate formation.	
	AtVAMP723	At2g33110	Involved in secretion, but specific functions are not clear.	Kwon et al. (2008)
	AtVAMP724	At4g15780		
	AtVAMP725	At2g32670		
	AtVAMP726	At1g04760		
	AtVAMP727	At3g54300	Forms a complex with SYP22, VTI11, SYP121 and SYP51 to drive membrane fusion and vesicle transport pathways between PVCs and vacuoles. VAMP727 and SYP22 mediate the BRI trafficking to PM.	Ebine et al. (2008, 2011); Zhang et al. (2017); Takemoto et al. (2018); Uemura et al. (2019); Zhang et al (2019)
	AtVAMP728	At3g24890	Specific function is not clear.	Kwon et al. (2008)
