# Supplementary material for: Vesicle Transport in Plants: A Revised Phylogeny of SNARE Proteins
Source: Evol Bioinform Online. 2020 Oct 15;16:1176934320956575. doi: 10.1177/1176934320956575 (PMC7573729; doi:10.1177/1176934320956575)
Supplement: Supplementary_Figures_1-5_Gu_et_al. – Supplemental material for Vesicle Transport in Plants: A Revised Phylogeny of SNARE Proteins [file Supplementary_Figures_1-5_Gu_et_al..pdf]

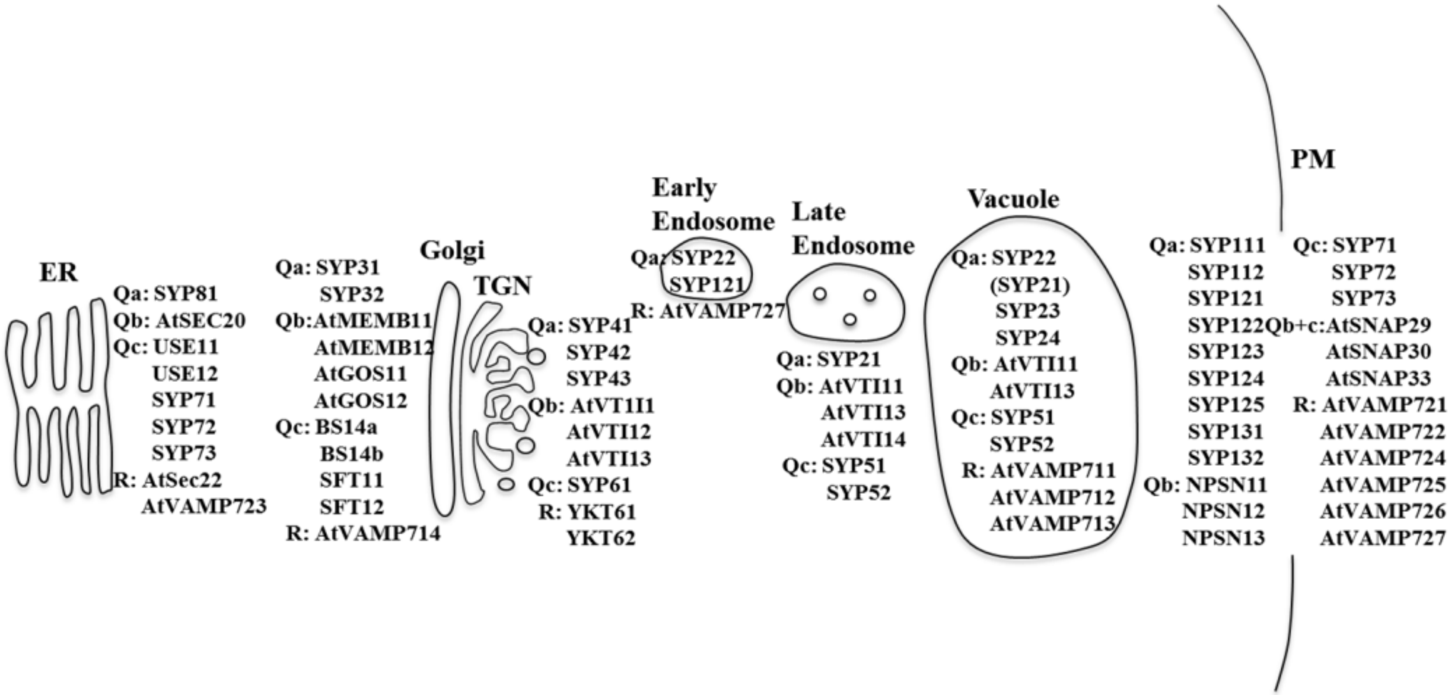

Suppl Fig. 1. Intracellular localization of SNARE proteins in *Arabidopsis*

Adapted from Uemura et al.[10]

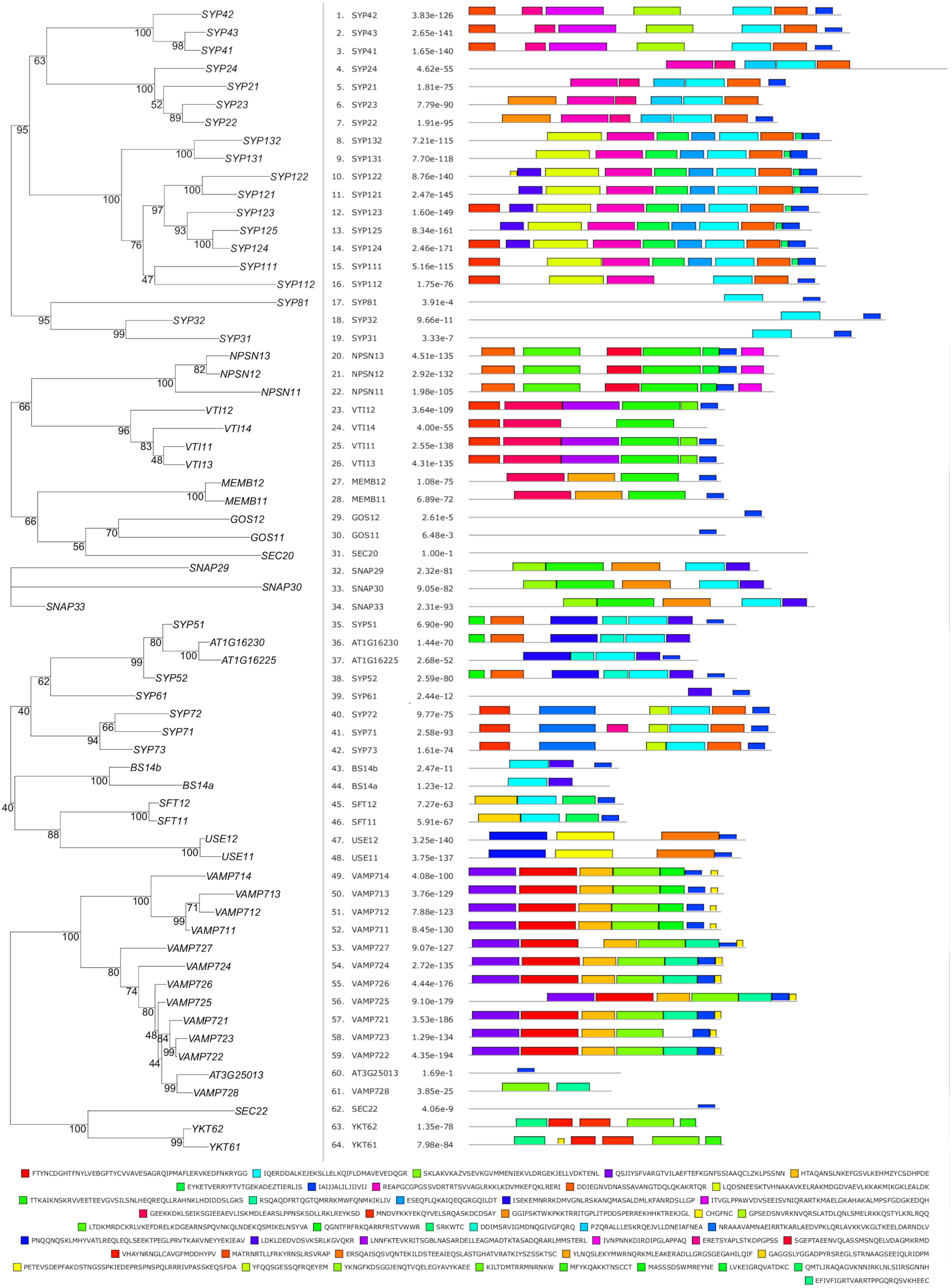

1. SYP42 3.83e-126
2. SYP43 2.65e-141
3. SYP41 1.65e-140
4. SYP24 4.62e-55
5. SYP21 1.81e-75
6. SYP23 7.79e-90
7. SYP22 1.91e-95
8. SYP132 7.21e-115
9. SYP131 7.70e-118
10. SYP122 8.76e-140
11. SYP121 2.47e-145
12. SYP123 1.60e-149
13. SYP125 8.34e-161
14. SYP124 2.46e-171
15. SYP111 5.16e-115
16. SYP112 1.75e-76
17. SYP81 3.91e-4
18. SYP32 9.66e-11
19. SYP31 3.33e-7
20. NPSN13 4.51e-135
21. NPSN12 2.92e-132
22. NPSN11 1.98e-105
23. VTI12 3.64e-109
24. VTI14 4.00e-55
25. VTI11 2.55e-138
26. VTI13 4.31e-135
27. MEMB12 1.08e-75
28. MEMB11 6.89e-72
29. GOS12 2.61e-5
30. GOS11 6.48e-3
31. SEC20 1.00e-1
32. SNAP29 2.32e-81
33. SNAP30 9.05e-82
34. SNAP33 2.31e-93
35. SYP51 6.90e-90
36. AT1G16230 1.44e-70
37. AT1G16225 2.68e-52
38. SYP52 2.59e-80
39. SYP61 2.44e-12
40. SYP72 9.77e-75
41. SYP71 2.58e-93
42. SYP73 1.61e-74
43. BS14b 2.47e-11
44. BS14a 1.23e-12
45. SFT12 7.27e-63
46. SFT11 5.91e-67
47. USE12 3.25e-140
48. USE11 3.75e-137
49. VAMP714 4.08e-100
50. VAMP713 3.76e-129
51. VAMP712 7.88e-123
52. VAMP711 8.45e-130
53. VAMP727 9.07e-127
54. VAMP724 2.72e-135
55. VAMP726 4.44e-176
56. VAMP725 9.10e-179
57. VAMP721 3.53e-186
58. VAMP723 1.29e-134
59. VAMP722 4.35e-194
60. AT3G25013 1.69e-1
61. VAMP728 3.85e-25
62. SEC22 4.06e-9
63. YKT62 1.35e-78
64. YKT61 7.98e-84

Suppl. Figure 2. MEME suite output showing 46 protein domains identified across 64 Arabidopsis SNARE genes.

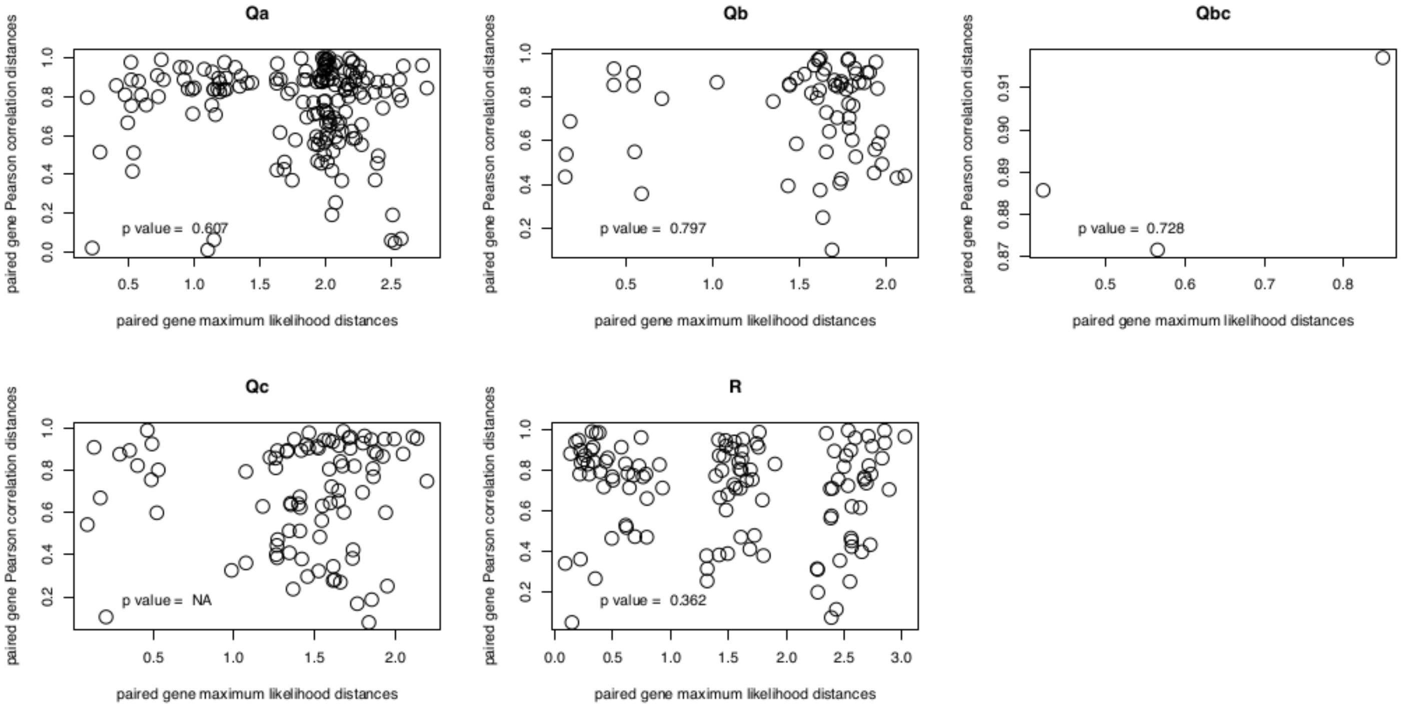

Suppl. Fig. 3. Within-gene class paired-gene maximum likelihood genetic distances versus Pearson correlation distances.

The results of Mantel tests are shown in each subpanel.

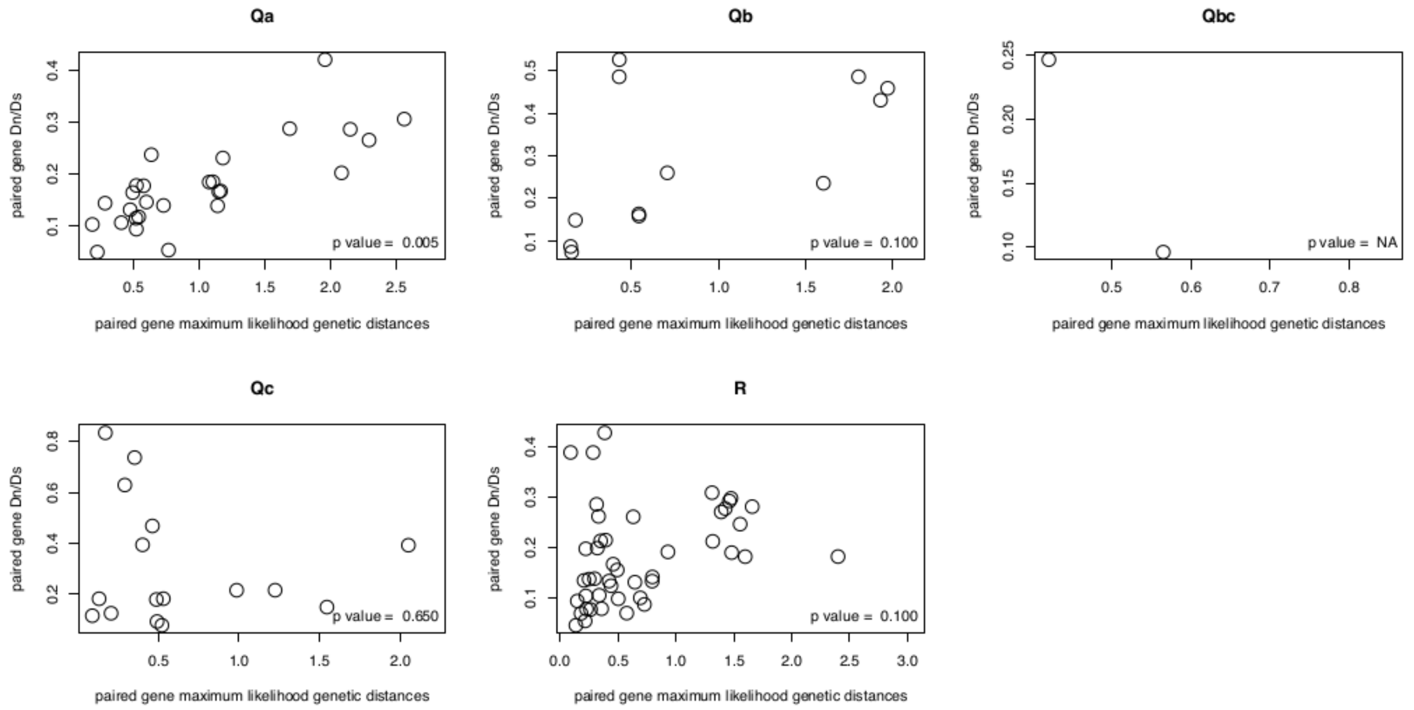

Suppl. Fig. 4. Within-gene class paired-gene non-synonymous to synonymous base change ratios (Dn/Ds) versus Pearson correlation distances.

The results of Mantel tests are shown in each subpanel.

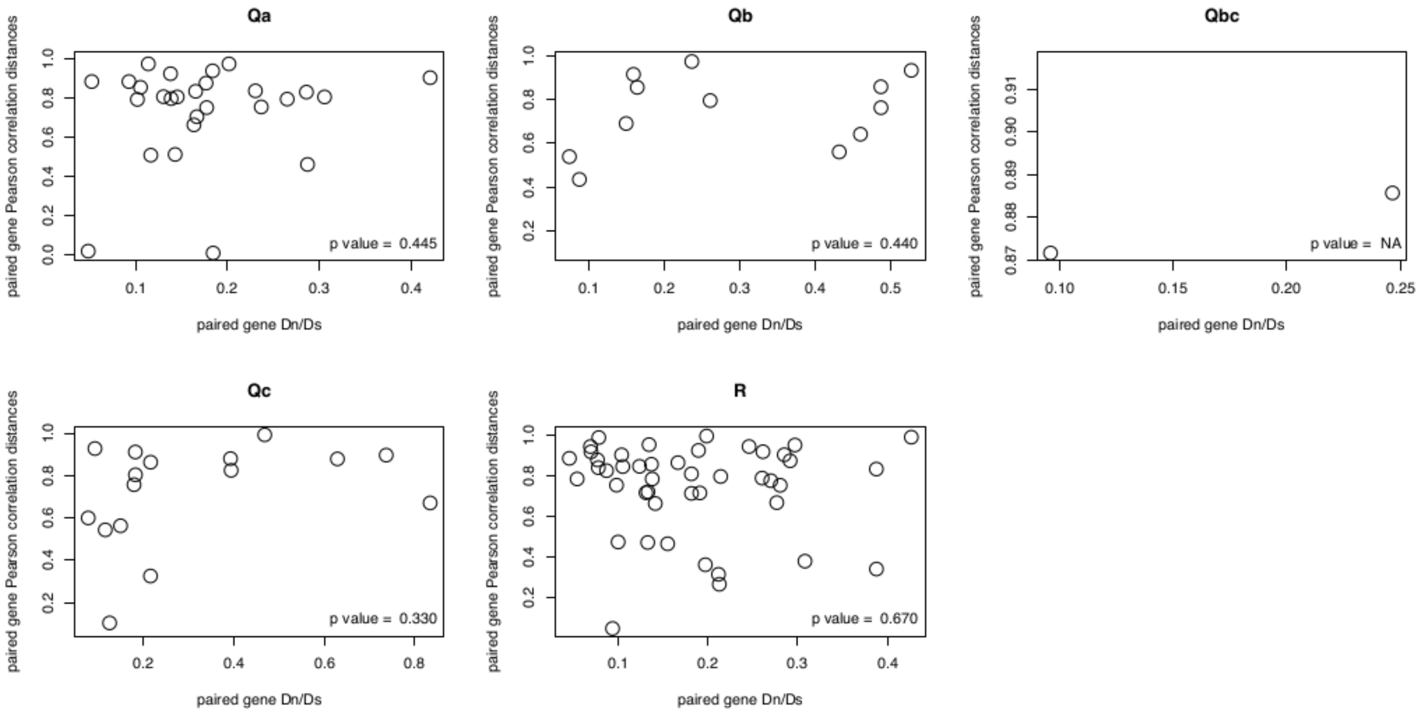

Suppl. Fig. 5. Within-gene class paired-gene maximum likelihood genetic distances versus non-synonymous to synonymous base change ratios (Dn/Ds).

The results of Mantel tests are shown in each subpanel.
